# Understanding the Underlying Molecular Mechanisms of Meiotic Arrest during In Vitro Spermatogenesis in Rat Prepubertal Testicular Tissue

**DOI:** 10.3390/ijms23115893

**Published:** 2022-05-24

**Authors:** Justine Saulnier, Frédéric Chalmel, Marion Delessard, Laura Moutard, Tony Pereira, François Fraissinet, Ludovic Dumont, Aurélie Rives-Feraille, Christine Rondanino, Nathalie Rives

**Affiliations:** 1 INSERM, U1239, Adrenal and Gonadal Pathophysiology Team, Laboratory of Neuroendocrine Endocrine and Germinal Differentiation and Communication, Rouen University Hospital, Rouen Normandy University, 76000 Rouen, France; justine.saulnier@live.fr (J.S.); marion.dufour@chu-rouen.fr (M.D.); laura.moutard@univ-rouen.fr (L.M.); ludovic.dumont1@univ-rouen.fr (L.D.); a.rives-feraille@chu-rouen.fr (A.R.-F.); christine.rondanino@univ-rouen.fr (C.R.); 2 Univ Rennes, Inserm, EHESP, IRSET (Institut de Recherche en Santé, Environnement et Travail)-UMR_S 1085, 35000 Rennes, France; frederic.chalmel@inserm.fr; 3 Department of General Biochemistry, Rouen University Hospital, 76000 Rouen, France; tony.pereira@chu-rouen.fr (T.P.); francois.fraissinet@chu-rouen.fr (F.F.)

**Keywords:** blood-testis barrier integrity, in vitro spermatogenesis, meiotic arrest, rat prepubertal testis, RNA-sequencing, steroidogenesis

## Abstract

In vitro spermatogenesis appears to be a promising approach to restore the fertility of childhood cancer survivors. The rat model has proven to be challenging, since germ cell maturation is arrested in organotypic cultures. Here, we report that, despite a meiotic entry, abnormal synaptonemal complexes were found in spermatocytes, and in vitro matured rat prepubertal testicular tissues displayed an immature phenotype. RNA-sequencing analyses highlighted up to 600 differentially expressed genes between in vitro and in vivo conditions, including genes involved in blood-testis barrier (BTB) formation and steroidogenesis. BTB integrity, the expression of two steroidogenic enzymes, and androgen receptors were indeed altered in vitro. Moreover, most of the top 10 predicted upstream regulators of deregulated genes were involved in inflammatory processes or immune cell recruitment. However, none of the three anti-inflammatory molecules tested in this study promoted meiotic progression. By analysing for the first time in vitro matured rat prepubertal testicular tissues at the molecular level, we uncovered the deregulation of several genes and revealed that defective BTB function, altered steroidogenic pathway, and probably inflammation, could be at the origin of meiotic arrest.

## 1. Introduction

With the improvement of biomedical research and clinical care, the survival of patients with childhood cancer has increased. The quality of life after cancer has therefore become a major issue in the management of these patients. Oncological treatments are mainly based on chemo- and radiotherapy, the gonadotoxicity of which can lead to gonadal dysfunction and infertility [1]. To preserve the future fertility of prepubertal boys, cryopreservation procedures of immature testicular fragments containing spermatogonial stem cells (SSC) have been developed [2]. Several fertility restoration approaches have also started to emerge [3] and are currently the object of research, such as (i) in vitro maturation in 3D cultures [4,5] or in organotypic cultures [6,7,8], and (ii) in vivo maturation by testicular tissue grafting [9,10] or SSC transplantation [11,12]. Among these different strategies, organotypic culture presents the advantage of maintaining the tissue architecture and cellular interactions that are necessary for germ cell maturation, as well as avoiding the potential reintroduction of residual tumour cells in cured patients. Using this approach, fertilizing spermatozoa have been obtained in vitro from mouse prepubertal testes [6,8]. The further development and optimization of organotypic culture procedures in animal models is essential before a human application can be envisaged. The rat model, the spermatogenesis of which has a duration that is intermediate between mice and humans (53 days vs. 36 and 74 days), and shows (as in humans) a germ cell maturation arrest in organotypic cultures [13,14,15,16,17,18], appears to be a relevant model for the improvement of in vitro culture conditions.

Spermatogenesis is an essential process by which haploid spermatozoa develop from SSC in the seminiferous tubules of the testis. Meiosis is the step during which genetic recombinations occur in diploid spermatocytes and, at the end of which, haploid spermatids are generated. A single round of DNA replication is followed by two consecutive rounds of nuclear meiotic divisions. The first meiotic division (meiosis I) is characterized by a prolonged prophase during which chromosome synapsis, crossovers, and homologous recombination take place [19], and by the segregation of homologous chromosomes. Synapsis and recombinations occur following the formation of a proteinaceous structure between the homologous chromosomes, called the synaptonemal complex [20]. Homologous recombinations require the generation of DNA double-strand breaks (DSBs). Most of the generated DSBs are involved in chromosome pairing and alignment [19,21]. The repair of DSBs is essential for stable homologous chromosome pairing and synapsis during prophase [22,23]. During meiosis II, sister chromatids separate within two haploid daughter cells.

Meiosis is therefore a complex process involving many genes, and the deregulation of its expression can be at the origin of meiotic arrest, as demonstrated in transgenic mice [24,25]. Numerous studies have investigated the causes of meiotic arrests in a fundamental or purely medical context [26,27,28,29]. Consequently, the development of high-throughput sequencing has led to the identification of several genes involved in male infertility [30]. Meiotic arrest could be due to chromosome abnormality, such as Y microdeletion or aneuploidy, or to gene mutations. For instance, a splice site mutation of the SYCE1 gene (encoding for Synaptonemal complex central element protein 1) leads to the disruption of synapsis in mice [31]. Some studies have highlighted the involvement of different checkpoints in cases of maturation arrest [32,33]. These checkpoints, such as the pachytene checkpoint, perform a fundamental role in meiotic progression. The pachytene checkpoint is involved in the repair of DSBs induced during meiotic recombination, and in the detection of asynapsis. It informs the cell of the persistence of DSBs on asynapsed chromosome fragments. Autosome asynapsis leads to a defect in sex chromosome inactivation and cell apoptosis [34]. It appears that mutations of genes involved in synapsis or silencing of sex chromosomes can induce an arrest of the cell cycle/meiosis at different stages [35,36]. However, chromosome and genetic alterations are not the only causes of meiotic arrests. Because spermatogenesis is finely regulated by different factors such as nutrients, temperature, and hormones, abrupt changes in the environment can affect its progression [37,38,39]. Indeed, an early deprivation of vitamin A in prepubertal mice leads to a disruption of the blood-testis barrier (BTB), an arrest in meiosis, and germ cell apoptosis [40,41]. In cases of cryptorchidism in humans, the maintenance of germ cells at body temperature induces the appearance of mutations, leading to germ cell alterations, testicular germ cell tumor formation, and progressive germ cell depletion [42]. Similarly, disruption of the androgen signaling pathway has been shown to lead to germ cell loss [43,44] or to azoospermia [45,46,47].

Our previous findings concerning in vitro maturation of prepubertal rat testicular tissue evidenced a blockage during meiosis I [16], as is also reported in human prepubertal testicular tissue [13]. The identification of gene sets or pathways that are specifically deregulated in vitro would contribute to a better understanding of the mechanisms involved in meiotic blockage, and consequently improve the in vitro maturation up to haploid germ cells. Transcriptomic approaches have proven to be useful in highlighting the downregulation of genes involved in the control of cell cycles such as *Igf-1* [48], or upregulation of genes related to immune response and the NFκB signaling pathway [49] in cultures of mouse prepubertal testes. Supplementations of culture media with IGF-1 [48] or an inhibitor of the TLR4-NFκB signaling pathway [49] led to a significant increase in the sperm yield and a reduction in the activation of macrophages, respectively. In addition, RNA sequencing (RNA-Seq) analysis has facilitated the identification of differentially expressed genes (DEGs) between in vitro and in vivo matured mouse ovarian tissues, thereby enabling the optimization of culture conditions and the successful completion of in vitro oogenesis [50].

The aim of the present study was therefore to understand the underlying mechanisms of meiotic arrest in organotypic cultures of prepubertal rat testicular tissue, using histological analyses and a bulk transcriptomic approach.

## 2. Results

### 2.1. Pre-Meiotic and Meiotic Germ Cells Survive in Cultured Prepubertal Rat Testicular Tissue but Do Not Achieve Their Differentiation

**Figure 1:**
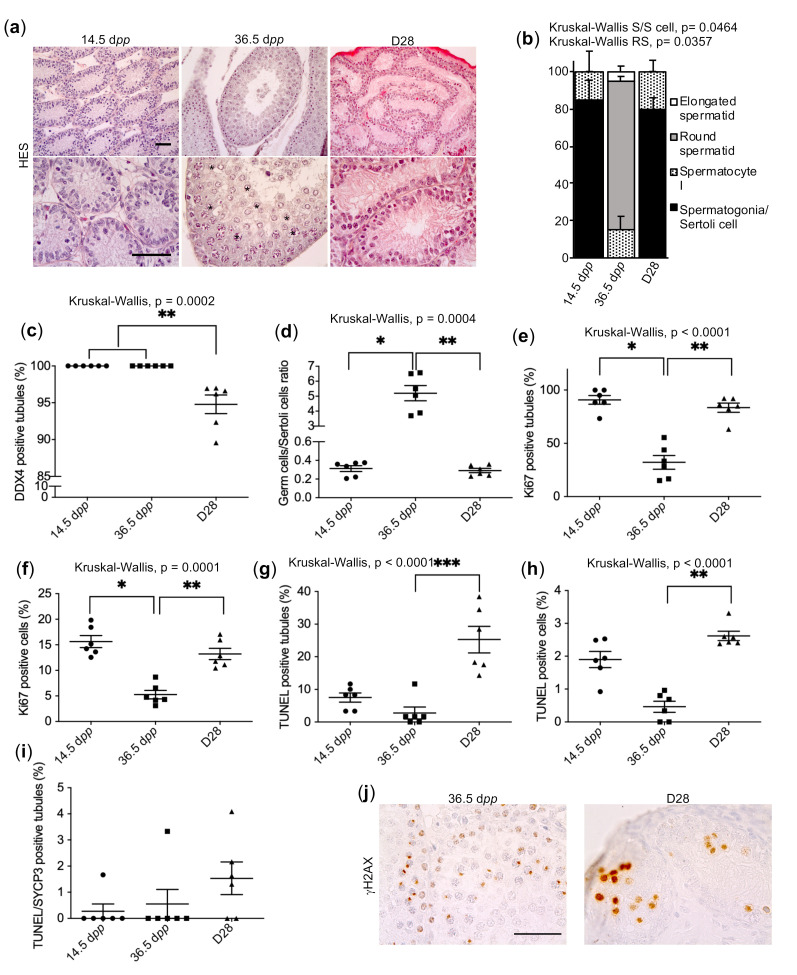
Histological and immunohistochemical evaluation of D28 in vitro cultured tissues sections. (**a**) HES staining of paraffin-embedded testicular tissue sections. Scale bar: 50 µm; Magnification: ×200 (upper panel) and ×500 (lower panel). Tissues cultured for 28 days (D28), tissues recovered from an age-matched in vivo control (36.5 dpp) and from an immature in vivo control (14.5 dpp) were analysed. Germ cells were observed in the adluminal compartment (*) only at 36.5 dpp. The most advanced stage of spermatogenesis (**b**) was evaluated (*n* = 3). DDX4 immunostaining was performed to determine the percentage of seminiferous tubules containing germ cells (**c**) and the intratubular germ cells/Sertoli cells ratio (**d**). (**e**) Percentage of Ki67-positive tubules and (**f**) Ki67-positive cells. (**g**) Percentage of TUNEL-positive tubules and (**h**) TUNEL-positive cells. (**i**) Percentage of SYCP3-positive cells (spermatocytes) presenting DNA fragmentation (TUNEL positive). (**j**) Immunohistochemical localization of γH2AX in paraffin-embedded testicular tissue sections from 36.5 dpp (left) and D28 cultured tissues (right). Indistinguishable XY bodies and a dispersed staining within the nuclei were observed in cultured tissues. Scale bar: 50 µm; Magnification: ×500. Unless otherwise, *n* = 6. For statistical analyses, Kruskal–Wallis tests followed by Dunn’s post-test were applied. Data are expressed as mean ± s.e.m. * *p* value < 0.05; ** *p* value < 0.01; *** *p* value < 0.001. dpp, days post-partum; S/S cell, Spermatogonia/Sertoli cell; HES, Hemalun eosin saffron; RS, Round spermatid.

As previously described [16], rat prepubertal testicular tissues were cultured on agarose gels for 28 days (D28), at which time round spermatids should be present in seminiferous tubules. In order to characterize and identify the origin of the meiotic arrest in in vitro cultured rat prepubertal testicular tissues, histological and immunohistochemical analyses were first performed and compared to age-matched in vivo controls (36.5 dpp) and prepubertal in vivo controls (14.5 dpp). In seminiferous tubule sections of in vivo rat testes, all the expected stages of spermatogenesis were observed, with meiotic germ cells present in the adluminal compartment of seminiferous tubules at 36.5 dpp. (Figure 1a,b). In sections of the D28 cultured tissues, an early meiotic arrest at the spermatocyte I stage was observed. The tubules remained quite empty and few spermatocytes I were observed (Figure 1a,b). The immunolabeling of DDX4, a protein expressed in the germline and involved in germline integrity, confirmed that the germ cell content was decreased after organotypic culture, with the presence of Sertoli cell-only tubules and a decrease in the germ cells/Sertoli cells ratio compared to age-matched in vivo controls. While a lower number of germ cells per tubule was observed in cultured tissues compared to the age-matched control, the number of Sertoli cells per tubule was similar between the two conditions (Figure 1c,f, Figure S1a–c,f). In cultured tissues, the nuclei of some germ cells appeared pyknotic. However, the germ cells/Sertoli cells ratios were similar between D28 cultured tissues and 14.5 dpp immature testes (Figure 1d, Figure S1a–c).

We next determined whether germ cell depletion in cultured tissues was the result of a decrease in proliferation or an increase in DNA damage. Ki67 immunostaining revealed that the proliferative activity of intratubular cells was maintained at the end of the culture process (Figure 1e,f, Figure S1g). The proliferative activity in D28 cultured tissues was closer to that of the immature 14.5 dpp tissues compared to the age-matched 36.5 dpp controls, suggesting that an impairment of tissue maturation may be a possible cause of the meiotic arrest. To evaluate the DNA integrity of the produced spermatocytes, we combined a TUNEL assay with SYCP3 immunostaining. As expected, an increased proportion of intratubular cells with fragmented DNA (TUNEL positive) was detected in cultured tissues, partially due to the culture conditions (Figure 1g,h). However, the proportion of tubules containing TUNEL-positive spermatocytes at D28 was not different from in vivo controls (Figure 1i). DSBs repair defects could lead to cell death [51]. However, the percentage of spermatocytes with DNA fragmentation was below 0.2% in cultured tissues (Figure S1d), which is insufficient to explain the cell maturation arrest.

**Figure 2:**
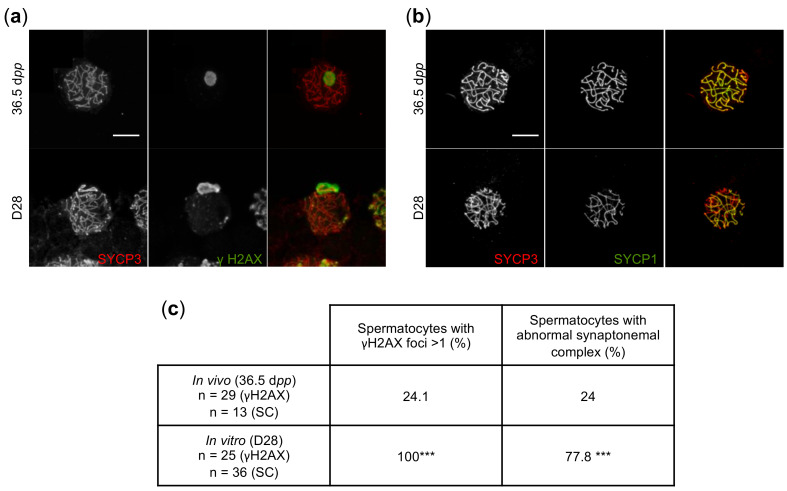
Immunofluorescence staining of meiotic spread preparations from D28 cultured tissues and 36.5 dpp controls. (**a**,**b**) γH2AX (**a**), SYCP1 (**b**), and SYCP3 (**a**,**b**) immunostaining. The upper and lower rows show a representative pachytene spermatocyte at 36.5 dpp and at D28, respectively. Scale bar: 10 μm, Magnification: ×630, Zoom 4. (**c**) Average percentage of spermatocytes with more than one γH2AX foci and with abnormal synaptonemal complex at 36.5 dpp (in vivo controls) and at D28. Cells were obtained from at least three different rats/culture repeats. For statistical analyses, a Chi^2^ test was applied *** *p* < 0.0001. Dpp—Days post-partum; SC—synaptonemal complex.

The formation of the XY body was then assessed by immunolabeling γH2AX, a protein involved in DSBs and transcriptional silencing of the XY body, on tissue sections (Figure 1j). When physiologically expressed, γH2AX is present at the zygotene stage on the chromosomal asynapsis region, whereas it becomes restricted and highly expressed in the XY body at the pachytene stage [52]. In D28 cultured tissues, arrested spermatocytes displayed an indistinguishable XY body and dispersed γH2AX staining within the nuclei, around the chromosomes (Figure 1j). To confirm this histological observation, meiotic chromosome spreads were prepared from D28 cultured tissues and age-matched in vivo controls. Using confocal microscopy, we found a γH2AX-positive XY body and a significantly higher number of γH2AX foci in the pachytene spermatocytes generated in vitro, which were localized on the chromosome axes, in comparison to the unique γH2AX-positive XY body detected in pachytene spermatocytes in vivo (Figure 2a,c, Videos S1, S2). Since the γH2AX increase could be due to asynapsis or the absence of DSBs repair [51], the integrity of the synaptonemal complex was investigated by performing double SYCP3/SYCP1 immunostaining. As previously hypothesized, asynapsis could be observed in many spermatocytes generated during culture, while some of them seemed to be correctly synapsed (Figure 2b,c, Videos S3, S4). An extensive absence of DSBs repair associated with a high level of asynapsis has previously been shown to be sufficient to induce the early apoptosis of pachytene spermatocytes [34].

### 2.2. In Vitro- and In Vivo-Matured Prepubertal Testicular Tissues Have Distinct Transcriptomic Profiles

In order to identify the molecular mechanisms involved in the spermatogenic arrest during organotypic culture, bulk RNA sequencing analyses were conducted on in vitro- and in vivo-matured testicular tissues. For each time point, three replicates were analysed, with three different testes for in vivo controls and twelve pooled cultured explants per replicate for the in vitro conditions. One 14.5 dpp testicular tissue, which displayed a different transcriptomic profile compared to the other two replicates, was excluded from the downstream analyses. 

**Figure 3:**
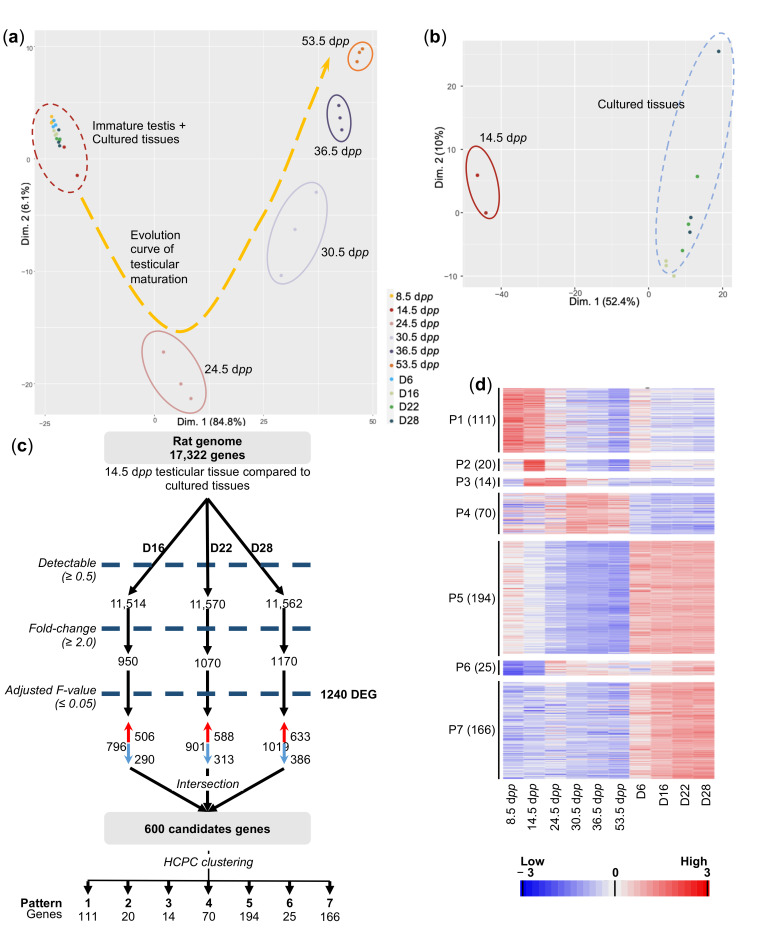
Transcriptomic analyses of in vitro and in vivo testicular tissues. (**a**) Scatter plot representing the position of each sample along the first two dimensions. Each point represents the average expression level of transcripts, one dot per replicate. The variability between samples is mainly explained by their age of development (Dimension 1, 84.8%). The different clusters, represented by colored circles, highlight the distance between each group of transcriptomes. An evolution curve of the transcriptome of maturing testicular tissues is represented in yellow. (**b**) PCA analysis conducted on the red dotted circle is shown in panel A, corresponding to cultured tissues and 14.5 dpp immature testes. The variability between groups is mainly explained by the origin of the tissue (in vivo vs. in vitro). All the in vitro-matured tissues display similar transcriptomic profiles. (**c**) Flowchart summarizing the filtration and clustering strategies. The numbers below the cluster ID indicate the genes falling into each cluster. (**d**) A false-color heatmap summarizes the seven patterns defining the global concentrations for transcripts across the entire sample set. Each line corresponds to a gene and each row to the mean expression value of a replicate. P1 to P7 refer to the number of patterns, followed by the number of genes included in each pattern in parenthesis. A color scale is shown for standardized values in RPKM, per million mapped reads. *n* = 3. Dpp—days post-partum; HCPC—hierarchical clustering on principal components; RPKM—reads per kilobase of transcript.

A PCA (Principal Component Analysis) revealed the biological relevance of the resulting transcriptomic dataset: indeed, the first component (or dimension), containing 84.8% of variances, appeared strongly correlated with the tissue maturity of samples (Figure 3a). Indeed, in vivo samples at each developmental stage (24.5, 30.5, 36.5 and 53.5 dpp) clustered together, whereas immature 14.5 dpp tissues clustered with all the in vitro-cultured tissues (D6, D16, D22, D28). A more precise analysis showed that, in this last cluster, 14.5 dpp tissues clustered away from cultured explants (Dimension 1, 52.4% of variance), demonstrating a persistent difference between in vivo- and in vitro-matured testicular tissues (Figure 3b). The analysis of DEGs confirmed the differences between in vivo and in vitro conditions from the outset of the first wave of spermatogenesis (Figure S2).

Based on the above PCA and on histological observations, we compared the transcriptomes of cultured tissues to those of 14.5 dpp tissues to highlight the genes involved in the initiation of germ cell maturation which could be deregulated during cultures, thereby leading to premature spermatogenic arrest (Figure 3c). By applying stringent selection parameters, we were able to highlight 1240 DEGs (differentially expressed genes), among which 600 were deregulated in each comparison (Figure 3c, Table S1). A clustering analysis on the resulting set of genes allowed us to generate seven expression patterns (termed P1–7), which can be organized into two broad clusters, P1–P4 and P5–P7, regrouped due to their extremely broad expression profiles (Figure 3d, Table S1): cluster P1–4 corresponds to 215 genes transiently expressed in the testis during its maturation and predominantly down-regulated in cultured tissues, and cluster P5–7 corresponds to 385 genes poorly expressed during the maturation process but up-regulated in cultured tissues (Figure 3d, Table S1).

### 2.3. The Functional Analysis Revealed Associations with Specific Biological Processes

**Figure 4:**
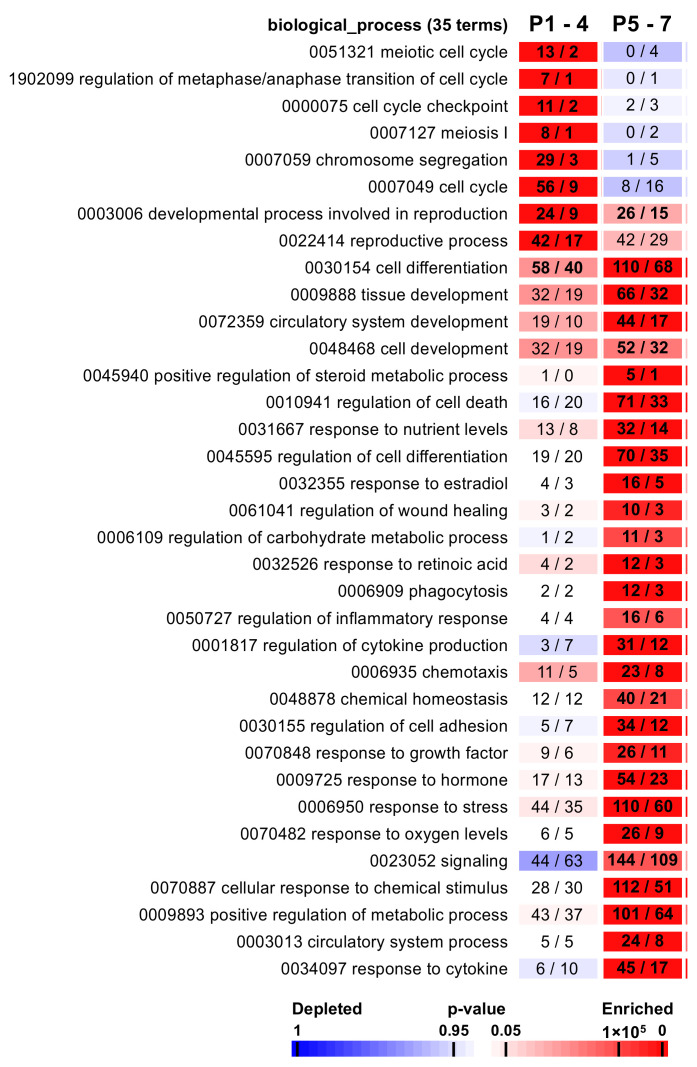
Gene ontology enrichment in cultured testicular tissues. Each cluster is matched with a selection of enriched GO terms from the ontologies “biological process” and “cellular component”. Significantly enriched “biological process” terms and their identification numbers are given, followed by the total number of genes associated with the term and the numbers of genes observed vs. expected by chance. Significant GO terms for each pattern were highlighted with bold values. A color code indicates overrepresentation (red) and underrepresentation (blue) as indicated in the *p* value scale bar.

In order to investigate the molecular pathways involved in this defective maturation process, we performed a functional analysis, based on a GO (gene ontology) enrichment, on the seven resulting expression patterns. This functional analysis highlighted 1056 enriched GO terms (Table S2) significantly associated with the P1–4 and P5–7 patterns, among which 35 biological processes have been selected for representation (Figure 4). Out of the 215 genes belonging to the P1–4 pattern, 42 are involved in reproductive processes (*p* value < 4.58 × 10^6^), 56 in the cell cycle (4.13 × 10^25^), and 58 in cell differentiation (0.037) (Figure 4). The P1–4 pattern was also found to be enriched in genes involved in chromosome segregation (number of genes = 29, *p* value < 9.23 × 10^18^) and cell cycle checkpoints (11, 0.0002), as well as in genes involved in metaphase/anaphase transition (7, 0.0002) (Figure 4), reflecting the clear defect in synaptonemal complex organization. The 385 genes downregulated in in vivo controls and gradually upregulated during the culture process (pattern P5–7) (Figure 4) appeared to be mainly involved in general mechanisms such as tissue development (66, 7.97 × 10^7^), cell development (52, 0.007), cell death (71, 6.24 × 10^8^), stress (110, 4.52 × 10^9^), or inflammation (16, 0.007) (Figure 4). Interestingly, more specific terms which may influence spermatogenesis are also enriched. Indeed, due to the culture condition, explants were exposed to a more harmful environment than under physiological conditions, with direct exposure to oxygen, and thus to oxidative stress. This was reflected by a significant association with the oxygen level GO term (26, 3.30 × 10^5^) (Figure 4). Additionally, genes whose expression could be affected by the culture medium and its supplementation were enriched for gene sets involved in nutrient level (32, 0.0003) and response to hormonal stimulation (54, 2.10 × 10^7^), as well as growth factors (26, 0.0008) (Figure 4). A KEGG (Kyoto Encyclopedia of Genes and Genomes) pathway analysis confirmed the involvement of the cell cycle (11, 1.98 × 10^5^), but also of the reproductive (oocyte meiosis—8, 0.003; progesterone-mediated oocyte maturation—7, 0.004) and immune system (TNF signaling—11, 7.17 × 10^4^; cytokine–cytokine receptor interaction—16, 0.001) signaling pathways (Table S2).

By exploring these gene sets with a manual bibliography, we identified 102 genes clearly related to spermatogenesis and/or SSC maintenance. Among them, 51 belong to the pattern P1–4 and 51 to the pattern P5–7 (Table 1, Table S3). The top 15 most differentially expressed genes (according to their adjusted F-values) associated with spermatogenesis were *Cyp17a1*, *Insl3*, *Tesk1*, *Cdc34*, *Kif11*, *Ube2c*, *Incenp*, *Plk1*, *Ccnf*, *Cks2*, *Alox15*, *Bub1b*, *Ccna2*, *Cbx2*, and *Aspm* for the pattern P1–4 (Table 1), and *Mmp9*, *Vldlr*, *Nr1d1*, *Igfbp3*, *Cxcl10*, *Abca1*, *Fstl3*, *Wnt4*, *Cd14*, *Ctsh*, *Stat3*, *Cx3cl1*, *Kdr*, *Srebf1*, and *Tf* for the pattern P5–7 (Table 1). At least 43 out of the 102 genes related to gametogenesis were expressed in spermatocytes, which included 6 genes (*Cbx2*, *Sohlh2*, *Tex12*, *Trip13*, *Plk1*, and *Plk2*) related to the synaptonemal complex. Most of them were down-regulated in cultured tissues, and may be related to the synapsis defect observed in vitro (Table 1, Table S3). Moreover, genes involved in DSBs and crossover formation (*Top2a*, *Eme1*, *Trip13*, and *Bard1*) were down-regulated (Table 1, Table S3). These gene expression patterns were consistent with our previous observations. In addition, numerous genes related to inflammation and stress response pathways were deregulated, such as *Cxcl10*, *Cd14*, *Cx3cl1*, *Alox15*, *Ccl2, Ccl5*, *Socs3*, and *Fcgr3a* (Table 1, Table S3). The immunological response of the testicular tissue is extremely important for its protection but, in some cases, could induce immunological infertility [53,54]. The recruitment of immune cells by the interstitium appeared to be extremely stimulated under our culture conditions (Table 1, Table S3). Additionally, some genes involved in tissue growth and tissue homeostasis were upregulated (*Srebf1*, *Slc2a1*, *Igf2*, and *Igfbp3*), reflecting the beginning of testicular maturation (Table 1, Table S3). An overexpression of genes involved in the reorganization of the BTB junctions (*Icam1*, *Ctsl*, *Mmp9*, *Mmp2*, and *Spp1*), which are essential for spermatogenic differentiation [55], was also noticed (Table 1, Table S3). Additionally, we observed a significant down-regulation of transcripts encoding key steroidogenic enzymes (*Cyp17a1*, *Cyp11a1*, and *Hsd3b1*) in cultured tissues (Table 1, Table S3). Other genes involved in the regulation of steroidogenesis *(Shbg*, *Pdpn*, *Lpar1*, *Apoe*, *Adrb1*, *Igfbp3*, *Adm*, *Hsd11b1*, *Ngfr*, and *Nr1d1*) were up-regulated (Table 1, Table S3). Finally, at least 52 genes out of 102 are involved in cell cycle processes, such as SSC self-renewal (*Ccna2*, *Igf2*, *Epha2*), spindle organization and chromosomal segregation *(Kif11*, *Aspm*, *Cdca2*, *Haus7*), cell cycle checkpoints (*Ube2c*, *Incenp*, *Plk1*, *Bub1b*), or meiotic entry (*Vldlr*, *Nr1d1*, *Rbp1*) (Table 1, Table S3).

The expression pattern of selected spermatogenesis-related genes was confirmed for the most part in 14.5 dpp testes and cultured explants by RT-qPCR. Indeed, our data showed a significant decrease in *Cbx2*, *Cks2*, *Top2a*, and *Ube2c* mRNA levels during the culture period (Figure S3). As is consistent with RNA-seq data, similar *Dlk1* mRNA levels were found in 14.5 dpp and in vitro cultured tissues, whereas *Ngfr* mRNA levels were deregulated in organotypic cultures (Figure S3). However, differences in *Bub1b* and *Sohlh2* mRNA levels were observed between RNA-seq and RT-qPCR analyses (Figure S3).

### 2.4. Impaired BTB Formation Could Impede Meiotic Progression

**Figure 5:**
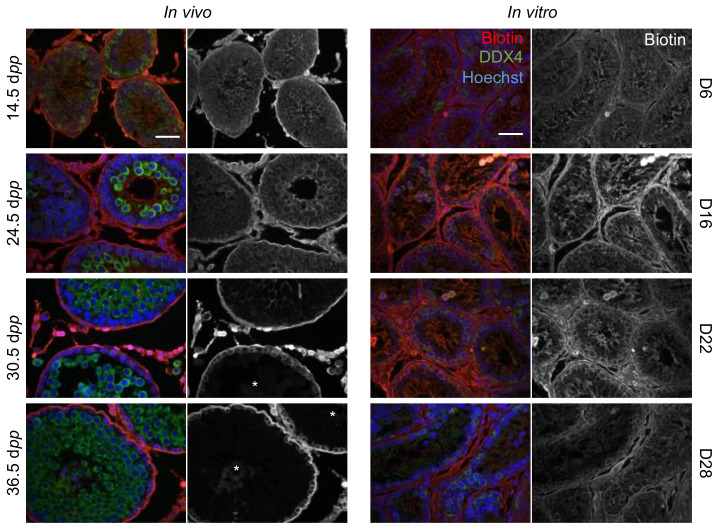
Analysis of the establishment and integrity of the blood–testis barrier throughout in vitro culture. The biotin tracer was revealed in red with Alexa fluor^®^ 594-conjugated streptavidin. A staining with a DDX4 antibody in green was performed to detect germ cells. Nuclei were counterstained with Hoechst. The in vivo kinetics showed a progressive establishment of the BTB. The absence of a functional BTB was highlighted by the presence of the biotin tracer within the adluminal compartment of seminiferous tubules at 14.5 and 24.5 dpp. Tubules with an in-tact BTB (annotated with a star) were observed at 30.5 dpp and 36.5 dpp: in these tubules, the biotin tracer was restricted to the basal compartment. In in vitro cultured tissues, biotin permeated throughout the seminiferous epithelium in all the tubules observed, showing an impairment in the establishment of the BTB. Scale bar: 50 µm; Magnification: ×400. BTB—blood–testis barrier; dpp—days post-partum.

We then chose to investigate BTB formation and steroidogenesis during organotypic cultures, since several genes involved in these biological processes were deregulated in vitro. To assess the establishment and integrity of the BTB in cultured tissues, testicular fragments were incubated with a biotin tracer supposed to be stopped at the level of the BTB and not to diffuse within the adluminal compartment of seminiferous tubules. Our kinetic analyses show the presence of this tracer throughout the seminiferous epithelium in all the tubules examined, thereby showing an impairment of BTB establishment that could impede meiosis (Figure 5). On the contrary, in cultures of mouse prepubertal testicular tissues [153], the same analyses revealed a good preservation of BTB integrity.

**Figure 6:**
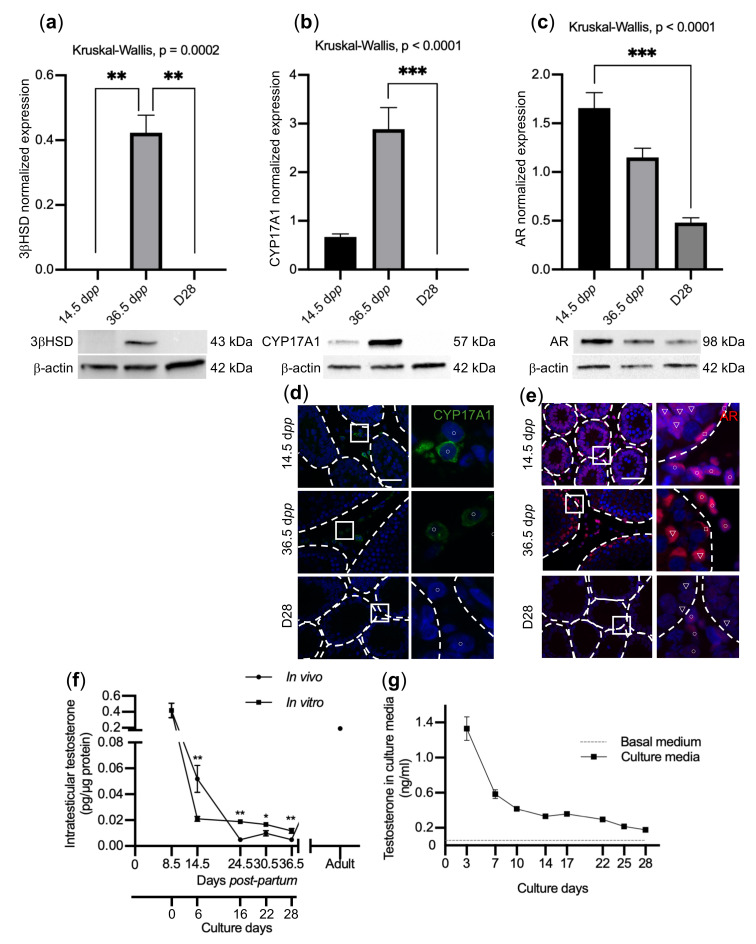
Analysis of steroidogenesis in in vitro culture. Expression of 3βHSD (**a**), CYP17A1 (**b**), and AR (**c**) was quantified by western blot. Tissues cultured for 28 days (D28), and tissues recovered from age-matched in vivo controls (36.5 dpp) and from immature in vivo controls (14.5 dpp) were analysed. Representative data are presented. *n* = 6. (**d**) CYP17A1 immunostaining in Leydig cells (circle). (**e**) AR immunostaining in Sertoli cells (triangle), peritubular cells (square), and Leydig cells (circle). Seminiferous tubules are delimited by dotted lines, and the panels on the right represent enlarged views of the boxes. Scale bar: 50 µm; Magnification: ×400. Intratesticular testosterone levels (**f**) and levels of testosterone in culture media (**g**). For statistical analyses, Kruskal–Wallis tests followed with Dunn’s post-test were applied for (**a**–**c**), and Mann–Whitney tests were applied for (**f**,**g**). Data are expressed as mean ± s.e.m. * *p* value < 0.05; ** *p* value < 0.01; *** *p* value < 0.001.

Steroidogenesis was then examined by first analysing the expression of both 3βHSD and CYP17A1 by western blot and immunofluorescence. Interestingly, the expression of these two steroidogenic enzymes was undetectable at D28, contrary to the age-matched in vivo control (Figure 6a,b,d). Testosterone levels were measured within cultured testicular fragments and culture media using LC-MS/MS. They first dropped and then slowly decreased during the culture (Figure 6f,g). The levels found in testicular fragments between D16 and D28 are slightly above the physiological levels (Figure 6f). However, a weak expression of the androgen receptor (AR) was observed at D28 (Figure 6c,e). One can therefore speculate that the decrease in AR, which mediates the action of testosterone, might affect BTB formation during the first wave of in vitro spermatogenesis [154].

### 2.5. The In Vitro Spermatogenesis Process May Be Associated with an Inflammatory Process

**Figure 7:**
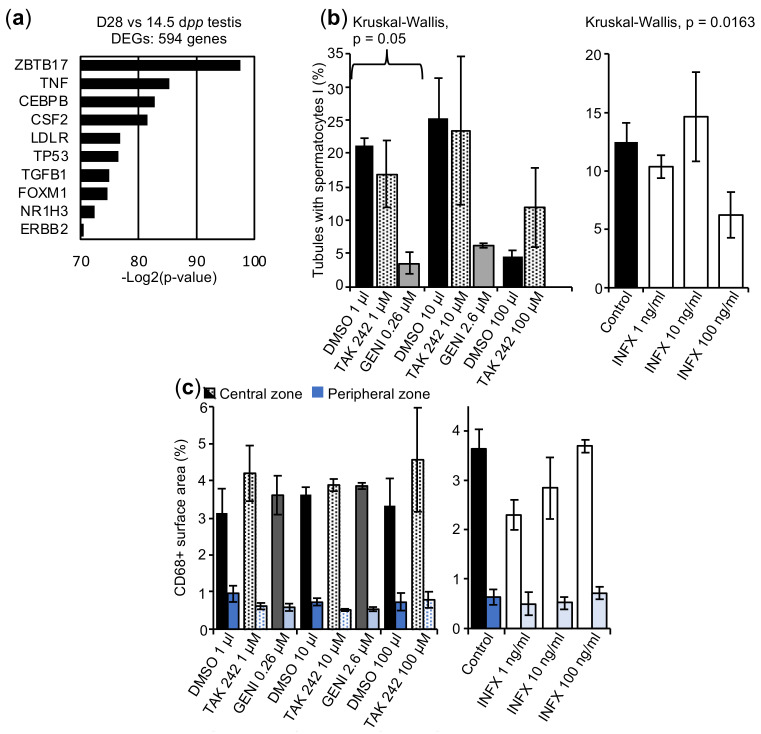
Analysis of in vitro cultures supplemented with anti-inflammatory molecules. (**a**) Predicted upstream transcriptional regulators of 594 DEGs between D28 in vitro cultured tissues and 14.5 dpp in vivo controls. (**b**) Percentage of seminiferous tubules at the spermatocyte I stage after 28-day cultures with anti-inflammatory molecules. (**c**) Percentage of surface area occupied by CD68+ macrophages. Left and right bars refer to the percentage of CD68+ surface area within the central and peripheral zones of the testicular fragments, respectively. *n* = 3. For statistical analyses, Kruskal–Wallis tests followed with Dunn’s post-test were applied. Tissues cultured with TAK 242 or Genistein (GENI) were compared to tissues cultured with DMSO. Tissues cultured with Infliximab (INFX) were compared to tissues cultured without DMSO. Data are expressed as mean ± s.e.m.

To identify a potential common regulator of these deregulated genes, the upstream regulator tool from Ingenuity Pathway Analyses (IPA) software was used. IPA analyses clearly demonstrated that zinc finger and BTB domains containing 17 (ZBTB17), tumor necrosis factor (TNF), and CCAAT/enhancer binding protein beta (CEBPB) were the top three predicted upstream regulators of the 594 deregulated genes between 14.5 dpp and D28 cultured tissues, among the 600 candidate genes (Figure 7a, Table S4). Most of the top 10 predicted upstream regulators were involved in inflammatory processes or immune cell recruitment. We hypothesized that the maturation arrest observed in vitro could be associated with a strong response of the local inflammatory system, probably generated by the resident macrophages, as previously described in cultures of mouse prepubertal testicular tissues [49]. To test this hypothesis, tissue cultures were supplemented for 28 days with anti-inflammatory molecules. The analysis of the progression of spermatogenesis shows no germ cell differentiation beyond the spermatocyte I stage, whatever the molecule added into the culture medium (Figure 7b). Moreover, none of the molecules tested were efficient in significantly increasing the percentage of seminiferous tubules containing spermatocytes I, especially genistein, which not only alters meiotic progression but also tissue architecture at the highest concentration used (Figure 7b). The molecules tested also failed to decrease the macrophage population, since the CD68+ surface area was not significantly different between the conditions (Figure 7c).

## 3. Discussion

The current study explored the in vitro maturation of rat prepubertal testicular tissues at the molecular level in order to unravel the mechanisms underlying meiotic arrest. Indeed, although organotypic culture at a gas–liquid interphase has proven to be a promising approach for generating spermatozoa from immature testicular tissues in the mouse model, a complete in vitro spermatogenesis has not yet been achieved in other species, including rats and humans, thereby delaying its application in clinics for fertility restoration of survivors of childhood cancer.

Consistently with our previous findings [16], no germ cells beyond the spermatocyte I stage were observed in rat organotypic cultures. In vitro cultured tissues were analysed at D28 (corresponding in vivo to 36.5 dpp), at which time round spermatids should be physiologically detected in seminiferous tubules. The proliferative activity of intratubular cells was maintained under the culture conditions. However, because of the meiotic blockage, a significant decrease in germ cell content was found within seminiferous tubules compared to age-matched in vivo controls. Although more tubules showed DNA fragmentation when comparing in vitro to in vivo conditions, few pachytene spermatocytes were TUNEL positive, suggesting that apoptosis occurred after this stage. Two previous studies have reported that round spermatids could be obtained after in vitro maturation of rat prepubertal testes, albeit in very small proportions [14,15]. 

Two distinct types of meiotic arrest have been described in humans [155]: (i) arrested spermatocytes are characterized by the absence of a discernible XY body and a dispersed γH2AX nuclear staining, or (ii) arrested spermatocytes display normal features, i.e., the presence of an XY body displaying γH2AX staining. Here, the arrested rat spermatocytes displayed a γH2AX-positive XY body and numerous γH2AX-positive foci along the chromosomes, reflecting an increase in DSBs and/or asynapsis. Moreover, the cytological analyses of the rat spermatocytes generated in vitro revealed a disorganization of synaptonemal complexes and asynapsis, which is known to induce a defect in the recruitment of ATR and BRCA1, thereby leading to a defective inactivation of XY chromosomes, and to apoptosis [34]. It would be interesting to explore the expression of key proteins involved in DSBs formation and repair processes, such as RAD51, BRCA1, and DMC1, to characterize more precisely the maturation arrest phenotype.

Meiotic arrest can have a genetic [156,157] or an environmental [158,159] origin. In order to overcome maturation arrest, it is essential to understand the molecular mechanisms that are deregulated during in vitro culture. Interestingly, our bulk RNA-Seq analyses revealed that D16, D22, and D28 cultured tissues showed immature features, with transcriptomic profiles close to 14.5 dpp in vivo controls. A total of 600 DEGs were identified between in vitro- and in vivo-matured testicular tissues. Using gene ontology analyses, different GO terms in relation with spermatogenesis, BTB, steroidogenesis, and synaptonemal complex were highlighted. The assessment of BTB functionality revealed its permeability in rat organotypic cultures, contrary to what was observed in cultured prepubertal mouse explants [153]. The analysis of proteins involved in BTB structure (i.e., OCLN, ZO-1) and regulation (i.e., SRC, FAK) will have to be investigated in future studies. Since BTB’s primary function is to maintain an immunological sanctuary inside the seminiferous tubules, its dysfunction has been shown to cause immunological damage, impede meiosis [55,160], and contribute to infertility [54]. The assessment of steroidogenesis first unveiled the absence of expression of the two steroidogenic enzymes 3βHSD and CYP17A1 at D28. The slightly elevated intratesticular levels observed between D16 and D28, which could arise from the lack of a functional vascular system in vitro, is unlikely to be the cause of the meiotic arrest. However, the decreased expression of AR, which mediates testosterone signaling, could explain this meiotic blockage. Indeed, impaired androgen signaling has previously been shown to disrupt BTB formation and integrity [154,161,162], to interfere with the assembly of the synaptonemal complex [163], and to induce meiotic arrest [46,47,164].

Then, IPA analyses performed on our dataset led to the identification of predictive upstream regulators. We found that the top 10 upstream regulators were involved in inflammation and in regulation of spermatogenesis. Among them, TNFα is partly produced by macrophages to promote the inflammatory process, and its expression is accompanied by the production of reactive oxygen species (ROS) or chemokines [165]. In addition, TNFα has been shown to be activated by the NF-κB signaling pathway, which contributes to immune cell survival and proliferation, followed by the production of various cytokines [166]. These results are consistent with a recent transcriptomic analysis that revealed the presence of a drastic and overwhelming immune reaction involving the TLR4-NF-κB signaling pathway in cultures of prepubertal mouse testicular tissues [49]. Moreover, we have previously shown that cytoplasmic and nuclear ROS are generated in in vitro cultured mouse testes [167]. Cytokines such as TNFα have also been shown to regulate BTB permeability by restructuring gap junctions [168,169,170], as well as the steroidogenic function of Leydig cells [171,172,173]. As mentioned earlier, an altered BTB integrity, a downregulation of steroidogenic genes (*Cyp11a1*, *Cyp17a1*, *Hsd3b1*), and a depletion in CYP17A1 and 3βHSD were evidenced in organotypic cultures in the current study. We therefore assessed the effect of three molecules with anti-inflammatory properties (TAK 242, infliximab, genistein) on spermatogenic progression in organotypic cultures. Among them, TAK 242, a TLR4-NF-κB signaling pathway inhibitor, has previously been used to successfully decrease the number of testicular macrophages in cultures of mouse prepubertal testicular tissues [49]. Interestingly, our IPA analysis predicted at least six regulators (ZBTB17, TNF, CEBPB, TP53, FOXM1, and NR1H3) that interact with the TNFα-NF-κB signaling pathway [174,175,176,177,178,179]. However, the three molecules tested, including TAK 242, failed to promote meiotic progression and to reduce the macrophage population in cultures of rat tissues, at all concentrations tested. By testing molecules that inhibit other inflammatory pathways, the in vitro meiotic arrest may be unlocked in the future. In addition, the use of a single-cell RNA-Seq approach will be useful in identifying DEGs in each testicular cell population, and therefore better decipher the molecular mechanisms leading to maturation arrest in spermatocytes during organotypic culture.

In conclusion, through a combination of histological and molecular analyses, we uncovered for the first time several underlying molecular mechanisms that could explain the meiotic arrest observed in cultures of rat prepubertal testicular tissues. Our data converge towards an alteration in BTB integrity, in the steroidogenic pathway, and possibly an inflammatory response. The utilization of the transcriptomic data obtained in this study will be useful to further optimize in vitro tissue maturation protocol, particularly the crucial meiotic step, before considering a clinical application

## 4. Materials and Methods

### 4.1. Animal Care and Handling

Rats were housed in a temperature- and humidity-controlled environment, under a 12h light/dark cycle. Food and water were provided ad libitum. Pubertal and adult rats were euthanized by CO_2_ asphyxiation, whereas pups aged 8.5 dpp were decapitated. In this study, 106 rats were used.

### 4.2. Animals and Tissue Samples

Wistar rats (*Rattus norvegicus*) were purchased from Charles River Laboratories (L’Arbresle, France) and bred in our animal facility. After euthanasia, testes were biopsied and rinsed in αMEM (Gibco Life Technologies, Saint-Aubin, France) at 4 °C. The tunica albuginea was removed with two needles under a binocular magnifier (S8AP0, Leica Microsystems, Wetzlar, Germany). 

For organotypic cultures, testicular tissues were collected at 8.5 dpp and prepared as described below. For histology and immunohistochemistry, tissues from in vivo controls or from in vitro cultures were fixed at room temperature in Bouin’s fixative solution (Sigma-Aldrich, Saint-Quentin Fallavier, France) or 4% paraformaldehyde (PFA, Sigma-Aldrich, Saint-Quentin Fallavier, France). They were then dehydrated in successive ethanol/xylene baths, embedded in paraffin and cut into thin sections (3 µm thick). After two xylene baths, tissue sections were rehydrated in successive descending ethanol baths and plunged into PBS buffer. For immunocytological analyses, cells from in vivo controls or cultured explants were spread onto glass slides. For molecular analyses, the central necrotic area in cultured explants was removed with two thin needles, before being stored in liquid nitrogen.

### 4.3. Organotypic Cultures

Testicular explants were cultured using the gas–liquid interphase method as described before [16]. Briefly, 8.5 dpp testes were cut into 6 fragments and cultured onto agarose gels (Sigma-Aldrich, Saint-Quentin Fallavier, France) in 6-well plates (ThermoFisher Scientific, Saint-Aubin, France). The day before the beginning of each culture, agarose gels were soaked completely in basal medium (αMEM + 0.01% gentamicin) and kept overnight in the incubator [6,16]. Tissues were maintained under 5% CO_2_–95% air at 34 °C for 6, 16, 22, or 28 days. The culture medium was composed of αMEM supplemented with 10% Knock-out serum replacement (Gibco Life Technologies, Saint-Aubin, France), 10^−6^ M retinol (Sigma-Aldrich, Saint-Quentin Fallavier, France) and 3.4 mM vitamin E (Sigma-Aldrich, Saint-Quentin Fallavier, France). To assess the effect of anti-inflammatory molecules, organotypic cultures were supplemented for 28 days with either TAK 242 (1, 10, or 100 µM in DMSO; Sigma-Aldrich, Saint-Quentin Fallavier, France), Infliximab (1, 10, or 1000 ng/mL in medium; Sigma-Aldrich, Saint-Quentin Fallavier, France) or genistein (0.26, 2.6, and 26 µM in DMSO; Merck-Millipore, Molsheim, France). Cultures supplemented with TAK 242 or genistein were compared to cultures supplemented with DMSO (1, 10 or 100 µL). The media were prepared and replaced every 3–4 days. Juvenile male rats at 14.5, 24.5, 30.5, and 36.5 dpp were used as in vivo controls.

### 4.4. Histological and Immunohistochemical Analyses

All the antibodies used in this study for immunohistochemical analyses are presented in Table S5. Unless stated otherwise, only the healthy peripheral zone of the cultured testicular explants was taken into consideration for the histological and immunohistochemical analyses.

#### 4.4.1. HES

Hemalun–Eosin–Saffron staining was performed to identify the different meiotic germ cells and division figures [180]. 

Ki67 and DDX4: To assess cell proliferation (Ki67) and germ cell (DDX4)/Sertoli cell ratio, tissues sections were subjected to a standard immunofluorescence protocol as described below. Tissue sections fixed in PFA were incubated in citrate buffer (Diapath, Martinengo, Italy) at 96 °C for 40 min and cooled for 20 min at RT. After rinsing in distilled water, sections were permeabilized in PBS-Triton X-100 0.01% for 15 min at RT (only for Ki67). To avoid nonspecific staining, sections were blocked with 5% BSA + 5% horse serum (Ki67) or with 20% goat serum + 5% BSA (DDX4). Slides were then incubated overnight with primary antibodies at 4 °C. Negative controls were performed with pre-immune IgGs. After 3 washes in PBST, sections were incubated with secondary antibodies coupled to Alexa Fluor^®^ 488 for 1 h at RT. Sections were rinsed, dehydrated with ethanol, and mounted in Vectashield (Vector, Eurobio, Les Ulis, France) with Hoechst 33342 (ThermoFisher Scientific, Saint-Aubin, France).

#### 4.4.2. TUNEL-SYCP3

To detect DNA fragmentation and spermatocytes in the same samples, tissue sections were first subjected to TUNEL assays (In Situ Cell Death kit POD, Roche, Mannheim, Germany), following the manufacturer’s instructions. Then, sections were post-fixed with 4% PFA for 15 min at RT and finally underwent the standard immunofluorescence protocol described above (without the permeabilization step). Rabbit anti-SYCP3 antibodies were used, and revealed with secondary antibodies coupled to Alexa Fluor^®^ 594. Positive controls were performed with DNase I before TUNEL assays and negative controls were carried out by omitting the enzyme solution (TUNEL) or with pre-immune IgGs (SYCP3 immunostaining).

#### 4.4.3. CD68

Tissue sections fixed with PFA were submitted to a standard immunofluorescence protocol, as previously described. CD68-immunolabeled testicular tissue images were acquired on a Thunder upright microscope (Leica Microsystems, Wetzlar, Germany). The surface area occupied by CD68+ macrophages was measured both in the peripheral (healthy) and central (necrotic) regions of the cultured explants.

#### 4.4.4. γH2AX

Tissue sections fixed with Bouin’s solution were incubated in citrate buffer at 96 °C for 10 min for antigen retrieval, and cooled down for 5 min at RT. After rinsing with distilled water, endogenous peroxidases were blocked with HP block (Dako, Les Ulis, France) for 15 min at RT. After 3 washes in PBST, nonspecific binding sites were then blocked for 30 min at RT with 20% goat serum, 5% BSA in PBS-Tween 0.05%. Tissue sections were incubated for 2 h at RT with anti-γH2AX antibodies diluted in blocking buffer. Subsequently, primary antibodies were detected with polyvalent secondary antibodies for 5 min at RT. Staining was achieved after incubation with a streptavidin-horseradish peroxidase solution (ThermoFisher Scientific, Saint-Aubin, France) for 15 min at RT followed by 3,3′-diaminobenzidine substrate for 30 s at RT. After nuclear counterstaining with hematoxylin (Diapath, Martinengo, Italy), sections were dehydrated in successive ethanol baths of increasing concentrations, followed by two xylene baths and mounting with Eukitt (Sigma-Aldrich, Saint-Quentin Fallavier, France).

Each in vivo replicate originated from different rats. For quantitative histological analyses, at least 2 levels of the testis/explant (separated by at least 30 µm) and 30 seminiferous tubules were analysed for each replicate. For each in vitro replicate, 2–3 testicular explants were analysed. In total, approximately 360 tubules were counted for in vivo controls and at least 720 tubules for the in vitro condition, per analysis. 

HES and γH2AX immunostaining were analysed with a DM4000B light microscope (Leica Microsystems, Wetzlar, Germany) equipped with Leica Application Suite software (LAS; Leica Microsystems, Wetzlar, Germany). Immunofluorescence analyses (except for CD68) were performed on an epifluorescence microscope (DMRBE, Leica Microsystems, Wetzlar, Germany GmbH) equipped with a monochrome CCD IEEE1394 FireWire video camera (Perceptive Instruments, Bury St Edmunds, UK).

### 4.5. Chromosome Spreads

Preparations of meiotic chromosome spreads from rat spermatocytes were adapted from previously published protocols [181,182] with slight modifications. Briefly, testicular tissue fragments from in vivo controls and in vitro cultured testicular explants were placed into a hypotonic extraction buffer (30 mM Tris, 50 mM sucrose, 17 mM sodium citrate, 5 mM ethylenediaminetetraacetic acid, 0.5 mM dithiothreitol, 0.5 mM phenylmethanesulfonyl fluoride, 118 mM potassium chloride, pH 8.2) for 40 to 60 min at RT. Fragments were then placed on a 20 µL drop of 100 mM sucrose, and the central necrotic area from in vitro cultured testicular explants was removed (2–3 explants per drop). Tissues were mechanically dispersed with two needles to obtain a cell suspension. After adding a 20 µL drop of sucrose solution and removing the remnant seminiferous tubules, cells were gently resuspended and spread (according to the drying-down technique) onto two Superfrost Plus glass slides previously coated with 1% PFA + 0.15% Triton X-100. Nuclei were air dried for at least 2 h in a humidified chamber. Finally, preparations were washed twice for 2 min in freshly prepared 0.4% Photoflo (Kodak, Rochester, NY, USA) and dried at RT. Preparations were stored at −20 °C and used within 2 weeks.

### 4.6. Analysis of Chromosome Synapsis

After 2 min at 37 °C, chromosome spreads were rehydrated in PBST for SYCP3/SYCP1 immunostaining or in successive Triton X-100 baths (5 min in PBS + 0.5% Triton X-100, 5 min in PBS + 0.05% Triton X-100) for SYCP3/γH2AX immunostaining. Spreads were blocked for 30 min at RT with 5% BSA + 5% horse serum in PBS (SYCP3/SYCP1) or with 2% BSA + 0.05% Triton X-100 in PBS (SYCP3/γH2AX). They were then incubated overnight with primary antibodies at 4 °C (SYCP3/SYCP1) or RT (SYCP3/γH2AX) in a humidified chamber. Negative controls were performed with pre-immune antibodies. After 3 washes, preparations were incubated with appropriate secondary antibodies. Biotin was revealed with Alexa Fluor^®^ 594 coupled to streptavidin for 30 min at RT. Preparations were rinsed, dehydrated with ethanol, and mounted in Vectashield (Vector, Eurobio, Les Ulis, France). The slides were sealed with polish for confocal microscopy. For the analysis of chromosome synapsis, spermatocytes were isolated from at least three 36.5 dpp control rat testes and D28 in vitro cultured tissues.

### 4.7. Classification of Prophase I Stages

The classification was performed as previously described [183]. Briefly, spermatocyte stages were identified by the morphology of the lateral elements (LE) revealed by SYCP3 immunostaining. At the early leptotene stage, LE appear as thin, dot-like fragments. At the leptotene stage, they are numerous and appear as short fragments. At the zygotene stage, LE elongate to form filaments. At the early zygotene stage, synapsis is not apparent or only in few and short fragments. The mid-zygotene stage is characterized by synapsis extension. At the late zygotene stage, LE are shorter than in mid-zygotene and almost all the chromosomes are synapsed extensively with their homologs, only few areas are not yet synapsed. At the pachytene stage, LE are shorter and all the chromosomes are synapsed. At the diplotene stage, synapsed LE begin to be released and chiasmata can be observed.

### 4.8. Confocal Microscopy Analysis

Confocal images (1024 × 1024 pixels) of meiotic chromosome spreads were acquired using an upright fixed-stage Leica TCS SP8 CFS confocal microscope (Leica Microsystems, Wetzlar, Germany) equipped with diode lasers (Coherent, Les Ulis, France) at 488 nm to excite Alexa Fluor^®^ 488 and at 552 nm to excite Alexa Fluor^®^ 594 and a conventional scanner at 400 Hz. Using a 63× (1.40, oil immersion), fluorescence emission was sequentially detected through a hybrid detector (Leica Microsystems, Wetzlar, Germany) in a photon counting mode with a specific band from 500 to 540 nm for Alexa Fluor^®^ 488 and 600 to 650 nm for Alexa Fluor^®^ 594. Z-stack images were acquired using an adapted step-size of 250 nm. 3D images (xyz) reconstruction and analysis were performed using Imaris software (Bitplane, Zurich, Switzerland). ImageJ (Rasband W.S., U.S. National Institutes of Health, Bethesda, MD, USA, http://imagej.nih.gov/ij/, 1997–2020, accessed on 8 September 2020) was used to adjust brightness and contrast for representative image treatment.

### 4.9. RNA Extraction

Total RNA was extracted according to the manufacturer’s instructions using an RNeasy Micro Kit (Qiagen, Hilden, Germany), and stored at −20 °C until use. Genomic DNA was removed by incubation with Turbo DNase (Invitrogen, Villebon-Sur-Yvette, France) and RNA quality control was performed before RNA sequencing and RT-qPCR on Agilent Bioanalyzer (Les Ulis, France). All the samples chosen for analysis displayed an RNA integrity number (RIN) value greater than or equal to 8.5.

### 4.10. RNA-Seq Library Preparation and Sequencing

RNA-Seq libraries were generated from 400 ng of total RNA using a TruSeq Stranded mRNA LT Sample Preparation Kit (Illumina, San Diego, CA, USA), according to the manufacturer’s instructions. Briefly, following purification with poly(T)-oligo attached magnetic beads, mRNA was fragmented using divalent cations at 94 °C for 2 min. The cleaved RNA fragments were copied into first strand cDNA using reverse transcriptase and random primers. Strand specificity was achieved by replacing dTTP with dUTP during the second strand cDNA synthesis using DNA polymerase I and RNase H. Following the addition of a single ‘A’ base and subsequent ligation of the adapter on double stranded cDNA fragments, the products were purified and enriched with PCR (30 s at 98 °C; (10 s at 98 °C, 30 s at 60 °C, 30 s at 72 °C) × 12 cycles; 5 min at 72 °C) to create the cDNA library. The excess of PCR primers was further removed by purification using AMPure XP beads (Beckman-Coulter, Villepinte, France), and the final cDNA libraries were checked for quality and quantified using capillary electrophoresis. Libraries were then single-read sequenced with a length of 50 bp on an Illumina Hiseq4000 sequencer. Image analysis and base calling were carried out using RTA v.2.7.7 (Illumina, San Diego, CA, USA) and bcl2fastq v.2.17.1.14 (Illumina, San Diego, CA, USA). 

For each time point, three independent sets of total RNA were isolated and used as a template for RNA-seq library preparation. In addition, for each cultured sample, testicular tissue fragments originated from a minimum of 3 juvenile males. Thus, a minimum of 9 animals per time point was used to minimize/normalize the effect of genetic variability.

### 4.11. RNA-Seq Analysis

#### 4.11.1. Read Mapping

Reads from each individual sample were aligned to the rn6 release of the rat genome with STAR (version 2.5.2a), [184] using previously published approaches [185,186,187,188]. Briefly, the STAR program was first run for each fastq file, using the RefSeq transcript annotation (GTF format) of the rat genome (release rn6) downloaded in November 2017. Exon junction outputs were then added to a splice junction set. STAR was next run a second time, complemented by the splice junction dataset, to produce a final alignment file (BAM format) for each sample.

#### 4.11.2. Transcriptome Quantification

RefSeq transcripts were quantified with StringTie (version 1.3.3), with default settings applied [189]. Transcript abundances were next normalized with Ballgown (available in the StringTie suite), expressed as reads per kilobase of exon model per million reads mapped (RPKM).

#### 4.11.3. Principal Component Analysis

Principal component analysis (PCA) was performed based on the gene abundance values with the FactoMineR package to graphically evaluate the distribution of sequenced samples [190].

#### 4.11.4. Statistical Filtration and Clustering Analysis

The statistical filtration of the genes showing a differential expression (DE) across experimental samples was performed using AMEN [191]. Out of 17,322 genes in the dataset, we first selected 11,701 “detectable” or “expressed” genes, defined as those for which abundance levels exceeded 0.5 RPKM in at least one experimental condition (median value of sample duplicates). The D6 condition was removed due to the differences in cellular composition (Figure S4). Next, we compared 14.5 dpp testicular tissue samples to D16, D22, and D28 cultured tissue conditions and selected 1490 genes yielding at least one-fold change greater than or equal to 2.0 (median values of sample duplicates). A linear models for microarray data (LIMMA) statistical test was used to identify 1240 genes with significant abundance variations across samples (F-value adjusted with the FDR method: *p* ≤ 0.05) (Smyth, 2004). Finally, we intersected the three sets of DEGs to select 600 genes that showed significant differential abundances in all three comparisons. The resulting 600 genes were next clustered into seven expression patterns (P1-P7) with the unsupervised HCPC algorithm, respectively [190]. The resulting patterns were ordered according to peak expression levels in the different cell types.

### 4.12. Functional Analysis

AMEN [191] was used to calculate the Fisher exact probability, and the Gaussian hypergeometric test was used to identify significantly enriched biological processes (Gene ontology) and pathways (KEGG pathway) associated with the seven expression patterns (P1-P7). A specific term was considered enriched in a group of coexpressed genes if the *p* value was <0.01 and the number of genes in this cluster showing this annotation was >5. Upstream regulator analysis was performed using the Ingenuity Pathway Analysis software (Ingenuity, Qiagen, Hilden, Germany).

### 4.13. Validation of the Transcriptome Data by RT-qPCR

To check the accuracy of the transcriptome data, DEGs identified as important for the process of spermatogenesis were selected for RT-qPCR. Each reaction was performed using three technical replicates and three biological replicates for the robustness of our analysis. RNA extraction and integrity analysis were performed as previously described.

#### 4.13.1. Reverse Transcription

cDNA synthesis was performed using qScript™ cDNA SuperMix (Quanta Biosciences by VWR, Fontenay-sous-Bois, France) according to the manufacturer’s instructions using the following program: 5 min at 25 °C, 30 min at 42 °C, and 5 min at 85 °C.

#### 4.13.2. qPCR

cDNA amplifications were carried out in a total volume of 13 μL containing 6 ng of cDNA templates, 6.5 μL of SYBR Green (ThermoFisher Scientific, Saint-Aubin, France), and 300 nM of each primer. Specific primers are listed in Table S6. Samples were dispensed using the Bravo pipetting robot (Agilent Technologies, Les Ulis, France). Reactions were performed in 384-well plates (Life Technologies) in a QuantStudio 12K Flex system. The amplification condition was 20 s at 95 °C followed by 40 cycles (1 s at 95 °C, 20 s at 60 °C) and a final step of denaturation of 15 s at 95 °C, 1 min at 60 °C, and 15 s at 95 °C. Melting curves were also obtained to ensure the specificity of PCR amplifications. The size of the amplicons was verified by agarose gel electrophoresis (E-gel 4%, Life Technologies). The relative expression level of each gene was normalized to two housekeeping genes, as recommended by the MIQE guidelines [192]: *Gapdh* and actin β (*Actb*), which were identified and validated as the most stable and suitable genes for RT-qPCR analysis in rodent testis development [193]. Data were analysed using the 2^−ΔΔCt^ method [194]. The Kruskall–Wallis test was used to determine significant differences.

### 4.14. BTB Integrity Assay

The assay was performed as previously described [153]. Briefly, testicular fragments were immersed in a 10 mg/mL EZ-Link Sulfo-NHS-LC-Biotin solution (21335, ThermoFisher Scientific, Saint-Aubin, France) in PBS containing 1 mM CaCl_2_ for 30 min at room temperature. Incubation of testicular fragments in 1 mM CaCl_2_ in PBS was used as a negative control. Tissues were fixed with PFA, dehydrated, and prepared as previously described. DDX4 immunofluorescence was then performed as described above, with slight modifications. After antigenic sites revelation with citrate buffer, nonspecific binding sites were then blocked for 30 min at RT with 5% horse serum and 5% BSA in PBS-Tween 0.05%. Then, sections were incubated with a primary antibody against DDX4, revealed with a secondary goat anti-rabbit antibody coupled with Alexa Fluor^®^ 488. Alexa Fluor^®^ 594-conjugated streptavidin was added at the same time to reveal biotin localization. Sections were rinsed, dehydrated with ethanol, and mounted in Vectashield with Hoechst 33342.

### 4.15. Hormone Measurement with LC-MS/MS

#### 4.15.1. Testicular Tissue Preparation

Tissues were flattened out in a PBS + protease inhibitors (5892791001, Sigma-Aldrich, Saint-Quentin Fallavier, France) buffer. Samples were centrifuged for 10 min at 12,000× *g*, 4 °C. The supernatants were collected and stored at −20 °C until use. 

#### 4.15.2. Sample Preparation

The standards for testosterone and stable labelled isotopes were obtained from Merck Millipore. Working solutions were prepared in methanol. Serial dilutions from working solutions were used to prepare seven-point calibration curves and 3 quality control levels for all analytes. For the final calibration and quality control solutions, PBS was used for testicular fragments and αMEM for culture media. The linearity ranges were from 0.05 to 10 ng/mL.

A simple deproteinization was carried out by an automated sample preparation system, the CLAM-2030 (Shimadzu Corporation, Marne-la-Vallée, France) coupled with a 2D-UHPLC-MS/MS system. Once the sample was on board, 30 µL was automatically pipetted in a pre-conditioned tube containing a filter, in which reagents were added, then mixed and filtered. Briefly, the PTFE (polytetrafluoroethylene) filter vial (0.45 μm pore size) was previously conditioned with 20 μL of methanol (Carlo Erba, Val-de-Reuil, France). Successively, 30 μL of sample and 60 μL of a mixture of isotopically labelled internal standards in acetonitrile were added. The mixture was agitated for 120 s (1900 rpm), then filtered, by application of vacuum pressure (−60 to −65 kPa) for 120 s, into a collection vial. Finally, 30 µL of the extract was injected in the 2D-UHPLC-MS/MS system.

#### 4.15.3. 2D-UHPLC-MS/MS Conditions

Analysis was performed on a two dimensional ultra-performance liquid chromatograph-tandem mass spectrometer (2D-UHPLC–MS/MS) consisting of the following Shimadzu^®^ modules (Shimadzu Corporation): an isocratic pump LC20AD SP, for pre-treatment mode; a binary pump consisting of coupling two isocratic pumps Nexera LC30AD for the analytical mode; an automated sampler SIL-30AC; a column oven CTO-20AC; and a triple-quadrupole mass spectrometer LCMS-8060.

The assay was broken down into two stages. The first was the pre-treatment, where the sample was loaded on the perfusion column. The second step was the elution of the compounds of interest to the analytical column.

The deproteinized extract performed by the CLAM-2030 was automatically transferred to the automated sampler, where 30 µL was directly analysed in the chromatographic system.

The LC-integrated online sample clean-up was performed using a perfusion column Shimadzu^®^ MAYI-ODS (5 mm L × 2 mm I.D.). The first step consisted of loading the extract on the perfusion column with a mobile phase composed of 10 mM ammonium formate in water (Carlo Erba, Val-de-Reuil, France) at flow rate of 0.5 mL/minute over 2 min. Then, the system switched to the analytical step to elute the analytes from the perfusion column to the analytical column, to achieve chromatographic separation. During this step, the loading line was washed with propan-2-ol (Carlo Erba, Val-de-Reuil, France) over 3 min. Chromatographic separation was achieved on a Restek^®^ Raptor Biphenyl (50 mm L × 3 mm I.D., 2.7 μm) maintained at 40 °C and a gradient of (A) 1mM ammonium fluoride buffer in water (Carlo Erba, Val-de-Reuil, France) and (B) methanol (Carlo Erba, Val-de-Reuil, France) at flow rate of 0.675 mL/minute as follows: 0.0–2.10 min, 5% (B); 2.1–3.0 min, 5 to 65% (B); 3.0–4.75 min, 65% (B); 4.75–5.0 min, 65 to 70% (B); 5.0–6.6 min, 70% (B); 6.6–8.0 min, 70 to 75% (B); 8.0–8.5 min, 75 to 100% (B); 8.50–9.5 min, 100% (B); 9.5–9.6 min, 100% to 5% (B); 9.6–12.0 min, 5% (B).

Detection and quantification were performed by scheduled-MRM (multiple reaction monitoring) using a 1 millisecond pause time and a 50 millisecond dwell time to achieve sufficient points per peak.

The interface parameters and common settings were as follows: interface voltage: 1 kV; nebulizing gas flow: 3 L/minute; heating gas flow: 10 L/minute; drying gas flow: 10 L/minute; interface temperature: 400 °C; DL (desolvation line) temperature: 150 °C; heat block temperature: 500 °C; collision gas pressure 300 kPa. Compound-specific MRM parameters are shown in Table S7.

### 4.16. Western Blot Analysis

Tissues were lysed in an RIPA buffer containing 150 mM NaCl, 0.5% cholic acid, 0.1% SDS, 50 mM Tris-HCl, pH 8.0, 1% Triton X-100 and protease inhibitors. Tissue homogenates were agitated for 30 min at 4 °C and centrifuged for 10 min at 12,000× *g*. The supernatants were collected and stored at −20 °C until used. Protein concentration in lysates was determined using the Bradford assay (Biorad, Marne-la-Coquette, France). Loads were adjusted to 20 µg in sterile water with 4× Laemmli buffer, incubated at 95 °C for 5 min, and separated on Midi 4–15 % precast gels (Biorad, Marne-la-Coquette, France). Proteins were transferred onto nitrocellulose (Androgen receptor and CYP17A1) or PVDF (3βHSD) membranes, using the Transblot Turbo Transfer System (Biorad, Marne-la-Coquette, France). Transfer quality was confirmed by Ponceau S staining. Membranes were washed in TBS and then blocked in 5% non-fat milk (Androgen receptor and CYP17A1) or 5% BSA (3βHSD and β-actin) in TBS + 0.2% Tween-20 (TBST) for 30 min at 37 °C with a gentle rocking action. Membranes were incubated with primary antibodies (Table S5) overnight at 4 °C with rocking, washed three times in TBST for 5 min, and incubated with the appropriate secondary antibody (Table S5). Membranes were washed before incubation with ECL reagent (Clarity Western ECL Substrate, Biorad, Marne-la-Coquette, France) for 2 min and analysis with the Chemidoc imaging system (Biorad, Marne-la-Coquette, France) and the Image Lab^TM^ Software (v6.0.1, Biorad, Marne-la-Coquette, France). Β-actin was used to normalize proteins of interest.

Antibody dehybridization: Membranes were incubated for 30 min in a pre-warmed dehybridization solution containing 0.5 M Tris base (pH 6.8), 10% SDS, and 0.7% β-mercaptoethanol, in a 50 °C water bath. Then, membranes were rinsed abundantly with distilled water and rinsed twice with TBST. The efficiency of dehybridization was confirmed by the absence of labelling on the membrane after incubation with the secondary antibody.

### 4.17. Statistical Analysis

Statistical analyses were carried out with the GraphPad Prism software (v8.2.1, GraphPad Software Inc., La Jolla, CA, USA). Non-parametric Mann–Whitney tests, Chi^2^ tests and Kruskall–Wallis tests, followed by Dunn’s multiple comparison, were performed as specified in each figure. A value of *p* < 0.05 was considered statistically significant.

## Figures and Tables

**Table 1:** Biological information of the top 15 DEGs in each pattern (1–4 and 5–7) related to spermatogenesis. SAC—spindle assembly checkpoint.

Patterns 1–4								
ID	Gene	Adjusted F-Value	Pattern	Fold Change (Log(2))			Cell Type (Testis)	Function in Spermatogenesis
				D16	D22	D28		
25146	*Cyp17a1*	4.10 × 10^5^	P4	Down (−7.47)	Down (−7.13)	Down (−7.46)	Leydig cells [56]	Conversion of progesterone into dehydroepiandrosterone [56]
114215	*Insl3*	4.10 × 10^5^	P4	Down (−5.65)	Down (−4.63)	Down (−4.24)	Leydig cells and post-meiotic cells [57]	Testicular descent [57]
29460	*Tesk1*	5.20 × 10^5^	P4	Up (1.43)	Up (1.60)	Up (1.78)	Spermatocytes (late pachytene) to round spermatids [58,59,60]	Cytoskeleton reorganization [60]
362187	*Ccdc34*	9.00 × 10^5^	P4	Down (−1.39)	Down (−1.51)	Down (−1.38)	Meiotic and post-meiotic cells [61]	Regulation of cell cycle G2/M [61,62,63]
171304	*Kif11*	1.10 × 10^4^	P1	Down (−1.47)	Down (−1.53)	Down (−1.81)	Spermatogonia, spermatocytes, spermatids, and Sertoli cells [64]	Chromosomes separation [65,66]
296368	*Ube2c*	1.10 × 10^4^	P4	Down (−2.14)	Down (−2.05)	Down (−2.24)	Meiotic cells [67]	Regulation of metaphase/anaphase transition [68,69]
293733	*Incenp*	1.20 × 10^4^	P1	Down (−1.40)	Down (−1.25)	Down (−1.44)	Dividing cells [70,71,72]	Metaphase/anaphase progression [72] Chromatids cohesion [71]
25515	*Plk1*	1.20 × 10^4^	P4	Down (−1.82)	Down (−1.86)	Down (−1.77)	Spermatocytes (diplotene), spermatocytes II, and round spermatids [73,74]	Metaphase/anaphase transition checkpoint and chromosomes segregation [75,76] Phosphorylation of the central element of the synaptonemal complex [74] Cohesion of sister chromatids [77] Chromosomes alignment [78]
117524	*Ccnf*	1.40 × 10^4^	P1	Down (−1.78)	Down (−1.76)	Down (−1.67)	Dividing cells [79], spermatozoa [80]	Cell cycle [79] Potentially involved in proliferation and sperm motility [80]
498709	*Cks2*	1.40 × 10^4^	P4	Down (−1.36)	Down (−1.36)	Down (−1.56)	Meiotic cells [81]	Regulation of meiotic cell cycle, contribution to the control of the first metaphase/anaphase transition during mammalian meiosis [33,81,82]
81639	*Alox15*	1.70 × 10^4^	P1	Down (−7.34)	Down (−6.72)	Down (−5.39)	Spermatozoa [83]	Contribution to spermiogenesis with formation and resorption of the cytoplasmic droplet [83] Constituent of the oxidative stress pathway [84,85,86]
171576	*Bub1b*	1.70 × 10^4^	P1	Down (−1.26)	Down (−1.42)	Down (−1.49)	Mitotic and meiotic cells [87]	Regulation of the SAC and chromosomal alignment, interaction with Plk1 [78,88,89,90]
114494	*Ccna2*	1.70 × 10^4^	P1	Down (−1.54)	Down (−1.61)	Down (−1.68)	Spermatogonial stem cells (SSC), spermatogonia, spermatocytes (preleptotene), Sertoli cells [91,92,93]	Formation of the SSC pool [94] Regulation of microtubule dynamics during the rapid formation of the metaphase II spindle (by similarity with oocytes) [95]
303730	*Cbx2*	1.80 × 10^4^	P1	Down (−1.12)	Down (−1.22)	Down (−1.12)	Germ cells [96]	Establishment of the synaptonemal complex [96]
**Patterns 5–7**								
**ID**	**Gene**	**Adjusted** **F-Value**	**Pattern**	**Fold Change (Log(2))**			**Cell Type (Testis)**	**Function in Spermatogenesis**
				**D16**	**D22**	**D28**		
81687	*Mmp9*	4.10 × 10^5^	P7	Up (8.06)	Up (8.32)	Up (7.94)	Early SSC [97], gonocytes and Sertoli cells [98,99,100], spermatocytes and spermatids [101]	Cell junctions and BTB maintenance [101,102,103,104] Sperm motility [105]
25696	*Vldlr*	4.10 × 10^5^	P7	Up (2.28)	Up (2.90)	Up (2.48)	Spermatocytes (pachytene) and Leydig cells [106]	Regulation of meiosis [106,107]
252917	*Nr1d1*	4.70 × 10^5^	P7	Up (1.31)	Up (1.43)	Up (1.80)	Leydig cells [108] and germ cells [109]	Regulation of meiotic entry, Stra8/Nr1d1 balance [109] Stimulation of testosterone production and steroidogenic gene expression [108]
24484	*Igfbp3*	5.20 × 10^5^	P5	Up (3.62)	Up (3.69)	Up (3.70)	Sertoli cells [110], Leydig cells [111]	Regulation of testicular cell homeostasis via apoptosis [112], inhibition of IGF1 stimulation of steroidogenesis [111]
245920	*Cxcl10*	9.70 × 10^5^	P5	Up (2.35)	Up (2.78)	Up (2.72)	Leydig cells, T cells, and macrophages [113,114], peritubular and Sertoli cells during inflammation [115]	Role in inflammatory process, induction of germ cell apoptosis [115,116]
313210	*Abca1*	1.10 × 10^4^	P5	Up (1.47)	Up (1.27)	Up (1.69)	Leydig and Sertoli cells, round spermatids, spermatozoa [117,118]	Cholesterol transporter [118] Modulation of Sertoli cell phagocytosis [117] Impact on fertility [119]
114031	*Fstl3*	1.10 × 10^4^	P5	Up (1.94)	Up (2.04)	Up (2.08)	Leydig cells, spermatogonia, mature spermatids [120]	Regulation of gonadal development via interaction with activin [120,121,122]
84426	*Wnt4*	1.10 × 10^4^	P7	Up (3.34)	Up (3.90)	Up (4.18)	SSC [123] and Leydig cells [124]	Cell activity and apoptosis [123]
60350	*Cd14*	1.40 × 10^4^	P5	Up (2.28)	Up (2.53)	Up (2.85)	Putative SSC and early spermatogonia [125,126] Macrophages [127]	Inflammatory response signaling pathway [128] Immunoregulation of the testicular environment [129]
25125	*Stat3*	1.40 × 10^4^	P5	Up (1.29)	Up (1.60)	Up (1.71)	Gonocytes, pro-spermatogonia, round spermatids [130] Sertoli cells (Rete testis) [131]	Cell cycle regulation of G1 to S phase transition [132] Promotion of SSC differentiation [133,134] Cell viability [135] Assembly of the meiotic spindle (by similarity with oocytes) [136]
25425	*Ctsh*	1.40 × 10^4^	P7	Up (1.25)	Up (1.25)	Up (1.38)	Germ cells, Sertoli and Leydig cells [137,138]	Germ cells, Sertoli and Leydig cells [137,138]
89808	*Cx3cl1*	1.60 × 10^4^	P5	Up (1.51)	Up (2.04)	Up (2.42)	Interstitial tissue [139], Sertoli and Leydig cells, spermatogonia, spermatocytes and peritubular cells	Inflammation process [139] Stimulation of macrophage recruitment [140]
25589	*Kdr*	1.70 × 10^4^	P5	Up (1.57)	Up (1.74)	Up (1.91)	Spermatids, Sertoli and Leydig cells, lamina propria and blood vessels [141,142]	Participation to germ cell survival and enhancement of vascularization [143,144]
78968	*Srebf1*	1.70 × 10^4^	P5	Up (1.22)	Up (1.46)	Up (1.74)	Germ cells [145]	Regulation of cholesterol transport [146] Glucose homeostasis and fat metabolism [147]
24825	*Tf*	1.70 × 10^4^	P7	Up (3.25)	Up (3.52)	Up (2.95)	Sertoli cells, spermatocytes and early spermatids [148,149,150,151]	Regulation of sperm yield [152]

## Data Availability

Raw data files (fastq) and expression data for all transcripts have been submitted to the NCBI Gene Expression Omnibus (GEO) under accession number GSE197241. All data are also conveniently accessible through the ReproGenomics Viewer [195,196].

## Supplementary Materials

The following supporting information can be downloaded at: https://www.mdpi.com/article/10.3390/ijms23115893/s1.
