# Supplementary material for: Understanding the Underlying Molecular Mechanisms of Meiotic Arrest during In Vitro Spermatogenesis in Rat Prepubertal Testicular Tissue
Source: Int J Mol Sci. 2022 May 24;23(11):5893. doi: 10.3390/ijms23115893 (PMC9180380; doi:10.3390/ijms23115893)
Supplement: Supplementary file 1 [file ijms-23-05893-s001.zip › Supplementary Figure S1-S4, Tables S3-S7, Video captions.pdf]

Supplementary Material

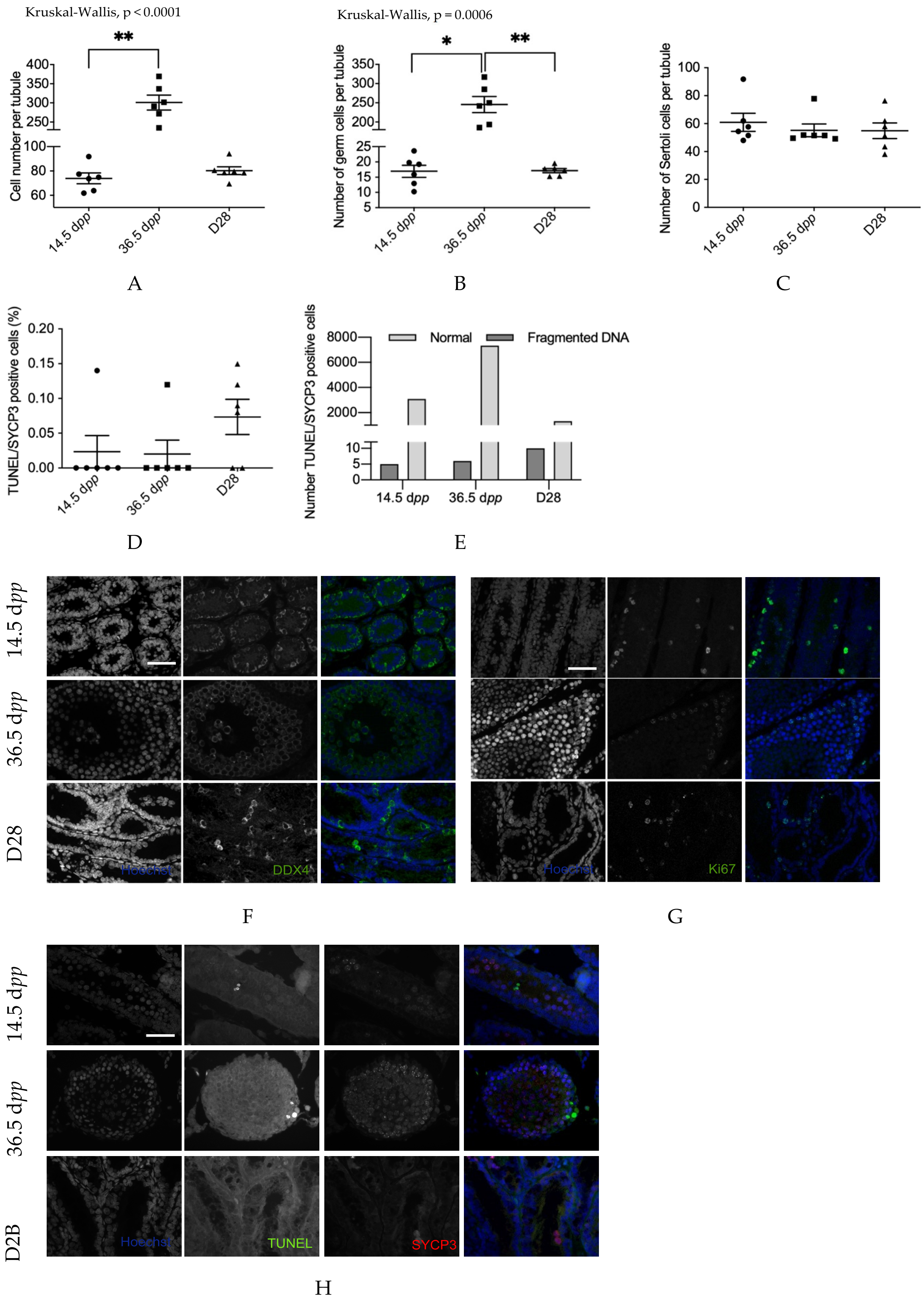

Figure S1. Histological and immunohistochemical evaluation of D28 in vitro cultured testicular tissues. Tissues cultured for 28 days (D28), tissues recovered from an age-matched invivo control (36.5 d*pp*) and from an immature in vivo control (14.5 dpp) were analyzed. (**A**) DDX4 immunostaining was performed to determine the number of cells per seminiferous tubule and the number of germ cells (**B**) and Sertoli cells (**C**) per tubule. (**D**) Percentage of TUNEL/SYCP3-positive cells. (**E**) Number of spermatocytes with or without DNA fragmentation. N = 6. For statistical analyses, Kruskal-Wallis tests followed with Dunn's post- test were applied. Data are expressed as mean ± s.e.m. * p value < 0.05; ** p value < 0.01. Localization by

immunofluorescence of DDX4 (**F**), Ki67 (**G**) and TUNEL/SYCP3 (**H**) in paraffin-embedded testicular tissue sections counterstained with Hoechst. Scale bar: 50 µm, Magnification: ×400.

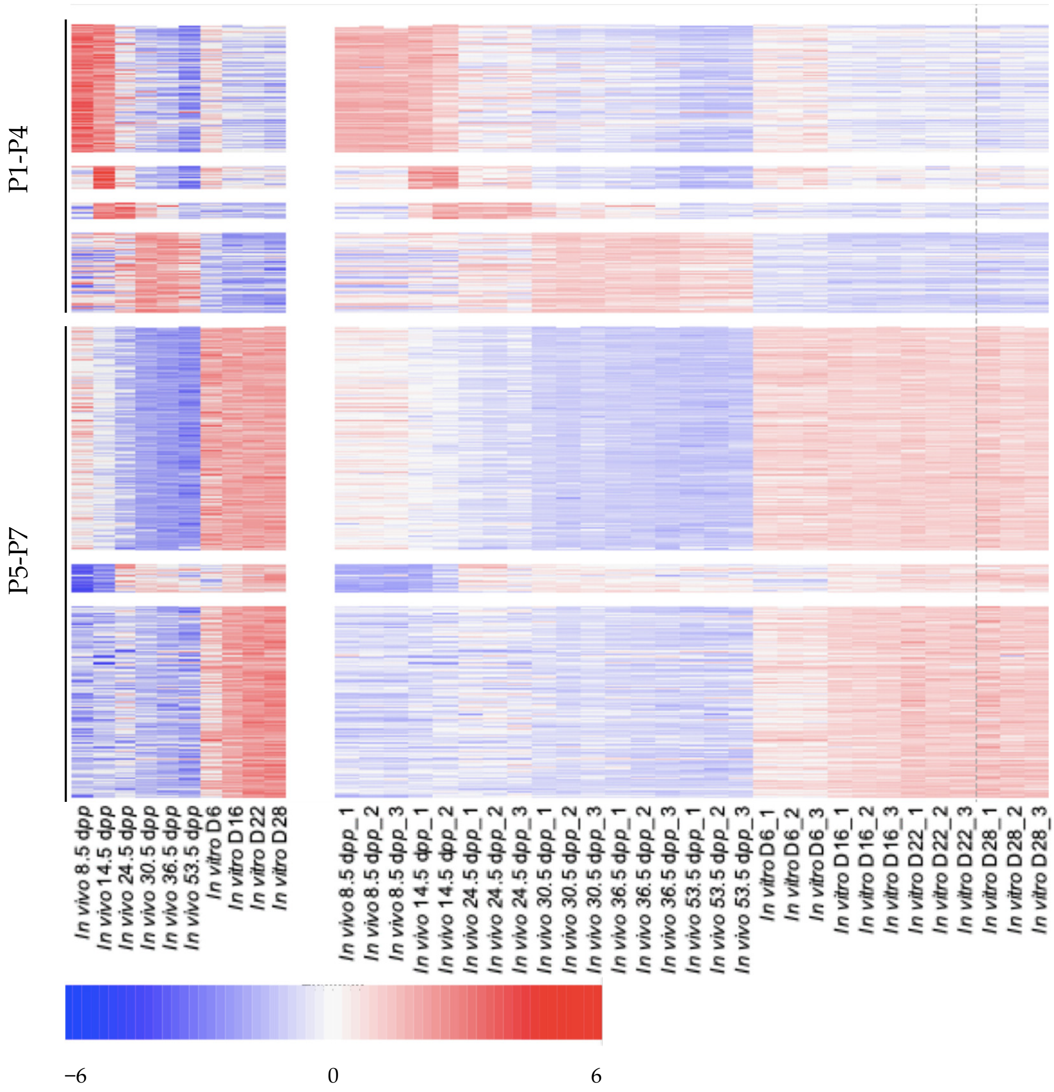

Figure S2. Transcriptomic analyses of in vitro and in vivo matured rat testicular tissues. A false-color heatmap summarizes the 7 patterns defining the global concentrations for transcriptsacross the entire sample set. Each line corresponds to a gene and each row to a replicate. A color scale is shown for standardized RPKM (Reads Per Kilobase of transcript, per Million mapped reads) values.

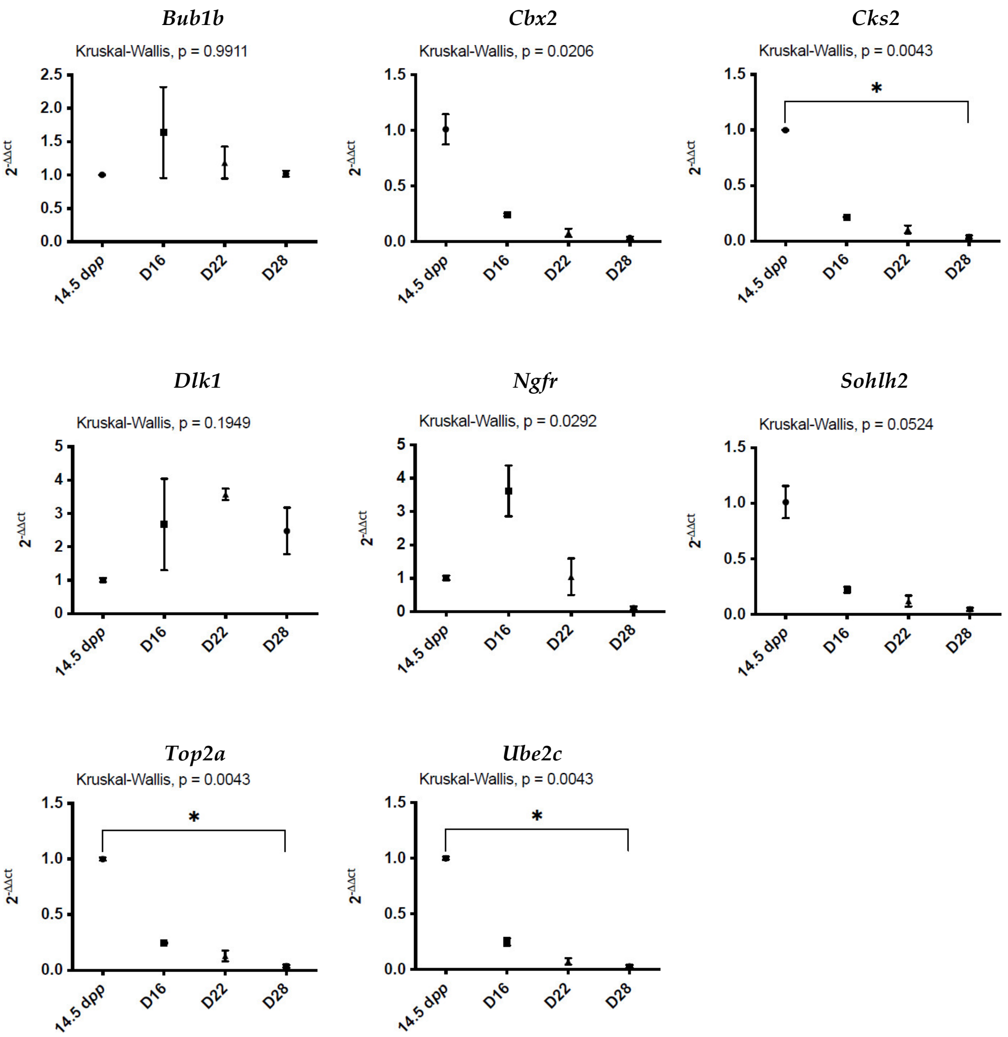

Figure S3. Relative mRNA levels of spermatogenesis-related genes (normalized to *Gapdh* and*Actb*) in in vitro cultured testicular tissues and 14.5 dpp in vivo controls. Data were obtained with RT-qPCR, n=3. For statistical analyses, Kruskal-Wallis tests followed with Dunn's post- test were applied. Data are expressed as mean ± s.e.m. * *p* value < 0.05. Dpp, days post-partum;RT-qPCR, Reverse transcription-quantitative Polymerase Chain reaction.

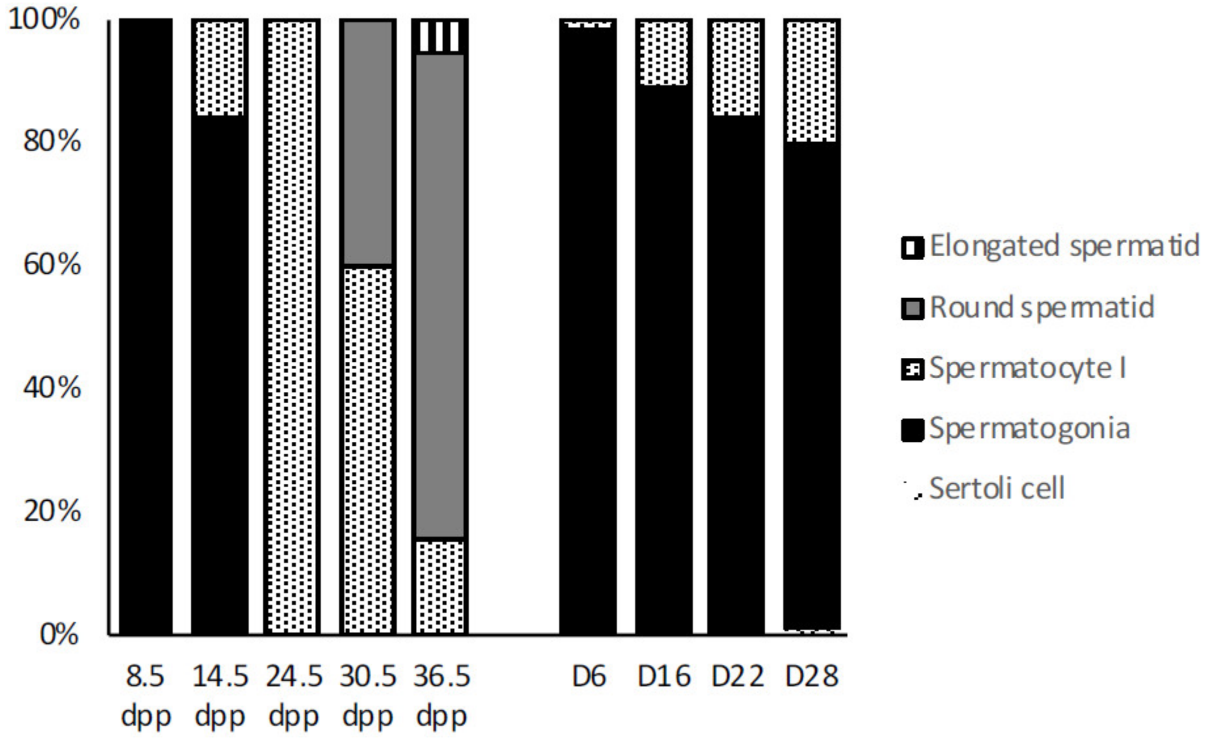

Figure S4. Most advanced stage of spermatogenesis in in vitro cultured tissues and age- matched in vivo controls. The culture starting point (8.5 dpp) was added. Contrary to other culture time points, tissues at D6 contained very few seminiferous tubules with meiotic cells. dpp, days postpartum.

Table S3. Biological information of the down-regulated and up-regulated genes related to spermatogenesis.NIA, No information available; SAC, Spindle assembly checkpoint.

ID	*Gene*	Ad-justedF-value	Pat-tern	Fold Change (Log(2))			Cell type (Testis)	Function in spermatogenesis
				D16	D22	D28		
25146	*Cyp17a1*	4.10E-05	P4	-7.47	-7.13	-7.46	Leydig cells [1]	Conversion of progesterone into dehydroepiandrosterone [1]
114215	*Insl3*	4.10E-05	P4	-5.65	-4.63	-4.24	Leydig cells and post-meiotic cells [2]	Testicular descent [2]
81687	*Mmp9*	4.10E-05	P7	8.06	8.32	7.94	Early spermatogonial stem cells (SSC) [3], gonocytes and Sertoli cells [4–6],spermatocytes and spermatids [7]	Cell junctions and blood-testis barrier(BTB) maintenance [7–10]<br>Sperm motility [11]
25696	*Vldlr*	4.10E-05	P7	2.28	2.90	2.48	Spermatocytes (pachytene) and Leydig cells [12]	Regulation of meiosis [12,13]
252917	*Nr1d1*	4.70E-05	P7	1.31	1.43	1.80	Leydig cells [14] and germ cells [15]	Regulation of meiotic entry,Stra8/Nr1d1 balance [15]<br>Stimulation of testosterone production and steroidogenic gene expression [14]
24484	*Igfbp3*	5.20E-05	P5	3.62	3.69	3.70	Sertoli cells [16], Leydig cells [17]	Regulation of testicular cell homeostasis via apoptosis [18], inhibition of IGF1 stimulation ofsteroidogenesis [17]
29460	*Tesk1*	5.20E-05	P4	1.43	1.60	1.78	Spermatocytes (late pachytene) to round spermatids [19–21]	Cytoskeleton reorganization [21]
362187	*Ccdc34*	9.00E-05	P4	-1.39	-1.51	-1.38	Meiotic and post-meiotic cells [22]	Regulation of cell cycle G2/M [22–24]
245920	*Cxcl10*	9.70E-05	P5	2.35	2.78	2.72	Leydig cells, T cells and macrophages [25,26], peritubular and Sertoli cells during inflammation [27]	Role in inflammatory process, inductionof germ cell apop-tosis [27,28]
313210	*Abca1*	1.10E-04	P5	1.47	1.27	1.69	Leydig and Sertoli cells, round spermatids,sper-matozoa [29,30]	Cholesterol transporter [30] Modulation of Sertoli cell phagocytosis[29]<br>Impact on fertility [31]
114031	*Fstl3*	1.10E-04	P5	1.94	2.04	2.08	Leydig cells, spermatogonia, mature spermatids [32]	Regulation of gonadal development *via* interaction with activin [32–34]
171304	*Kif11*	1.10E-04	P1	-1.47	-1.53	-1.81	Spermatogonia, spermatocytes, spermatidsand Sertoli cells [35]	Chromosomes separation [36,37]
296368	*Ube2c*	1.10E-04	P4	-2.14	-2.05	-2.24	Meiotic cells [38]	Regulation of metaphase/anaphase transition [39,40]
84426	*Wnt4*	1.10E-04	P7	3.34	3.90	4.18	SSC [41] and Leydig cells [42]	Cell activity and apoptosis [41]
293733	*Incenp*	1.20E-04	P1	-1.40	-1.25	-1.44	Dividing cells [43–45]	Metaphase/anaphase progression [45]<br>Chromatids cohesion [44]
								Metaphase/anaphase transition checkpoint and chromo-somes segregation [48,49]

25515	*Plk1*	1.20E-04	P4	-1.82	-1.86	-1.77	Spermatocytes (diplotene), spermatocytes II and round spermatids [46,47]	Phosphorylation of the central element of the synaptonemal complex [50] Cohesion of sister chromatids [51] Chromosomes alignment [52]
117524	*Ccnf*	1.40E-04	P1	-1.78	-1.76	-1.67	Dividing cells [53], spermatozoa [54]	Cell cycle [53]<br>Potentially involved in proliferation and sperm motility [54]
60350	*Cd14*	1.40E-04	P5	2.28	2.53	2.85	Putative SSC and early spermatogonia [55,56]<br>Macrophages [57]	Inflammatory response signalling pathway [58]<br>Immunoregulation of the testicular environment [59]
498709	*Cks2*	1.40E-04	P4	-1.36	-1.36	-1.57	Meiotic cells [60]	Regulation of meiotic cell cycle, contribution to the control of the first metaphase/anaphase transition during mammalian meiosis [60–62]
25425	*Ctsh*	1.40E-04	P7	1.25	1.25	1.38	Germ cells, Sertoli and Leydig cells [63,64]	Germ cells, Sertoli and Leydig cells [63,64]
25125	*Stat3*	1.40E-04	P5	1.29	1.60	1.71	Gonocytes, pro-spermatogonia, round spermatids [65]<br>Sertoli cells (Rete testis) [66]	Cell cycle regulation of G1 to S phase transition [67]<br>Promotion of SSC differentiation [68,69]<br>Cell viability [70]<br>Assembly of the meiotic spindle (by similarity with oocytes) [71]
89808	*Cx3cl1*	1.60E-04	P5	1.51	2.04	2.42	Interstitial tissue [72], Sertoli and Leydig cells, spermatogonia, spermatocytes and peritubular cells	Inflammation process [72] Stimulation of macrophages recruitment [73]
81639	*Alox15*	1.70E-04	P1	-7.34	-6.72	-5.39	Spermatozoa [74]	Contribution to spermiogenesis with formation and resorption of the cytoplasmic droplet [74]
								Constituent of the oxidative stress pathway [75–77]
171576	*Bub1b*	1.70E-04	P1	-1.26	-1.42	-1.49	Mitotic and meiotic cells [78]	Regulation of the SAC and chromosomal alignment, interaction with Plk1 [52,79–81]
114494	*Ccna2*	1.70E-04	P1	-1.54	-1.61	-1.68	SSC, spermatogonia, spermatocytes (preleptotene), Sertoli cells [82–84]	Formation of the SSC pool [85] Regulation of microtubule dynamics during the rapid formation of the metaphase II spindle (by similarity with oocytes) [86]
25589	*Kdr*	1.70E-04	P5	1.57	1.74	1.91	Spermatids, Sertoli and Leydig cells, lamina propria and blood vessels [87,88]	Participation to germ cell survival and enhancement of vascularization [89,90]
78968	*Srebf1*	1.70E-04	P5	1.22	1.46	1.74	Germ cells [91]	Regulation of cholesterol transport [92] Glucose homeostasis and fat metabolism [93]
24825	*Tf*	1.70E-04	P7	3.25	3.52	2.95	Sertoli cells, spermatocytes and early spermatids [94–97]	Regulation of sperm yield [98]
303730	*Cbx2*	1.80E-04	P1	-1.12	-1.22	-1.12	Germ cells [99]	Establishment of the synaptonemal complex [99]

60628	*Cxcr4*	1.90E-04	P7	2.58	2.61	2.72	Inhibition of CXCR4 signalling in testes of adult mice Impairment of SSC maintenance, leading to loss of the germline [100] Gonocytes and spermatogonia [101] Macrophages [102] Putative SSC [103]	Modulation of primordial germ cells migration and germ cell survival [104–106]
246273	*Trib3*	1.90E-04	P7	6.20	7.36	6.75	SSC [107]	Expression during early tissue development [107]
289054	*Aspm*	2.00E-04	P4	-1.66	-1.48	-1.65	Spermatocytes (leptotene to pachytene)	Spindle function [108] Necessary for germline maintenance [109]
114496	*Bik*	2.10E-04	P5	2.17	2.26	2.38	Testis [110]	Testis development, first wave of apoptosis [110]
24770	*Ccl2*	2.10E-04	P7	6.10	6.27	6.05	Interstitial cells [111] and peritubular cells [112]	Mobilization and migration of immune cells in the interstitium [113,114]
305984	*Cdca2*	2.10E-04	P4	-1.26	-1.32	-1.65	Dividing cells [115,116]	Chromosomes segregation [116] Chromatin remodeling [115,117]
59326	*Rapgef3*	2.10E-04	P5	1.27	1.55	1.60	Spermatozoa [118,119]	Element of the acrosome reaction signalling pathway [118,119]
24483	*Igf2*	2.20E-04	P5	3.49	3.19	3.04	NIA	Correlation with sperm motility and count [120,121] Biological redundancy insulin [122] Regulation of spermatogonial proliferation [123]
25026	*Adm*	2.30E-04	P7	4.14	4.58	4.87	Sertoli and Leydig cells [124,125]	Regulation of steroidogenesis [124]
363071	*Stra6*	2.50E-04	P1	-1.38	-1.45	-1.86	Sertoli cells [126]	Vitamin transport across BTB [126]
363088	*Ccnb2*	2.70E-04	P4	-1.30	-1.30	-1.35	Spermatocytes (pachytene, diplotene, metaphase), early round spermatids [127,128]	Not decisive for spermatogenesis [128] Expression counterbalanced by CCNB1 [129] Drives G2-M transition in mitosis with CDK1 (oocyte) [129,130]
266776	*Cst12*	3.00E-04	P6	1.58	2.39	2.57	Sertoli cells and spermatozoa [131,132]	May play a functional role in sperm maturation [132,133]
25116	*Hsd11b1*	3.00E-04	P7	2.87	3.48	3.82	Leydig cells [134]	Regulation of corticosterone and testosterone level [134]
25464	*Icam1*	3.20E-04	P5	1.82	1.95	2.13	Sertoli cells [135], spermatogonia, spermatids [136]	BTB and testicular homeostasis [7,135–137]
360243	*Top2a*	3.50E-04	P1	-1.44	-1.52	-1.87	Spermatogonia, spermatocytes, round spermatids and early elongating spermatids [138,139]	Appears as the unique enzyme responsible for transient double- stranded breaks in elongating spermatids [138] May be involved in mediating DNA modifications and maintaining nuclear organization during spermatogenesis [139–142]
360348	*Hsd3b1*	3.60E-04	P4	-3.04	-2.35	-1.98	Leydig cells [143,144]	Key enzyme for steroidogenesis [145]
294074	*Cep55*	3.70E-04	P1	-1.37	-1.35	-1.54	Sertoli cells [146], spermatocytes, spermatids and germ cell intercellular bridge [147]	Cytokinesis [146,148] Could induce germ cell apoptosis during overexpression [149]

25697	*Ctsl*	3.70E-04	P5	1.41	1.37	1.57	Inactive Ctsl induces atrophy of the seminiferous tubules and altered differentiation into pachytene spermatocytes [150]<br>Sertoli cells [151–154]<br>Dying or dead germ cells [155]	Involved in seminiferous tubules structure and germ cell differentiation [150]<br>Facilitation of spermatids movement toward the lumen of seminiferous
							Testis [156]<br>Intratubular cells [157]<br>Leydig cells, Sertoli cells, germ cells [158]	tubules [159], formation of Sertoli cell junctions [160]
315330	*Espl1*	4.10E-04	P4	-1.34	-1.26	-1.27	Mitotic and meiotic cells [161,162]	Removal of sister chromatids cohesion in anaphase by cleaving the cohesin complex, target phosphorylated Rec8 [161,163](by similarity to mitosis) [164–166] (mouse oocyte) [162,167]) (yeast)
292263	*Fbxo5*	4.10E-04	P1	-1.30	-1.31	-1.63	Testis [168]	Cell cycle regulation via Plk1 interaction [168–171]
294286	*Kifc1*	4.40E-04	P1	-1.56	-1.42	-1.50	Spermatocytes (pachytene), spermatids [172]	Nuclear shaping [172] and acrosome biogenesis [173,174]
295661	*Spc25*	4.40E-04	P1	-1.27	-1.33	-1.72	Dividing cells [175,176]	SAC cells [175,176]
25240	*Aqp1*	4.70E-04	P5	3.95	3.76	3.68	Rete testis epithelium (adult) [177,178]	Participation to the reabsorption of the tubal fluid [178,179]
81780	*Ccl5*	4.80E-04	P1	-1.94	-1.87	-1.52	Upregulation impacts negatively sperm fertilizing capacity [180]<br>Leydig cells, Sertoli cells and germ cells [181]<br>Immune cells [182]	Produced during inflammatory response [182,183]
25678	*Ddr1*	4.80E-04	P7	1.26	1.53	1.75	Testis [184], germ cells and peritubular cells [185]	Deregulation associated with non- obstructive azoospermia [185]
114592	*Aurkb*	4.90E-04	P1	-1.60	-1.92	-2.26	Spermatogonia, spermatocytes [186] Mitotic and meiotic cells [44,187]	Participation to homologous chromosomes alignment and segregation [188–190] (oocyte)<br>[191,192]
304951	*Nuf2*	5.70E-04	P1	-1.09	-1.18	-1.18	Dividing cells [193]	Cell cycle progression, chromosomes segregation [193–195]
25203	*Ccnb1*	6.00E-04	P1	-1.38	-1.56	-1.63	The meiotic G2/M transition is associated with high levels of Cyclin B1 [196] Spermatogonia, spermatocytes (pachytene, II), round spermatids and Sertoli cells [196–199]	Potentially essential for SSC renewal and spermatocytes differentiation [199,200]
29680	*Cyp11a1*	6.00E-04	P4	-3.17	-3.76	-3.25	Leydig cells [144,201]	Enzyme of steroidogenesis, determines the biosynthetic capacity of Leydig cells [144,201]
114482	*Nek2*	6.10E-04	P4	-1.77	-1.53	-1.72	Spermatocytes (pachytene and diplotene), early round spermatids [202]	Meiotic progression [203], chromatin condensation [204] and SAC [205]
298441	*Nasp*	6.30E-04	P4	-1.22	-1.12	-1.40	Spermatocytes I, round spermatids and spermatozoa [206,207]	Potentially related to acrosome reaction [207]<br>Histone transport protein [208]
24925	*Adrb1*	6.80E-04	P7	3.32	3.75	4.03	Sertoli and Leydig cells [209], testis [210]	Stimulation of testosterone production [209]

54320	*Pdpn*	6.80E-04	P5	1.41	1.34	1.48	Sertoli cells [211]	Proliferation and maturation of Sertoli cells, stimulation of Androgen Binding Protein production [211]
291023	*Id4*	7.10E-04	P4	1.17	1.68	1.75	SSC [212]	SSC self-renewal [212–214]
619575	*Sohlh2*	7.20E-04	P3	-1.37	-1.54	-1.52	Overexpression induces a lower proliferation level (by similarity with female cells) [215] Meiotic cells [216] Spermatogonia, Sertoli cells, Leydig cells and smooth muscle of blood vessel wall [217,218]	Critical for the formation of synaptonemal complexes via the regulation of Sycp1 expression [216] Progression and viability of differentiating spermatogonia via Kit stimulation [217,219] Regulation of meiotic entry by interaction with STRA8 [220]
24854	*Clu*	8.80E-04	P7	1.09	1.75	2.08	KO = uKO mice were fertile and had essentially normal spermatogenesis with the exception of some incomplete spermiation after stage VIII Spermatozoa, Sertoli cells and degenerating germ cells [221–224]	Possibly involved in sperm maturation and capacitation [225]
54238	*Cdkn2c*	9.00E-04	P4	-2.13	-1.96	-2.46	Spermatocytes (pachytene), round spermatids, elongated spermatids and Leydig cells [226,227]	Sperm cell development and proliferation [228] Regulation of spermatogenesis via inhibition of CDKs, mitotic exit and normal meiotic maturation of spermatocytes [226]
81686	*Mmp2*	9.20E-04	P5	2.13	1.85	1.88	Spermatogonia, spermatocytes, round spermatids, spermatozoa and Sertoli cells [229–234]	Regulation of SSC stemness [235] Migration of spermatogonia and spermatocytes [232] Regulation of BTB tight junctions
								[8,236] Regulation of the elongated spermatid dynamics [237]
58919	*Ccnd1*	9.90E-04	P2	-1.26	-1.32	-1.25	Gonocytes and spermatogonia [238] Sertoli cells [239]	Modulation of Sertoli cell proliferation [240,241] Regulation of SSC renewal and differentiation [242–244]
64557	*Bard1*	1.10E-03	P1	-1.29	-1.17	-1.35	Spermatogonia, spermatocytes, spermatids [245]	Modulation of meiotic recombinations [246]
293844	*Haus7*	1.10E-03	P5	1.23	1.18	1.00	KO led to spindle defects, chromosomes misalignment and disruption of γ-tubulin localization in the spindle poles (by similarities with oocytes) [247] Spermatocytes [248]	Could be associated with oligoazoospermia [249] Involved in spindle formation during meiotic maturation (by similarity with oocytes) [247]
116744	*Lpar1*	1.10E-03	P5	1.23	1.21	1.23	Germ cells [250]	Related to testosterone production and germ cell survival [250,251]
25728	*Apoe*	1.30E-03	P5	1.91	1.60	1.94	Sertoli cells and germ cells, interstitial cells [252–255]	Regulation of cholesterol biosynthesis and steroidogenesis [256,257] Lipid transport for sperm maturation [258]

83785	*Vegfa*	1.30E-03	P7	3.01	3.98	3.66	KO induces subfertility and a reduced sperm count [259]<br>Germ cells, Sertoli cells and Leydig cells [89,260,261]	Support germ cell production [261,262] Development of vasculature and seminiferous cords [260,263]
79131	*Fabp3*	1.90E-03	P5	1.06	1.02	1.02	Testis [264]	Associated with the functional differentiation of interstitial cells [265]
89829	*Socs3*	1.90E-03	P5	1.37	2.17	2.24	Sertoli cells [266]	Involved in inflammatory response pathway [267]<br>Linked with female infertility [268]
24778	*Slc2a1*	2.00E-03	P5	1.34	1.70	1.69	Blood vessels [269], spermatocytes and spermatids [270]	Glucose transporter [270]
685161	*Eppin*	2.20E-03	P3	-2.91	-1.48	-1.50	Spermatozoa [271,272]<br>Round spermatids, elongated spermatids [273]	Control of sperm motility and acrosomal reaction via modulating internal calcium levels [272,274,275]
24167	*Adcyap1r1*	2.50E-03	P5	2.20	2.13	1.92	Leydig cells, endothelial cells, primary spermatocytes [276]	Probably related to fertility rate [277]
140588	*Gli3*	2.50E-03	P1	-1.64	-1.54	-1.53	Sertoli cells, spermatogonia [278], spermatocytes and round spermatids [279]	Inhibition of Sertoli cell proliferation [280]
64515	*Cdc20*	2.60E-03	P4	-1.73	-1.70	-1.59	Mutation led to dysfunction during the transition from metaphase to anaphase [281] Spermatocytes (pachytene), round spermatids, elongated spermatids [282]	Involved in metaphase/anaphase transition [283]<br>Critical for spindle assembly and chromosomes segregation [284,285] (by similarity with oocytes)
292071	*Cdt1*	2.90E-03	P1	-1.19	-1.19	-1.42	Testis [286]	Contribution to genomic stability [287]
29366	*Serpine2*	3.20E-03	P5	1.21	1.18	1.30	Germ cells, Leydig cells [288]	Prevention of cholesterol efflux [288]
313484	*Dmrtb1*	3.30E-03	P4	-1.57	-2.52	-1.48	Spermatogonia, spermatocytes (preleptotene to pachytene) [289,290]	Coordination of the mitosis/meiosis transition [290]
304966	*Fcgr3a*	3.40E-03	P7	3.72	4.39	4.79	Natural killer cells (by similarity with female) [291]l<br>Lymphocytes [292]	Involved in immunological sterility [292] Female infertility [293]
84392	*Kif3a*	4.50E-03	P4	-1.42	-1.51	-1.63	Spermatocytes, round spermatids, elongating spermatids, spermatozoa [294]	Microtubular transport and formation of sperm [295,296]
24182	*Agtr2*	8.10E-03	P5	1.22	1.01	0.98	Testis [297], spermatozoa [298]	Probably related to tissue growth [297,299], correlated with sperm motility [298]
54237	*Cdk1*	8.30E-03	P1	-1.75	-1.97	-2.46	Upregulation of CDK1 inhibits separase action [163]<br>Spermatogonia and spermatocytes (I and II) [83,300,301]	Progression of the meiotic division [196,300,302–304]

301701	*Ndc80*	8.50E-03	P4	-1.84	-1.57	-1.81	Mitotic and meiotic cells [305] (by similarity with oocytes) [306], mitosis [307] (by similarity with oocytes)	Chromosomes alignment and spindle organization [305,307] (by similarity with oocytes) [205,308], somatic cells [309,310], mitosis [311–313] Involved in G2-M transition [314]
294503	*Nodal*	9.80E-03	P5	2.20	1.94	1.87	Germ cells [315,316]	Regulation of male germ cell potency [315,317,318] and Sertoli cell proliferation [316]
690393	*Tex12*	1.00E-02	P3	-4.04	-3.47	-2.08	Spermatocytes [319,320]	Component of the central element and structure of the synaptonemal complex, pairing of homologous chromosomes by the establishment of SYCP1 [319,321,322] Phosphorylated by PLK1, for the disassembly of the synaptonemal complex [50]
24775	*Shbg*	1.10E-02	P7	0.89	1.62	1.49	Sertoli cells [323,324] and Leydig cells [325,326] Germ cells (endocytosis) [327]	Androgen transport protein, facilitation of the transport of testosterone or dihydrotestosterone into seminiferous tubules [325] Influence on the dynamic of germ cell proliferation [328] Extracellular transport of androgens [323]
366492	*Epha2*	1.20E-02	P5	1.04	1.26	1.44	SSC [329]	Critical modifier of self-renewal signals in SSC [329]
292206	*Trip13*	1.20E-02	P2	-1.68	-1.58	-1.74	Upregulation probably silences the mitotic checkpoint [330] Mitotic cells and spermatocytes [330–332]	Participation to the formation of the synaptonemal complex, efficient synapsis of sex chromosomes and for sex body formation, crossover formation and recombination [331–333] Involved in chromosomal instability [334] Regulation of DSBs repair [335]
25353	*Spp1*	2.40E-02	P5	1.89	1.91	1.96	Spermatogonia to early pachytene spermatocytes [336] SSC [337]	Suggests a role as an adhesive protein binding to the basement membrane and adjacent Sertoli cells [336] Potentially involved in SSC proliferation [337] Initiation of meiotic recombination, DSB (yeast) [338–341]
25056	*Rbp1*	3.70E-02	P5	1.52	1.32	1.75	Reduced CRBP I mRNA levels in vitamin A deficient testis [342] Spermatocytes, Sertoli cells and peritubular cells [248,343,344]	May regulate the intracellular concentration of retinoids [343]
25345	*Prm2*	4.00E-02	P4	-0.45	-3.51	-0.45	Round spermatids and elongating spermatids [345,346]	Essential for sperm DNA compaction [346]
24840	*Tnp2*	4.20E-02	P4	-2.40	-2.60	-3.84	Round spermatids and elongating spermatids [345,347]	Allows the histone to protamine transition in spermatids [348]
83722	*Plk2*	4.30E-04	P6	2.156504	2.102189	1.878657	Spermatocytes [47,349], at the crossover sites [350]	Stabilization of the synaptonemal complex [349]
24596	*Ngfr*	4.90E-04	P5	2.185249	1.833839	1.468167	Sertoli cells [351]	Regulation of entry into the second meiotic division [352] Regulation of meiosis via androgen

								pathway and fixation to NGF produced by germ cells [351]
364382	*Prmt5*	1.00E-03	P3	-1.21228	-1.27504	-1.18728	Germ cells [353,354]	Indispensable for germ cell survival and meiotic progression [353,354]
287634	*Eme1*	1.90E-03	P4	-2.33741	-1.9649	-1.72282	Spermatocytes [355]	Component of the crossover pathway [355–357]
289175	*Slc19a2*	5.60E-03	P6	1.049012	1.210315	1.180235	NIA	Necessary for survival of pachytene spermatocytes [358,359]

Table S4. IPA analysis of the 594 differentially expressed genes in in vitro cultured tissues(D28).

Upstream Regulator	Molecule Type	Activation z-score	p-value of overlap	Target Molecules in Dataset	Mechanistic Network
ZBTB17	Transcriptionreg-ulator		4.56E-30	ASPM,AURKB,BUB1B,CCNA2,CCNB1,CCNB2,CDC20,CDCA8,CDK1,CENPF,CEP55,CKAP2,ECT2,FOXM1,HMMR,IQGAP3,KIF11,KIF18B,KIF22,MKI67,NDC80,NUF2,NUSAP1,PBK,PIMREG,PLK1,PRC1, TOP2A,UBE2C	166 (7)
TNF	Cytokine	6.201	2.15E-26	ABCA1,ACTA2,ADCY5,ADM, ADORA2A,AOC3,APLN,APOE,ARSI,ATF3,B2M,BHLHE40, BIK,CASP4,CCL11,CCL2,Ccl2,CCL5,Ccl7,CCND1,CD14,CDH13,CDH2,CEBPD,CFB,CH25H,CLU,CNN1,CTSK,CTSS, CX3CL1,CXCL10,CXCL13,CXCL2,CXCR4,CYP11A1,CYP17A1,DUSP5,EFNA1,FCGR2B,G0S2,GADD45A,GM2A,H19,HLA-A,ICAM1,IER3,IGF2,IGFBP3,IL4R,ISG15,JUNB,LRRC8C,LTBP2,MGP,MGST1,MGST2, MMP12,MMP2,MMP9,MYLK, NQO1,OSMR,PDPN,PENK,PLA2G2A,PLSCR1,PRSS23,RASD1,RBP1,SAT1,SERPINB9,SERPINE2,SLC2A1,SOCS3,SPSB1,SREBF1,STAT3,STEAP4,STMN1,TAGLN,TF,TGM2,TMEM176B,VEGFA	177 (17)
CEBPB	Transcriptionreg-ulator	-2.669	1.15E-25	ACTA2,APLN,APLNR,AURKB,BARD1,BUB1B,C1QC,C3AR1,Ccl2,CCL5,CCNA2,CCNB2, CCND1,CD14,CDC20,CDCA3,CDCA8,CDH3,CDK1,CDKN	245 (18)

				2C,CDT1,CEBPD,CXCL10,CXCR4,CYP11A1,CYP17A1,DEPDC1,DIAPH3,DLGAP5,ESPL1,FBLN1,FCGR1A,FCGR3A/FCGR3B,FZD1,GADD45A,HBB,HMMR,HSD11B1,IER3,IGFBP3,KIF18B,KIF22,LGMN,LMNB1,MELK,MKI67,MXD3,NASP,PLK1,SPC25,SPP1,SREBF1,STAT3,STMN1,TACC3,TF,TK1,TOP2A,TRIB3,TRIP13,VLDLR	
CSF2	Cytokine	-1.175	2.91E-25	ABCA1,BUB1B,CCL2,Ccl2,CCNA2,CCNB1,CCNF,CD14,CDC20,CDCA2,CDCA3,CDCA8,CDK1,CSF2RB,CXCL10,E2F7,FBXO5,FCGR2B,FOXM1,HJURP,ICAM1,IER3,IFITM3,JUNB,KIF11,LCP1,MKI67,MMP9,NUSAP1,OSMR,PLK1,PRC1,RBBP4,RECQL4,REG3G,SLC2A1,SNTB1,SOCS3,SPC25,SPP1,SREBF1,STMN1,TGM2,TOP2A,TRIP13,UBE2C,UHRF1	185 (18)
LDLR	Transporter	1.664	6.9E-24	ABCA1,APOE,AURKB,BUB1B,CCL2,Ccl2,CCL5,Ccl7,CCNA2,CCNB1,CCNB2,CCND1,CDCA3,CDK1,CENPA,CENPF,CFB,CX3CL1,E2F7,FCGR1A,FOXM1,GAPDH,GJA4,ICAM1 ,IER3,IRF7,LYZ,mir-23,MKI67,MMP2,MMP9,NUSAP1,PFKL,PLK1,PRC1,SLC2A1,SPP1,SREBF1,TOP2A,UBE2C,UHRF1,VLDLR	156 (14)

TP53	Transcrip-tionregulator	3.98	9.41E-24	ACSL6,ACTA2,AD-GRB1,ALOX15,APOE,ATF3,AURKB,BCL6B,BHLHE40,BIK,BUB1B,C1QC,CASP4,Ccl2,CCL5,CCNA2,CCNB1,CCNB2,CCND1,CD82,CDC20,CDH3,CDK1,CDT1,CENPF,CEP55,CKAP2,CKMT2,CLIC4,CLU,CNN1,CPT1B,CXCL10,CXCL2,DDR1,DGKA,DLGAP1,DUSP4,DUSP5,EPHA2,ESPL1,FABP3,FGF7,FKBP5,FOXM1,FSTL3,FUCA1,GADD45A,GADD45B,GAPDH,GPR157,GTSE1,H2BC5,HMMR,ID3,IER3,IGF2,IGFBP3,IL4R,INKA2,IRF7,ISG15,JUNB,KCNJ2,KCNJ4,KDR,KIFC1,LY6H,LYZ,MGST2,mir-23,mir-27,MKI67,MMP2,MMP23B,MMP9,NDC80,NDRG1,NR2F1,PBK,PECAM1,PLK1,PLK2,PRAG1,PRC1,PTPRM,RBBP4,RECQL4,RFC4,RRM1,RUNX1,SAT1,SERPINB9,SERPINE2,SESN2,SLC19A2,SLC2A1,SPC25,SPP1,SREBF1,STAT3,STMN1,TGM2,THBS1,TOP2A,TRIB3,TYMS,UBE2C,UHRF1,UNC5B,VASN,VEGFA,VLDLR,WNT10A	231 (23)

TGFB1	Growth factor	2.01	2.54E-23	ACTA2,ADM,AQP1,ASPM,BARD1,BHLHE40,BUB1B,CASP4,CCL11,CCL2,Ccl2,CCL5,Ccl7,CCNA2,CCNB1,CCNB2,CCND1,CDC20,CDH2,CDK1,CDKN2C,CDT1,CENPA,CENPF,CKS2,CLDN4,CNN1,CXCL10,CXCL2,CXCR4,CYP11A1,CYP17A1,ELN,ENPP1,ESPL	204 (20)
				1,FAM107A,FBLN5,FSTL3,FZD1,HTRA1,ICAM1,IER3,IGF2,IGFBP3,JUNB,JUP,KDR,LTBP2,mir-196,MKI67,MMP12,MMP2,MMP9,MXD3,NDC80,PECAM1,PRC1,RFC4,S1PR3,SEMA7A,SOCS3,SPP1,STAT3,TAGLN,TGM2,THBS1,TOP2A,VEGFA	
FOXM1	Transcription regulator	-2.555	3.68E-23	AURKB,BUB1B,CCNA2,CCNB1,CCNB2,CCND1,CCNF,CDC20,CDCA2,CDCA8,CDH2,CDK1,CENPA,CENPF,CKS2,CTNNAL1,FZD1,GTSE1,KDR,MKI67,MMP2,MMP9,NUF2,PECAM1,PLK1,PRC1,PRR11,SKP2,STAT3,STMN1,VEGFA	245 (19)
NR1H3	Ligand- dependent nuclear receptor	0.381	1.59E-22	ABCA1,APOE,ATF3,AURKB,BUB1B,CCL2,Ccl2,CCL5,Ccl7,CCNA2,CCNB1,CCNB2,CDCA3,CDK1,CENPA,CENPF,CFB,CH25H,CX3CL1,CXCL10,CXCL2,E2F7,FCGR1A,FOXM1,HSD11B1,IFIT2,IRF7,LYZ,MELK,MKI67,MMP9,MYLIP,N	173 (16)

				USAP1,PLK1,PRC1,SREBF1, TOP2A,UBE2C,UHRF1	
ERBB2	Kinase	0.202	5.2E-22	ANGPT2,ASPM,ATF3,BARD1 ,BHLHE40,BUB1B,CAVIN2,C CL11,CCL2,CCL5,Ccl7,CCNA 2,CCNB1,CCNB2,CCND1,CD 82,CDC20,CDCA2,CDCA3,C DCA7,CDCA8,CDH2,CDK1,C DKN2C,CDT1,CENPA,CENP F,CKS2,CLDN4,CST8,CXCL1 0,E2F7,ESPL1,ETV1,FSTL3, GALNT18,HOMER2,HTRA1,I CAM1,IL17B,JUNB,JUP,LTB P2,LUM,mir-23,mir-	263 (24)
				27,MKI67,MMP12,MMP16,M MP9,MXD3,NDC80,NOTCH4 , NUP210,PENK,PRC1,RFC4, RRM1,SLC2A1,STAT3,STEA P4,TAGLN,THBS1,TK1,TOP2 A,TYMS,UNC5B,VEGFA	

Table S5. Antibodies used in the current study**.** IF, Immunofluorescence; IHC,ImmunoHistoChemistry; WB, Western-Blot.

Category	Antibody/protein	Host	Dilution	Reference	Supplier
Primary	3□HSD	Mouse	1:1000 (WB)	sc-515120	Santa Cruz Biotechnologies
	γH2AX	Mouse	1:200 (IHC)<br>1:500 (IF)	05-636	Merck
	b-actin	Mouse	1: 5000 (WB)	Ab8226	Abcam
	Androgen receptor	Rabbit	1:5000 (WB)	ab133273	Abcam
	CD68	Mouse	1:200 (IF)	MCA341R	Biorad
	CYP17A1	Rabbit	1:10000 (WB)	ab231794	Abcam
	DDX4	Rabbit	1:800 (IF)	ab13840	Abcam
	Ki67	Rabbit	1:500 (IF)	ab16667	Abcam

	SYCP3	Rabbit	1:200 (IF)	ab15093	Abcam
	SYCP3	Mouse	1:200 (IF)	ab97672	Abcam
	SYCP1	Rabbit	1:200 (IF)	ab175191	Abcam
Secondary	Anti-rabbit (Alexa Fluor® 488-conjugated)	Goat	1:200 (IF)	ab150077	Abcam
	Anti-rabbit (Alexa Fluor® 594-conjugated)	Goat	1:200 (IF)	ab150080	Abcam
	Anti-mouse (Alexa Fluor® 488-conjugated)	Goat	1:200 (IF)	ab150113	Santa Cruz Biotechnologies
	Anti-mouse (Biotin-conjugated)	Goat	1:200 (IF)	ab6788	Abcam
	Polyvalent (Biotin-conjugated)	Goat	Prediluted (IHC)	TP-60-BN	ThermoFisher Scientific
	Anti-mouse (HRP)	Goat	1:5000 (WB)	31430	Invitrogen
	Anti-rabbit (HRP)	Goat	1.5000 (WB)	A16110	Invitrogen
Control IgGs		Rabbit	1:200 (IF, IHC)	02-6102	Invitrogen
		Mouse	1:200 (IF, IHC)	Sc-2025	Santa Cruz Biotechnologies
Alexa Fluor® 594-conjugated streptavidin		/	1:200 or 1:100 (IF)	S11227	Invitrogen

Table S6. Primers and parameters used for RT-qPCR. bp, base pair; RT-qPCR, Reversetranscription-quantitative Polymerase Chain reaction.

Gene Name	Forward Primer (5′→3′)	Reverse Primer (5′→3′)	Tm (°C)	Size (bp)
Cbx2	CAAGGGCAAGCTGGAGTACCT	TCGGGTCCAAGATGTTCTCTTC	80.2	89
Cks2	GGAGGAGACTTGGTGTTCAACAG	AATATGCGGTTCTGGCTCATG	74.5	71
Top2a	GAACTGGCTTCAAATCAGTATGTGA	CCTTATATGTCTGGGTCCATGTTCT	74.0	109
Ube2c	AGCCCTTTGAACACACATGCT	TGGTTGGAGACCTGCTTTGAATA	75.5	95
Dlk1	GTGAAGAACCATGGCAGTGTGT	GCAAGCCCGAATATCTATTTCG	76.5	74
Bub1b	ACTACAGAAGTCATCCCTGCAAATG	ATGATGGCATCTTCACTCAAAGG	73.0	92
Sohlh2	TCCCTTCAGAGCAATAAGAGGTTT	CATAGCATCTCCCCTCTGTGATG	77.3	93
Ngfr	GGGCCTTGTGGCCTATATTG	GCGCCTTGTTTATTTTGTTTGC	78.0	63
Actb	ACCGTGAAAAGATGACCCAGAT	CACAGCCTGGATGGCTACGTA	76.0	74
Gapdh	CAGCCTCGTCTCATAGACAAGATG	CAATGTCCACTTTGTCACAAGAGAA	79.0	106

Table S7. Compound-specific Multiple Reaction Monitoring parameters.

Name Q = Quantifier/q = qualifier	Precursor Ion (m/z)	Product Ion (m/z)	Q1 Pre Bias (V)	Collision Energy (CE)	Q3 Pre Bias (V)
Testosterone-13C3-Q	292.1	112.1	-18	-23	-23
Testosterone-13C3-q	292.1	100.15	-18	-23	-11
Testosterone-Q	289.1	109.2	-17	-26	-12
Testosterone-q	289.1	97.15	-17	-22	-18

Video S1: SYCP3/γH2AX immunofluorescence staining of meiotic spread preparations from 36.5 dpp controls. Representative SYCP3 (red)/γH2AX (green) immunostaining of two pachytene spermatocytes from 36.5 dpp testicular tissues. The spermatocytes contain normal synaptonemal complexes and a γH2AX-positive XY body. Magnification: ×630, Zoom 4.

Video S2: SYCP3/γH2AX immunofluorescence staining of meiotic spread preparations from D28 cultured tissues. Representative SYCP3 (red)/γH2AX (green) immunostaining of a pachytene spermatocyte from D28 testicular explants. The spermatocyte contains compact synaptonemal complexes, multiple γH2AX-positive foci localizing alongside the synaptonemalcomplexes and a γH2AX-positive XY body. Magnification: ×630, Zoom 4.

Video S3: SYCP3/SYCP1 immunofluorescence staining of meiotic spread preparations from 36.5 d*pp* controls. Representative SYCP3 (red)/SYCP1 (green) immunostaining of a pachytenespermatocyte from 36.5 d*pp* testicular tissues. The spermatocyte contains normal synaptonemalcomplexes, with a colocalization of the lateral (SYCP3) and the central (SYCP1) elements (except for the sex chromosomes). Magnification: ×630, Zoom 4.

Video S4: SYCP3/SYCP1 immunofluorescence staining of meiotic spread preparations from D28 cultured tissues. Representative SYCP3 (red)/SYCP1 (green) immunostaining of two pachytene spermatocytes from D28 testicular explants. The spermatocytes contain incompletely formed synaptonemal complexes, with dim SYCP1 labelling along SYCP3filaments. Magnification: ×630, Zoom 4.

References

1. Aghazadeh, Y.; Zirkin, B.R.; Papadopoulos, V. Pharmacological Regulation of the Cholesterol Transport Machinery in Steroidogenic Cells of the Testis. *Vitam. Horm.* **2015**, *98*, 189–227, doi:10.1016/bs.vh.2014.12.006.
2. Sansone, A.; Kliesch, S.; Isidori, A.M.; Schlatt, S. AMH and INSL3 in Testicular andExtragonadal Pathophysiology: What Do We Know? *Andrology* **2019**, *7*, 131–138, doi:10.1111/andr.12597.
3. Lee, W.-Y.; Do, J.T.; Park, C.; Kim, J.H.; Chung, H.-J.; Kim, K.-W.; Gil, C.-H.; Kim,N.-H.; Song, H. Identification of Putative Biomarkers for the Early Stage of Porcine Spermatogonial Stem Cells Using Next-Generation Sequencing. *PloS One* **2016**, *11*, e0147298, doi:10.1371/journal.pone.0147298.
4. Robinson, L.L.; Sznajder, N.A.; Riley, S.C.; Anderson, R.A. Matrix Metalloproteinasesand Tissue Inhibitors of Metalloproteinases in Human Fetal Testis and Ovary. *Mol. Hum. Reprod.* **2001**, *7*, 641–648, doi:10.1093/molehr/7.7.641.
5. Kumar, S.; Tinson, A.; Mulligan, B.P.; Ojha, S. Gelatin Binding Proteins inReproductive Physiology. *Indian J. Microbiol.* **2016**, *56*, 383–393, doi:10.1007/s12088-016-0618-0.
6. Kurzawski, M.; Kaczmarek, M.; Kłysz, M.; Malinowski, D.; Kazienko, A.; Kurzawa, R.; Droździk, M. MMP2, MMP9 and TIMP2 Polymorphisms Affect Sperm Parametersbut Not Fertility in Polish Males. *Andrologia* **2017**, *49*, doi:10.1111/and.12654.
7. Lydka, M.; Bilinska, B.; Cheng, C.Y.; Mruk, D.D. Tumor Necrosis Factor α-MediatedRestructuring of the Sertoli Cell Barrier in Vitro Involves Matrix Metalloprotease 9 (MMP9), Membrane-Bound Intercellular Adhesion Molecule-1 (ICAM-1) and the Actin Cytoskeleton. *Spermatogenesis* **2012**, *2*, 294–303, doi:10.4161/spmg.22602.
8. Siu, M.K.Y.; Lee, W.M.; Cheng, C.Y. The Interplay of Collagen IV, Tumor Necrosis Factor-α, Gelatinase B (Matrix Metalloprotease-9), and Tissue Inhibitor of Metalloproteases-1 in the Basal Lamina Regulates Sertoli Cell-Tight Junction Dynamics in the Rat Testis. *Endocrinology* **2003**, *144*, 371–387, doi:10.1210/en.2002-220786.
9. Siu, M.K.Y.; Cheng, C.Y. Interactions of Proteases, Protease Inhibitors, and the Beta1Integrin/Laminin Gamma3 Protein Complex in the Regulation of Ectoplasmic Specialization Dynamics in the Rat Testis. *Biol. Reprod.* **2004**, *70*, 945–964, doi:10.1095/biolreprod.103.023606.
10. Siu, M.K.Y.; Cheng, C.Y. Extracellular Matrix and Its Role in Spermatogenesis. *Adv.Exp. Med. Biol.* **2008**, *636*, 74–91, doi:10.1007/978-0-387-09597-4_5.
11. Mohagheghi, S.; Khodadadi, I.; Karami, M.; Amiri, I.; Tavilani, H. Gene Polymorphism of Matrix Metalloproteinase 9 in Asthenozoospermic Male Subjects. *Int. J. Fertil. Steril.* **2018**, *11*, 247–252, doi:10.22074/ijfs.2018.5038.
12. Riaz, M.A.; Stammler, A.; Borgers, M.; Konrad, L. Clusterin Signals via ApoER2/VLDLR and Induces Meiosis of Male Germ Cells. *Am. J. Transl. Res.* **2017**,*9*, 1266–1276.
13. Tacken, P.J.; van der Zee, A.; Beumer, T.L.; Florijn, R.J.; Gijpels, M.J.; Havekes, L.M.; Frants, R.R.; van Dijk, K.W.; Hofker, M.H. Effective Generation of Very LowDensity Lipoprotein Receptor Transgenic Mice by

Overlapping Genomic DNA Fragments: High Testis Expression and Disturbed Spermatogenesis. *Transgenic Res.***2001**, *10*, 211–221, doi:10.1023/a:1016682520887.
14. Li, C.; Zhang, L.; Ma, T.; Gao, L.; Yang, L.; Wu, M.; Pang, Z.; Wang, X.; Yao, Q.; Xiao, Y.; et al. Bisphenol A Attenuates Testosterone Production in Leydig Cells via theInhibition of NR1D1 Signaling. *Chemosphere* **2021**, *263*, 128020, doi:10.1016/j.chemosphere.2020.128020.
15. Ferder, I.C.; Fung, L.; Ohguchi, Y.; Zhang, X.; Lassen, K.G.; Capen, D.; Brown, D.; Xavier, R.J.; Wang, N. Meiotic Gatekeeper STRA8 Suppresses Autophagy by Repressing Nr1d1 Expression during Spermatogenesis in Mice. *PLoS Genet.* **2019**, *15*,e1008084, doi:10.1371/journal.pgen.1008084.
16. Rappaport, M.S.; Smith, E.P. Insulin-like Growth Factor (IGF) Binding Protein 3 in theRat Testis: Follicle-Stimulating Hormone Dependence of MRNA Expression and Inhibition of IGF-I Action on Cultured Sertoli Cells. *Biol. Reprod.* **1995**, *52*, 419–425, doi:10.1095/biolreprod52.2.419.
17. Lin, T.; Wang, D.; Nagpal, M.L.; Shimasaki, S.; Ling, N. Expression and Regulation ofInsulin-like Growth Factor-Binding Protein-1, -2, -3, and -4 Messenger Ribonucleic Acids in Purified Rat Leydig Cells and Their Biological Effects. *Endocrinology* **1993**, *132*, 1898–1904, doi:10.1210/endo.132.5.7682935.
18. Lue, Y.; Swerdloff, R.; Liu, Q.; Mehta, H.; Hikim, A.S.; Lee, K.-W.; Jia, Y.; Hwang, D.; Cobb, L.J.; Cohen, P.; et al. Opposing Roles of Insulin-like Growth Factor BindingProtein 3 and Humanin in the Regulation of Testicular Germ Cell Apoptosis. *Endocrinology* **2010**, *151*, 350–357, doi:10.1210/en.2009-0577.
19. Toshima, J.; Ohashi, K.; Okano, I.; Nunoue, K.; Kishioka, M.; Kuma, K.; Miyata, T.; Hirai, M.; Baba, T.; Mizuno, K. Identification and Characterization of a Novel ProteinKinase, TESK1, Specifically Expressed in Testicular Germ Cells. *J. Biol. Chem.* **1995**,*270*, 31331–31337, doi:10.1074/jbc.270.52.31331.
20. Toshima, J.; Koji, T.; Mizuno, K. Stage-Specific Expression of Testis-Specific ProteinKinase 1 (TESK1) in Rat Spermatogenic Cells. *Biochem. Biophys. Res. Commun.* **1998**, *249*, 107–112, doi:10.1006/bbrc.1998.9099.
21. Toshima, J.; Toshima, J.Y.; Suzuki, M.; Noda, T.; Mizuno, K. Cell-Type-Specific Expression of a TESK1 Promoter-Linked LacZ Gene in Transgenic Mice. *Biochem. Biophys. Res. Commun.* **2001**, *286*, 566–573, doi:10.1006/bbrc.2001.5404.
22. Pati, D.; Meistrich, M.L.; Plon, S.E. Human Cdc34 and Rad6B Ubiquitin-ConjugatingEnzymes Target Repressors of Cyclic AMP-Induced Transcription for Proteolysis. *Mol. Cell. Biol.* **1999**, *19*, 5001–5013, doi:10.1128/mcb.19.7.5001.
23. Butz, N.; Ruetz, S.; Natt, F.; Hall, J.; Weiler, J.; Mestan, J.; Ducarre, M.; Grossenbacher, R.; Hauser, P.; Kempf, D.; et al. The Human Ubiquitin-Conjugating Enzyme Cdc34 Controls Cellular Proliferation through Regulation of P27Kip1 ProteinLevels. *Exp. Cell Res.* **2005**, *303*, 482–493, doi:10.1016/j.yexcr.2004.10.008.
24. Legesse-Miller, A.; Elemento, O.; Pfau, S.J.; Forman, J.J.; Tavazoie, S.; Coller, H.A. Let-7 Overexpression Leads to an Increased Fraction of Cells in G2/M, Direct down- Regulation of Cdc34, and Stabilization of Wee1 Kinase in Primary Fibroblasts. *J. Biol.Chem.* **2009**, *284*, 6605–6609, doi:10.1074/jbc.C900002200.
25. Hu, J.; You, S.; Li, W.; Wang, D.; Nagpal, M.L.; Mi, Y.; Liang, P.; Lin, T. Expressionand Regulation of Interferon-Gamma-Inducible Protein 10 Gene in Rat Leydig Cells. *Endocrinology* **1998**, *139*, 3637–3645, doi:10.1210/endo.139.8.6143.
26. Le Goffic, R.; Mouchel, T.; Aubry, F.; Patard, J.-J.; Ruffault, A.; Jégou, B.; Samson,
27. M. Production of the Chemokines Monocyte Chemotactic Protein-1, Regulated on Activation Normal T Cell Expressed and Secreted Protein, Growth-Related Oncogene,and Interferon-Gamma-Inducible Protein-10 Is Induced by the Sendai Virus in Humanand Rat Testicular Cells. *Endocrinology* **2002**, *143*, 1434–1440, doi:10.1210/endo.143.4.8735.
28. Aubry, F.; Habasque, C.; Satie, A.P.; Jégou, B.; Samson, M. Expression and Regulation of the CXC-Chemokines, GRO/KC and IP-10/Mob-1 in Rat SeminiferousTubules. *Eur. Cytokine Netw.* **2000**, *11*, 690–698.
29. Jiang, Q.; Wang, F.; Shi, L.; Zhao, X.; Gong, M.; Liu, W.; Song, C.; Li, Q.; Chen, Y.; Wu, H.; et al. C-X-C Motif Chemokine Ligand 10 Produced by Mouse Sertoli Cells inResponse to Mumps Virus Infection Induces Male Germ Cell Apoptosis. *Cell Death Dis.* **2017**, *8*, e3146, doi:10.1038/cddis.2017.560.
30. Dong, Y. -s; Hou, W. -g; Li, Y.; Liu, D. -b; Hao, G. -z; Zhang, H. -f; Li, J. -c; Zhao, J.;Zhang, S.; Liang, G. -b; et al. Unexpected Requirement for a Binding Partner of the Syntaxin Family in Phagocytosis by Murine Testicular Sertoli Cells. *Cell Death Differ.***2016**, *23*, 787–800, doi:10.1038/cdd.2015.139.
31. Merkl, M.; Ertl, R.; Handschuh, S.; Aurich, C.; Schäfer-Somi, S. The Cholesterol Transporter ABCA1 Is Expressed in Stallion Spermatozoa and Reproductive TractTissues. *Theriogenology* **2016**, *85*, 1080–1089, doi:10.1016/j.theriogenology.2015.11.019.
32. Stocchi, L.; Giardina, E.; Varriale, L.; Sechi, A.; Vagnini, A.; Parri, G.; Valentini, M.;Capalbo, M. Can Tangier Disease Cause Male Infertility? A Case Report and an Overview on Genetic Causes of Male Infertility and Hormonal Axis Involved. *Mol. Genet. Metab.* **2018**, *123*, 43–49, doi:10.1016/j.ymgme.2017.11.009.
33. Xia, Y.; Sidis, Y.; Schneyer, A. Overexpression of Follistatin-like 3 in Gonads CausesDefects in Gonadal Development and Function in Transgenic Mice. *Mol. Endocrinol. Baltim. Md* **2004**, *18*, 979–994, doi:10.1210/me.2003-0364.

34. Tsuchida, K.; Arai, K.Y.; Kuramoto, Y.; Yamakawa, N.; Hasegawa, Y.; Sugino, H.Identification and Characterization of a Novel Follistatin-like Protein as a Binding Protein for the TGF-Beta Family. *J. Biol. Chem.* **2000**, *275*, 40788–40796, doi:10.1074/jbc.M006114200.
35. Oldknow, K.J.; Seebacher, J.; Goswami, T.; Villen, J.; Pitsillides, A.A.; O'Shaughnessy, P.J.; Gygi, S.P.; Schneyer, A.L.; Mukherjee, A. Follistatin-like 3 (FSTL3) Mediated Silencing of Transforming Growth Factor β (TGFβ) Signaling Is Essential for Testicular Aging and Regulating Testis Size. *Endocrinology* **2013**, *154*, 1310–1320, doi:10.1210/en.2012-1886.
36. Hara-Yokoyama, M.; Kurihara, H.; Ichinose, S.; Matsuda, H.; Ichinose, S.; Kurosawa,M.; Tada, N.; Iwahara, C.; Terasawa, K.; Podyma-Inoue, K.A.; et al. KIF11 as a Potential Marker of Spermatogenesis Within Mouse Seminiferous Tubule Cross- Sections. *J. Histochem. Cytochem. Off. J. Histochem. Soc.* **2019**, *67*, 813–824, doi:10.1369/0022155419871027.
37. Blangy, A.; Lane, H.A.; d'Hérin, P.; Harper, M.; Kress, M.; Nigg, E.A. Phosphorylation by P34cdc2 Regulates Spindle Association of Human Eg5, a Kinesin-Related Motor Essential for Bipolar Spindle Formation in Vivo. *Cell* **1995**, *83*, 1159– 1169, doi:10.1016/0092-8674(95)90142-6.
38. Kapitein, L.C.; Peterman, E.J.G.; Kwok, B.H.; Kim, J.H.; Kapoor, T.M.; Schmidt, C.F.The Bipolar Mitotic Kinesin Eg5 Moves on Both Microtubules That It Crosslinks. *Nature* **2005**, *435*, 114–118, doi:10.1038/nature03503.
39. Ben-Eliezer, I.; Pomerantz, Y.; Galiani, D.; Nevo, N.; Dekel, N. Appropriate Expression of Ube2C and Ube2S Controls the Progression of the First MeioticDivision. *FASEB J.* **2015**, *29*, 4670–4681, doi:10.1096/fj.15-274522.
40. Peters, J.-M. The Anaphase-Promoting Complex: Proteolysis in Mitosis and Beyond.
41. *Mol. Cell* **2002**, *9*, 931–943, doi:10.1016/s1097-2765(02)00540-3.
42. Hao, Z.; Zhang, H.; Cowell, J. Ubiquitin-Conjugating Enzyme UBE2C: Molecular Biology, Role in Tumorigenesis, and Potential as a Biomarker. *Tumour Biol. J. Int. Soc. Oncodevelopmental Biol. Med.* **2012**, *33*, 723–730, doi:10.1007/s13277-011-0291-1.
43. Boyer, A.; Yeh, J.R.; Zhang, X.; Paquet, M.; Gaudin, A.; Nagano, M.C.; Boerboom, D.CTNNB1 Signaling in Sertoli Cells Downregulates Spermatogonial Stem Cell Activityvia WNT4. *PloS One* **2012**, *7*, e29764, doi:10.1371/journal.pone.0029764.
44. Jordan, B.K.; Shen, J.H.-C.; Olaso, R.; Ingraham, H.A.; Vilain, E. Wnt4 Overexpression Disrupts Normal Testicular Vasculature and Inhibits Testosterone Synthesis by Repressing Steroidogenic Factor 1/Beta-Catenin Synergy. *Proc. Natl.Acad. Sci. U. S. A.* **2003**, *100*, 10866–10871, doi:10.1073/pnas.1834480100.
45. Adams, R.R.; Eckley, D.M.; Vagnarelli, P.; Wheatley, S.P.; Gerloff, D.L.; Mackay, A.M.; Svingen, P.A.; Kaufmann, S.H.; Earnshaw, W.C. Human INCENP Colocalizeswith the Aurora-B/AIRK2 Kinase on Chromosomes and Is Overexpressed in TumourCells. *Chromosoma* **2001**, *110*, 65–74, doi:10.1007/s004120100130.
46. Parra, M.T.; Viera, A.; Gómez, R.; Page, J.; Carmena, M.; Earnshaw, W.C.; Rufas,J.S.; Suja, J.A. Dynamic Relocalization of the Chromosomal Passenger Complex Proteins Inner Centromere Protein (INCENP) and Aurora-B Kinase during Male Mouse Meiosis. *J. Cell Sci.* **2003**, *116*, 961, doi:10.1242/jcs.00330.
47. Goto, H.; Kiyono, T.; Tomono, Y.; Kawajiri, A.; Urano, T.; Furukawa, K.; Nigg, E.A.;Inagaki, M. Complex Formation of Plk1 and INCENP Required for Metaphase- Anaphase Transition. *Nat. Cell Biol.* **2006**, *8*, 180–187, doi:10.1038/ncb1350.
48. Matsubara, N.; Yanagisawa, M.; Nishimune, Y.; Obinata, M.; Matsui, Y. Murine Pololike Kinase 1 Gene Is Expressed in Meiotic Testicular Germ Cells and Oocytes. *Mol. Reprod. Dev.* **1995**, *41*, 407–415, doi:10.1002/mrd.1080410403.
49. Jordan, P.W.; Karppinen, J.; Handel, M.A. Polo-like Kinase Is Required for Synaptonemal Complex Disassembly and Phosphorylation in Mouse Spermatocytes. *J.Cell Sci.* **2012**, *125*, 5061, doi:10.1242/jcs.105015.
50. Liu, D.; Davydenko, O.; Lampson, M.A. Polo-like Kinase-1 Regulates Kinetochore- Microtubule Dynamics and Spindle Checkpoint Silencing. *J. Cell Biol.* **2012**, *198*, 491–499, doi:10.1083/jcb.201205090.
51. Mondal, G.; Ohashi, A.; Yang, L.; Rowley, M.; Couch, F.J. Tex14, a Plk1-RegulatedProtein, Is Required for Kinetochore-Microtubule Attachment and Regulation of the Spindle Assembly Checkpoint. *Mol. Cell* **2012**, *45*, 680–695, doi:10.1016/j.molcel.2012.01.013.
52. Jordan, P.W.; Karppinen, J.; Handel, M.A. Polo-like Kinase Is Required for Synaptonemal Complex Disassembly and Phosphorylation in Mouse Spermatocytes. *J. Cell Sci.* **2012**, *125*, 5061–5072, doi:10.1242/jcs.105015.
53. Giménez-Abián, J.F.; Sumara, I.; Hirota, T.; Hauf, S.; Gerlich, D.; de la Torre, C.;Ellenberg, J.; Peters, J.-M. Regulation of Sister Chromatid Cohesion between Chromosome Arms. *Curr. Biol. CB* **2004**, *14*, 1187–1193, doi:10.1016/j.cub.2004.06.052.
54. Matsumura, S.; Toyoshima, F.; Nishida, E. Polo-like Kinase 1 Facilitates ChromosomeAlignment during Prometaphase through BubR1. *J. Biol. Chem.* **2007**, *282*, 15217– 15227, doi:10.1074/jbc.M611053200.
55. Kong, M.; Barnes, E.A.; Ollendorff, V.; Donoghue, D.J. Cyclin F Regulates the Nuclear Localization of Cyclin B1 through a Cyclin-Cyclin Interaction. *EMBO J.* **2000**,*19*, 1378–1388, doi:10.1093/emboj/19.6.1378.

56. Sun, Y.; Fu, L.; Xue, F.; Li, Y.; Xu, H.; Chen, J. Digital Gene Expression Profiling andValidation Study Highlight Cyclin F as an Important Regulator for Sperm Motility of Chickens. *Poult. Sci.* **2019**, *98*, 5118–5126, doi:10.3382/ps/pez212.
57. Orwig, K.E.; Ryu, B.-Y.; Master, S.R.; Phillips, B.T.; Mack, M.; Avarbock, M.R.; Chodosh, L.; Brinster, R.L. Genes Involved in Post-Transcriptional Regulation Are Overrepresented in Stem/Progenitor Spermatogonia of Cryptorchid Mouse Testes. *StemCells Dayt. Ohio* **2008**, *26*, 927–938, doi:10.1634/stemcells.2007-0893.
58. Park, H.-J.; Lee, W.-Y.; Park, C.; Hong, K.; Song, H. CD14 Is a Unique Membrane Marker of Porcine Spermatogonial Stem Cells, Regulating Their Differentiation. *Sci.Rep.* **2019**, *9*, 9980, doi:10.1038/s41598-019-46000-6.
59. Hu, J.; Zhang, Y.-Q.; Liu, X.-P.; Wang, R.-A.; Jin, Y.; Xu, R.-J. Expression and Localization of Smad1, Smad2 and Smad4 Proteins in Rat Testis during PostnatalDevelopment. *Asian J. Androl.* **2003**, *5*, 51–55.
60. Palladino, M.A.; Fasano, G.A.; Patel, D.; Dugan, C.; London, M. Effects of Lipopolysaccharide-Induced Inflammation on Hypoxia and Inflammatory GeneExpression Pathways of the Rat Testis. *Basic Clin. Androl.* **2018**, *28*, 14, doi:10.1186/s12610-018-0079-x.
61. Winnall, W.R.; Hedger, M.P. Phenotypic and Functional Heterogeneity of the Testicular Macrophage Population: A New Regulatory Model. *J. Reprod. Immunol.***2013**, *97*, 147–158, doi:10.1016/j.jri.2013.01.001.
62. Rother, K.; Dengl, M.; Lorenz, J.; Tschöp, K.; Kirschner, R.; Mössner, J.; Engeland, K.Gene Expression of Cyclin-Dependent Kinase Subunit Cks2 Is Repressed by the Tumor Suppressor P53 but Not by the Related Proteins P63 or P73. *FEBS Lett.* **2007**, *581*, 1166–1172, doi:10.1016/j.febslet.2007.02.028.
63. Spruck, C.H.; de Miguel, M.P.; Smith, A.P.L.; Ryan, A.; Stein, P.; Schultz, R.M.;Lincoln, A.J.; Donovan, P.J.; Reed, S.I. Requirement of Cks2 for the First Metaphase/Anaphase Transition of Mammalian Meiosis. *Science* **2003**, *300*, 647,doi:10.1126/science.1084149.
64. Smirnova, N.A.; Romanienko, P.J.; Khil, P.P.; Camerini-Otero, R.D. Gene ExpressionProfiles of Spo11-/- Mouse Testes with Spermatocytes Arrested in Meiotic Prophase I.*Reprod. Camb. Engl.* **2006**, *132*, 67–77, doi:10.1530/rep.1.00997.
65. Kominami, E.; Tsukahara, T.; Bando, Y.; Katunuma, N. Distribution of Cathepsins B and H in Rat Tissues and Peripheral Blood Cells. *J. Biochem. (Tokyo)* **1985**, *98*, 87–93,doi:10.1093/oxfordjournals.jbchem.a135277.
66. Haraguchi, C.M.; Ishido, K.; Kominami, E.; Yokota, S. Expression of Cathepsin H inDifferentiating Rat Spermatids: Immunoelectron Microscopic Study. *Histochem. CellBiol.* **2003**, *120*, 63–71, doi:10.1007/s00418-003-0545-0.
67. Murphy, K.; Carvajal, L.; Medico, L.; Pepling, M. Expression of Stat3 in Germ Cells ofDeveloping and Adult Mouse Ovaries and Testes. *Gene Expr. Patterns GEP* **2005**, *5*, 475–482, doi:10.1016/j.modgep.2004.12.007.
68. Nagasawa, K.; Imura-Kishi, K.; Uchida, A.; Hiramatsu, R.; Kurohmaru, M.; Kanai, Y.Regionally Distinct Patterns of STAT3 Phosphorylation in the Seminiferous Epithelia of Mouse Testes. *Mol. Reprod. Dev.* **2018**, *85*, 262–270, doi:10.1002/mrd.22962.
69. Saxena, N.K.; Vertino, P.M.; Anania, F.A.; Sharma, D. Leptin-Induced Growth Stimulation of Breast Cancer Cells Involves Recruitment of Histone Acetyltransferasesand Mediator Complex to CYCLIN D1 Promoter via Activation of Stat3. *J. Biol. Chem.* **2007**, *282*, 13316–13325, doi:10.1074/jbc.M609798200.
70. Oatley, J.M.; Brinster, R.L. The Germline Stem Cell Niche Unit in Mammalian Testes.
71. *Physiol. Rev.* **2012**, *92*, 577–595, doi:10.1152/physrev.00025.2011.
72. Sisakhtnezhad, S.; Heshmati, P. Comparative Analysis of Single-Cell RNA SequencingData from Mouse Spermatogonial and Mesenchymal Stem Cells to Identify Differentially Expressed Genes and Transcriptional Regulators of Germline Cells. *J. Cell. Physiol.* **2018**, *233*, 5231–5242, doi:10.1002/jcp.26303.
73. Chen, J.-X.; Xu, L.-L.; Wang, X.-C.; Qin, H.-Y.; Wang, J.-L. Involvement of C- Src/STAT3 Signal in EGF-Induced Proliferation of Rat Spermatogonial Stem Cells. *Mol. Cell. Biochem.* **2011**, *358*, 67–73, doi:10.1007/s11010-011-0922-2.
74. Haraguchi, S.; Ikeda, M.; Akagi, S.; Hirao, Y. Dynamic Changes in PStat3 Are Involved in Meiotic Spindle Assembly in Mouse Oocytes. *Int. J. Mol. Sci.* **2020**, *21*,E1220, doi:10.3390/ijms21041220.
75. Habasque, C.; Satie, A.-P.; Aubry, F.; Jégou, B.; Samson, M. Expression of Fractalkinein the Rat Testis: Molecular Cloning of a Novel Alternative Transcript of Its Gene ThatIs Differentially Regulated by pro-Inflammatory Cytokines. *Mol. Hum. Reprod.* **2003**, *9*, 449–455, doi:10.1093/molehr/gag059.
76. Truman, L.A.; Ford, C.A.; Pasikowska, M.; Pound, J.D.; Wilkinson, S.J.; Dumitriu, I.E.; Melville, L.; Melrose, L.A.; Ogden, C.A.; Nibbs, R.; et al. CX3CL1/Fractalkine IsReleased from Apoptotic Lymphocytes to Stimulate Macrophage Chemotaxis. *Blood* **2008**, *112*, 5026–5036, doi:10.1182/blood-2008-06-162404.
77. Fischer, K.A.; Van Leyen, K.; Lovercamp, K.W.; Manandhar, G.; Sutovsky, M.; Feng,D.; Safranski, T.; Sutovsky, P. 15-Lipoxygenase Is a Component of the Mammalian Sperm Cytoplasmic Droplet. *Reprod. Camb. Engl.* **2005**, *130*, 213–222, doi:10.1530/rep.1.00646.

78. Bromfield, E.G.; Mihalas, B.P.; Dun, M.D.; Aitken, R.J.; McLaughlin, E.A.; Walters, J.L.H.; Nixon, B. Inhibition of Arachidonate 15-Lipoxygenase Prevents 4- Hydroxynonenal-Induced Protein Damage in Male Germ Cells. *Biol. Reprod.* **2017**, *96*,598–609, doi:10.1093/biolre/iox005.
79. Bromfield, E.G.; Walters, J.L.H.; Cafe, S.L.; Bernstein, I.R.; Stanger, S.J.; Anderson,A.L.; Aitken, R.J.; McLaughlin, E.A.; Dun, M.D.; Gadella, B.M.; et al. Differential Cell Death Decisions in the Testis: Evidence for an Exclusive Window of Ferroptosisin Round Spermatids. *Mol. Hum. Reprod.* **2019**, *25*, 241–256, doi:10.1093/molehr/gaz015.
80. Walters, J.L.H.; De Iuliis, G.N.; Dun, M.D.; Aitken, R.J.; McLaughlin, E.A.; Nixon,B.; Bromfield, E.G. Pharmacological Inhibition of Arachidonate 15-Lipoxygenase Protects Human Spermatozoa against Oxidative Stress. *Biol. Reprod.* **2018**, *98*, 784–794, doi:10.1093/biolre/ioy058.
81. Izumi, H.; Matsumoto, Y.; Ikeuchi, T.; Saya, H.; Kajii, T.; Matsuura, S. BubR1 Localizes to Centrosomes and Suppresses Centrosome Amplification via RegulatingPlk1 Activity in Interphase Cells. *Oncogene* **2009**, *28*, 2806–2820, doi:10.1038/onc.2009.141.
82. Jeganathan, K.B.; van Deursen, J.M. Differential Mitotic Checkpoint Protein Requirements in Somatic and Germ Cells. *Biochem. Soc. Trans.* **2006**, *34*, 583–586,doi:10.1042/BST0340583.
83. Choi, E.; Lee, H. Chromosome Damage in Mitosis Induces BubR1 Activation andPrometaphase Arrest. *FEBS Lett.* **2008**, *582*, 1700–1706, doi:10.1016/j.febslet.2008.04.028.
84. Parra, M.T.; Gómez, R.; Viera, A.; Llano, E.; Pendás, A.M.; Rufas, J.S.; Suja, J.A. Sequential Assembly of Centromeric Proteins in Male Mouse Meiosis. *PLoS Genet.***2009**, *5*, e1000417–e1000417, doi:10.1371/journal.pgen.1000417.
85. Ravnik, S.E.; Wolgemuth, D.J. The Developmentally Restricted Pattern of Expressionin the Male Germ Line of a Murine Cyclin A, Cyclin A2, Suggests Roles in Both Mitotic and Meiotic Cell Cycles. *Dev. Biol.* **1996**, *173*, 69–78, doi:10.1006/dbio.1996.0007.
86. Ravnik, S.E.; Wolgemuth, D.J. Regulation of Meiosis during Mammalian Spermatogenesis: The A-Type Cyclins and Their Associated Cyclin-Dependent Kinases Are Differentially Expressed in the Germ-Cell Lineage. *Dev. Biol.* **1999**, *207*,408–418, doi:10.1006/dbio.1998.9156.
87. Sweeney, C.; Murphy, M.; Kubelka, M.; Ravnik, S.E.; Hawkins, C.F.; Wolgemuth,D.J.; Carrington, M. A Distinct Cyclin A Is Expressed in Germ Cells in the Mouse.*Dev. Camb. Engl.* **1996**, *122*, 53–64.
88. Ma, F.; Wang, X.; Chung, S.S.W.; Sicinski, P.; Shang, E.; Wolgemuth, D.J. Cyclin A2Is Essential for Mouse Gonocyte Maturation. *Cell Cycle Georget. Tex* **2020**, *19*, 1654–1664, doi:10.1080/15384101.2020.1762314.
89. Zhang, Q.-H.; Yuen, W.S.; Adhikari, D.; Flegg, J.A.; FitzHarris, G.; Conti, M.; Sicinski, P.; Nabti, I.; Marangos, P.; Carroll, J. Cyclin A2 Modulates Kinetochore-Microtubule Attachment in Meiosis II. *J. Cell Biol.* **2017**, *216*, 3133–3143, doi:10.1083/jcb.201607111.
90. Ergün, S.; Kiliç, N.; Fiedler, W.; Mukhopadhyay, A.K. Vascular Endothelial GrowthFactor and Its Receptors in Normal Human Testicular Tissue. *Mol. Cell. Endocrinol.***1997**, *131*, 9–20, doi:10.1016/s0303-7207(97)00082-8.
91. Bott, R.C.; Clopton, D.T.; Fuller, A.M.; McFee, R.M.; Lu, N.; McFee, R.M.; Cupp,
92. A.S. KDR-LacZ-Expressing Cells Are Involved in Ovarian and Testis-Specific Vascular Development, Suggesting a Role for VEGFA in the Regulation of This Vasculature. *Cell Tissue Res.* **2010**, *342*, 117–130, doi:10.1007/s00441-010-1038-9.
93. Caires, K.C.; de Avila, J.; McLean, D.J. Vascular Endothelial Growth Factor RegulatesGerm Cell Survival during Establishment of Spermatogenesis in the Bovine Testis. *Reprod. Camb. Engl.* **2009**, *138*, 667–677, doi:10.1530/REP-09-0020.
94. Tian, R.; Yang, S.; Zhu, Y.; Zou, S.; Li, P.; Wang, J.; Zhu, Z.; Huang, Y.; He, Z.; Li, Z.VEGF/VEGFR2 Signaling Regulates Germ Cell Proliferation in Vitro and Promotes Mouse Testicular Regeneration in Vivo. *Cells Tissues Organs* **2016**, *201*, 1–13, doi:10.1159/000440949.
95. Fon Tacer, K.; Kalanj-Bognar, S.; Waterman, M.R.; Rozman, D. Lanosterol Metabolism and Sterol Regulatory Element Binding Protein (SREBP) Expression inMale Germ Cell Maturation. *J. Steroid Biochem. Mol. Biol.* **2003**, *85*, 429–438, doi:10.1016/s0960-0760(03)00191-2.
96. Brown, M.S.; Goldstein, J.L. A Proteolytic Pathway That Controls the CholesterolContent of Membranes, Cells, and Blood. *Proc. Natl. Acad. Sci. U. S. A.* **1999**, *96*,11041–11048, doi:10.1073/pnas.96.20.11041.
97. Osborne, T.F. Sterol Regulatory Element-Binding Proteins (SREBPs): Key Regulatorsof Nutritional Homeostasis and Insulin Action. *J. Biol. Chem.* **2000**, *275*, 32379– 32382, doi:10.1074/jbc.R000017200.
98. Vannelli, B.G.; Orlando, C.; Barni, T.; Natali, A.; Serio, M.; Balboni, G.C. Immunostaining of Transferrin and Transferrin Receptor in Human SeminiferousTubules. *Fertil. Steril.* **1986**, *45*, 536–541, doi:10.1016/s0015-0282(16)49284-8.
99. Forti, G.; Barni, T.; Vannelli, B.G.; Balboni, G.C.; Orlando, C.; Serio, M. Sertoli CellProteins in the Human Seminiferous Tubule. *J. Steroid Biochem.* **1989**, *32*, 135–144, doi:10.1016/0022-4731(89)90154-4.

100. Petrie, R.G.; Morales, C.R. Receptor-Mediated Endocytosis of Testicular Transferrinby Germinal Cells of the Rat Testis. *Cell Tissue Res.* **1992**, *267*, 45–55, doi:10.1007/BF00318690.
101. Bhattacharya, I.; Basu, S.; Pradhan, B.S.; Sarkar, H.; Nagarajan, P.; Majumdar, S.S. Testosterone Augments FSH Signaling by Upregulating the Expression and Activity ofFSH-Receptor in Pubertal Primate Sertoli Cells. *Mol. Cell. Endocrinol.* **2019**, *482*, 70–80, doi:10.1016/j.mce.2018.12.012.
102. Lécureuil, C.; Staub, C.; Fouchécourt, S.; Maurel, M.C.; Fontaine, I.; Martinat, N.; Gauthier, C.; Daudignon, A.; Delaleu, B.; Sow, A.; et al. Transferrin OverexpressionAlters Testicular Function in Aged Mice. *Mol. Reprod. Dev.* **2007**, *74*, 197–206, doi:10.1002/mrd.20523.
103. Baumann, C.; De La Fuente, R. Role of Polycomb Group Protein Cbx2/M33 in MeiosisOnset and Maintenance of Chromosome Stability in the Mammalian Germline. *Genes* **2011**, *2*, 59–80, doi:10.3390/genes2010059.
104. Yang, Q.-E.; Kim, D.; Kaucher, A.; Oatley, M.J.; Oatley, J.M. CXCL12-CXCR4 Signaling Is Required for the Maintenance of Mouse Spermatogonial Stem Cells. *J.Cell Sci.* **2013**, *126*, 1009–1020, doi:10.1242/jcs.119826.
105. Westernströer, B.; Terwort, N.; Ehmcke, J.; Wistuba, J.; Schlatt, S.; Neuhaus, N. Profiling of Cxcl12 Receptors, Cxcr4 and Cxcr7 in Murine Testis Development and aSpermatogenic Depletion Model Indicates a Role for Cxcr7 in Controlling Cxcl12 Activity. *PloS One* **2014**, *9*, e112598, doi:10.1371/journal.pone.0112598.
106. Habasque, C.; Aubry, F.; Jégou, B.; Samson, M. Study of the HIV-1 Receptors CD4,CXCR4, CCR5 and CCR3 in the Human and Rat Testis. *Mol. Hum. Reprod.* **2002**, *8*,419–425, doi:10.1093/molehr/8.5.419.
107. Goissis, M.D.; Giassetti, M.I.; Worst, R.A.; Mendes, C.M.; Moreira, P.V.; Assumpção,M.E.O.A.; Visintin, J.A. Spermatogonial Stem Cell Potential of CXCR4-Positive Cellsfrom Prepubertal Bull Testes. *Anim. Reprod. Sci.* **2018**, *196*, 219–229, doi:10.1016/j.anireprosci.2018.08.014.
108. Molyneaux, K.A.; Zinszner, H.; Kunwar, P.S.; Schaible, K.; Stebler, J.; Sunshine, M.J.;O'Brien, W.; Raz, E.; Littman, D.; Wylie, C.; et al. The Chemokine SDF1/CXCL12 and Its Receptor CXCR4 Regulate Mouse Germ Cell Migration and Survival. *Dev. Camb. Engl.* **2003**, *130*, 4279–4286, doi:10.1242/dev.00640.
109. Li, H.; Liang, R.; Lu, Y.; Wang, M.; Li, Z. RTN3 Regulates the Expression Level of Chemokine Receptor CXCR4 and Is Required for Migration of Primordial Germ Cells. *Int. J. Mol. Sci.* **2016**, *17*, 382, doi:10.3390/ijms17040382.
110. Lee, J.H.; Park, J.-W.; Kim, S.W.; Park, J.; Park, T.S. C-X-C Chemokine Receptor Type 4 (CXCR4) Is a Key Receptor for Chicken Primordial Germ Cell Migration. *J. Reprod. Dev.* **2017**, *63*, 555–562, doi:10.1262/jrd.2017-067.
111. Zhang, Y.; Luo, F.; Wu, S.; Yu, B.; Liu, T.; Wu, Y. Tribbles Homolog 3 Expression inSpermatogonial Stem Cells of Rat Testes. *Cell Biol. Int.* **2014**, *38*, 1403–1407, doi:10.1002/cbin.10338.
112. Zhong, X.; Liu, L.; Zhao, A.; Pfeifer, G.P.; Xu, X. The Abnormal Spindle-like, Microcephaly-Associated (ASPM) Gene Encodes a Centrosomal Protein. *Cell Cycle Georget. Tex* **2005**, *4*, 1227–1229, doi:10.4161/cc.4.9.2029.
113. Pulvers, J.N.; Bryk, J.; Fish, J.L.; Wilsch-Bräuninger, M.; Arai, Y.; Schreier, D.;Naumann, R.; Helppi, J.; Habermann, B.; Vogt, J.; et al. Mutations in Mouse
114. <em>Aspm</Em> (Abnormal Spindle-like Microcephaly Associated) Cause Not OnlyMicrocephaly but Also Major Defects in the Germline. *Proc. Natl. Acad. Sci.* **2010**, *107*, 16595, doi:10.1073/pnas.1010494107.
115. Coultas, L.; Bouillet, P.; Loveland, K.L.; Meachem, S.; Perlman, H.; Adams, J.M.; Strasser, A. Concomitant Loss of Proapoptotic BH3-Only Bcl-2 Antagonists Bik andBim Arrests Spermatogenesis. *EMBO J.* **2005**, *24*, 3963–3973, doi:10.1038/sj.emboj.7600857.
116. Guazzone, V.A.; Rival, C.; Denduchis, B.; Lustig, L. Monocyte Chemoattractant Protein-1 (MCP-1/CCL2) in Experimental Autoimmune Orchitis. *J. Reprod. Immunol.***2003**, *60*, 143–157, doi:10.1016/j.jri.2003.08.001.
117. Aubry, F.; Habasque, C.; Satie, A.P.; Jégou, B.; Samson, M. Expression and Regulation of the CC-Chemokine Monocyte Chemoattractant Protein-1 in RatTesticular Cells in Primary Culture. *Biol. Reprod.* **2000**, *62*, 1427–1435, doi:10.1095/biolreprod62.5.1427.
118. Guazzone, V.A.; Rival, C.; Denduchis, B.; Lustig, L. Monocyte Chemoattractant Protein-1 (MCP-1/CCL2) in Experimental Autoimmune Orchitis. *J. Reprod. Immunol.***2003**, *60*, 143–157, doi:10.1016/j.jri.2003.08.001.
119. Guazzone, V.A.; Jacobo, P.; Theas, M.S.; Lustig, L. Cytokines and Chemokines inTesticular Inflammation: A Brief Review. *Microsc. Res. Tech.* **2009**, *72*, 620–628,doi:10.1002/jemt.20704.
120. Vagnarelli, P.; Ribeiro, S.; Sennels, L.; Sanchez-Pulido, L.; de Lima Alves, F.; Verheyen, T.; Kelly, D.A.; Ponting, C.P.; Rappsilber, J.; Earnshaw, W.C. Repo-Man Coordinates Chromosomal Reorganization with Nuclear Envelope Reassembly during Mitotic Exit. *Dev. Cell* **2011**, *21*, 328–342, doi:10.1016/j.devcel.2011.06.020.
121. Wurzenberger, C.; Held, M.; Lampson, M.A.; Poser, I.; Hyman, A.A.; Gerlich, D.W. Sds22 and Repo-Man Stabilize Chromosome Segregation by Counteracting Aurora Bon Anaphase Kinetochores. *J. Cell Biol.* **2012**, *198*, 173–183, doi:10.1083/jcb.201112112.

122. Vagnarelli, P.; Hudson, D.F.; Ribeiro, S.A.; Trinkle-Mulcahy, L.; Spence, J.M.; Lai, F.;Farr, C.J.; Lamond, A.I.; Earnshaw, W.C. Condensin and Repo-Man-PP1 Co-Operate in the Regulation of Chromosome Architecture during Mitosis. *Nat. Cell Biol.* **2006**, *8*, 1133–1142, doi:10.1038/ncb1475.
123. Tomes, C.N. The Proteins of Exocytosis: Lessons from the Sperm Model. *Biochem. J.*
124. **2015**, *465*, 359–370, doi:10.1042/BJ20141169.
125. Itzhakov, D.; Nitzan, Y.; Breitbart, H. Protein Kinase A Inhibition Induces EPAC- Dependent Acrosomal Exocytosis in Human Sperm. *Asian J. Androl.* **2019**, *21*, 337–344, doi:10.4103/aja.aja_99_18.
126. Rajender, S.; Avery, K.; Agarwal, A. Epigenetics, Spermatogenesis and MaleInfertility. *Mutat. Res.* **2011**, *727*, 62–71, doi:10.1016/j.mrrev.2011.04.002.
127. Tang, Q.; Pan, F.; Yang, J.; Fu, Z.; Lu, Y.; Wu, X.; Han, X.; Chen, M.; Lu, C.; Xia, Y.;et al. Idiopathic Male Infertility Is Strongly Associated with Aberrant DNA Methylation of Imprinted Loci in Sperm: A Case-Control Study. *Clin. Epigenetics* **2018**, *10*, 134, doi:10.1186/s13148-018-0568-y.
128. Griffeth, R.J.; Bianda, V.; Nef, S. The Emerging Role of Insulin-like Growth Factors inTestis Development and Function. *Basic Clin. Androl.* **2014**, *24*, 12, doi:10.1186/2051-4190-24-12.
129. Söder, O.; Bang, P.; Wahab, A.; Parvinen, M. Insulin-like Growth Factors SelectivelyStimulate Spermatogonial, but Not Meiotic, Deoxyribonucleic Acid Synthesis during Rat Spermatogenesis. *Endocrinology* **1992**, *131*, 2344–2350, doi:10.1210/endo.131.5.1425434.
130. Chan, Y.-F.; O, W.-S.; Tang, F. Adrenomedullin in the Rat Testis. I: Its Production, Actions on Testosterone Secretion, Regulation by Human Chorionic Gonadotropin, andIts Interaction with Endothelin 1 in the Leydig Cell. *Biol. Reprod.* **2008**, *78*, 773–779, doi:10.1095/biolreprod.107.060871.
131. Chan, Y.-F.; Tang, F.; O, W.-S. Adrenomedullin in the Rat Testis. II: Its Production,Actions on Inhibin Secretion, Regulation by Follicle-Stimulating Hormone, and Its Interaction with Endothelin 1 in the Sertoli Cell. *Biol. Reprod.* **2008**, *78*, 780–785, doi:10.1095/biolreprod.107.060863.
132. Kelly, M.; Widjaja-Adhi, M.A.K.; Palczewski, G.; von Lintig, J. Transport of Vitamin A across Blood-Tissue Barriers Is Facilitated by STRA6. *FASEB J. Off. Publ. Fed. Am. Soc. Exp. Biol.* **2016**, *30*, 2985–2995, doi:10.1096/fj.201600446R.
133. Chapman, D.L.; Wolgemuth, D.J. Isolation of the Murine Cyclin B2 CDNA and Characterization of the Lineage and Temporal Specificity of Expression of the B1 and B2 Cyclins during Oogenesis, Spermatogenesis and Early Embryogenesis. *Dev. Camb.Engl.* **1993**, *118*, 229–240.
134. Brandeis, M.; Rosewell, I.; Carrington, M.; Crompton, T.; Jacobs, M.A.; Kirk, J.; Gannon, J.; Hunt, T. Cyclin B2-Null Mice Develop Normally and Are Fertile WhereasCyclin B1-Null Mice Die in Utero. *Proc. Natl. Acad. Sci. U. S. A.* **1998**, *95*, 4344– 4349, doi:10.1073/pnas.95.8.4344.
135. Chotiner, J.Y.; Wolgemuth, D.J.; Wang, P.J. Functions of Cyclins and CDKs inMammalian Gametogenesis†. *Biol. Reprod.* **2019**, doi:10.1093/biolre/ioz070.
136. Daldello, E.M.; Luong, X.G.; Yang, C.-R.; Kuhn, J.; Conti, M. Cyclin B2 Is Requiredfor Progression through Meiosis in Mouse Oocytes. *Dev. Camb. Engl.* **2019**, *146*, doi:10.1242/dev.172734.
137. Hsia, N.; Cornwall, G.A. Cres2 and Cres3: New Members of the Cystatin-Related Epididymal Spermatogenic Subgroup of Family 2 Cystatins. *Endocrinology* **2003**, *144*,909–915, doi:10.1210/en.2002-220890.
138. Li, Y.; Putnam-Lawson, C.A.; Knapp-Hoch, H.; Friel, P.J.; Mitchell, D.; Hively, R.; Griswold, M.D. Immunolocalization and Regulation of Cystatin 12 in Mouse Testis and Epididymis. *Biol. Reprod.* **2005**, *73*, 872–880, doi:10.1095/biolreprod.105.040238.
139. Whelly, S.; Muthusubramanian, A.; Powell, J.; Johnson, S.; Hastert, M.C.; Cornwall,
140. G.A. Cystatin-Related Epididymal Spermatogenic Subgroup Members Are Part of anAmyloid Matrix and Associated with Extracellular Vesicles in the Mouse EpididymalLumen. *Mol. Hum. Reprod.* **2016**, *22*, 729–744, doi:10.1093/molehr/gaw049.
141. Lin, H.; Yuan, K.; Zhou, H.; Bu, T.; Su, H.; Liu, S.; Zhu, Q.; Wang, Y.; Hu, Y.; Shan, Y.; et al. Time-Course Changes of Steroidogenic Gene Expression and Steroidogenesisof Rat Leydig Cells after Acute Immobilization Stress. *Int. J. Mol. Sci.* **2014**, *15*, 21028–21044, doi:10.3390/ijms151121028.
142. Mruk, D.D.; Cheng, C.Y. The Mammalian Blood-Testis Barrier: Its Biology andRegulation. *Endocr. Rev.* **2015**, *36*, 564–591, doi:10.1210/er.2014-1101.
143. Cai, Y.; Liu, T.; Fang, F.; Shen, S.; Xiong, C. Involvement of ICAM-1 in ImpairedSpermatogenesis after Busulfan Treatment in Mice. *Andrologia* **2016**, *48*, 37–44, doi:10.1111/and.12414.
144. Xiao, X.; Cheng, C.Y.; Mruk, D.D. Intercellular Adhesion Molecule-1 Is a Regulator ofBlood-Testis Barrier Function. *J. Cell Sci.* **2012**, *125*, 5677–5689, doi:10.1242/jcs.107987.
145. Bakshi, R.P.; Galande, S.; Bali, P.; Dighe, R.; Muniyappa, K. Developmental andHormonal Regulation of Type II DNA Topoisomerase in Rat Testis. *J. Mol. Endocrinol.* **2001**, *26*, 193–206.
146. Chen, J.L.; Guo, S.H.; Gao, F.H. Nuclear Matrix in Developing Rat SpermatogenicCells. *Mol. Reprod. Dev.* **2001**, *59*, 314–321, doi:10.1002/mrd.1036.

147. Roca, J.; Mezquita, C. DNA Topoisomerase II Activity in Nonreplicating, Transcriptionally Inactive, Chicken Late Spermatids. *EMBO J.* **1989**, *8*, 1855–1860.
148. Bakshi, R.; Galande, S.; Muniyappa, K. Substrate Specificity Plays an Important Rolein Uncoupling the Catalytic and Scaffolding Activities of Rat Testis DNA Topoisomerase IIalpha. *J. Biomol. Struct. Dyn.* **2001**, *18*, 749–760, doi:10.1080/07391102.2001.10506704.
149. Sukhacheva, T.V.; Bogush, T.A.; Kolomiets, O.L. Destructive Effect of DNA Topoisomerase II Inhibitor Vepesid on Mouse Spermatogenesis. *Bull. Exp. Biol. Med.***2003**, *135*, 464–469, doi:10.1023/a:1024919510096.
150. Payne, A.H.; Hales, D.B. Overview of Steroidogenic Enzymes in the Pathway fromCholesterol to Active Steroid Hormones. *Endocr. Rev.* **2004**, *25*, 947–970, doi:10.1210/er.2003-0030.
151. Zirkin, B.R.; Papadopoulos, V. Leydig Cells: Formation, Function, and Regulation.
152. *Biol. Reprod.* **2018**, *99*, 101–111, doi:10.1093/biolre/ioy059.
153. Miller, W.L.; Auchus, R.J. The Molecular Biology, Biochemistry, and Physiology ofHuman Steroidogenesis and Its Disorders. *Endocr. Rev.* **2011**, *32*, 81–151, doi:10.1210/er.2010-0013.
154. Iwamori, T.; Iwamori, N.; Ma, L.; Edson, M.A.; Greenbaum, M.P.; Matzuk, M.M.TEX14 Interacts with CEP55 to Block Cell Abscission. *Mol. Cell. Biol.* **2010**, *30*, 2280–2292, doi:10.1128/MCB.01392-09.
155. Chang, Y.-C.; Chen, Y.-J.; Wu, C.-H.; Wu, Y.-C.; Yen, T.-C.; Ouyang, P. Characterization of Centrosomal Proteins Cep55 and Pericentrin in IntercellularBridges of Mouse Testes. *J. Cell. Biochem.* **2010**, *109*, 1274–1285, doi:10.1002/jcb.22517.
156. Zhao, W.; Seki, A.; Fang, G. Cep55, a Microtubule-Bundling Protein, Associates withCentralspindlin to Control the Midbody Integrity and Cell Abscission during Cytokinesis. *Mol. Biol. Cell* **2006**, *17*, 3881–3896, doi:10.1091/mbc.e06-01-0015.
157. Sinha, D.; Kalimutho, M.; Bowles, J.; Chan, A.-L.; Merriner, D.J.; Bain, A.L.; Simmons, J.L.; Freire, R.; Lopez, J.A.; Hobbs, R.M.; et al. Cep55 Overexpression Causes Male-Specific Sterility in Mice by Suppressing Foxo1 Nuclear Retention through Sustained Activation of PI3K/Akt Signaling. *FASEB J. Off. Publ. Fed. Am.Soc. Exp. Biol.* **2018**, *32*, 4984–4999, doi:10.1096/fj.201701096RR.
158. Wright, W.W.; Smith, L.; Kerr, C.; Charron, M. Mice That Express EnzymaticallyInactive Cathepsin L Exhibit Abnormal Spermatogenesis1. *Biol. Reprod.* **2003**, *68*,680–687, doi:10.1095/biolreprod.102.006726.
159. Zabludoff, S.D.; Charron, M.; DeCerbo, J.N.; Simukova, N.; Wright, W.W. Male GermCells Regulate Transcription of the Cathepsin l Gene by Rat Sertoli Cells. *Endocrinology* **2001**, *142*, 2318–2327, doi:10.1210/endo.142.6.8106.
160. Charron, M.; Folmer, J.S.; Wright, W.W. A 3-Kilobase Region Derived from the RatCathepsin L Gene Directs in Vivo Expression of a Reporter Gene in Sertoli Cells in aManner Comparable to That of the Endogenous Gene. *Biol. Reprod.* **2003**, *68*, 1641–1648, doi:10.1095/biolreprod.102.011619.
161. Charron, M.; Chern, J.-Y.; Wright, W.W. The Cathepsin L First Intron Stimulates GeneExpression in Rat Sertoli Cells. *Biol. Reprod.* **2007**, *76*, 813–824, doi:10.1095/biolreprod.106.057851.
162. Visone, T.; Charron, M.; Wright, W.W. Activation and Repression Domains within thePromoter of the Rat Cathepsin L Gene Orchestrate Sertoli Cell-Specific and Stage- Specific Gene Transcription in Transgenic Mice. *Biol. Reprod.* **2009**, *81*, 571–579, doi:10.1095/biolreprod.109.075952.
163. Kim, G.H.; Wright, W.W. A Comparison of the Effects of Testicular Maturation and Aging on the Stage-Specific Expression of CP-2/Cathepsin L Messenger RibonucleicAcid by Sertoli Cells of the Brown Norway Rat. *Biol. Reprod.* **1997**, *57*, 1467–1477, doi:10.1095/biolreprod57.6.1467.
164. Sluka, P.; O'Donnell, L.; Stanton, P.G. Stage-Specific Expression of Genes Associatedwith Rat Spermatogenesis: Characterization by Laser-Capture Microdissection and Real-Time Polymerase Chain Reaction. *Biol. Reprod.* **2002**, *67*, 820–828,doi:10.1095/biolreprod.102.004879.
165. Gye, M.C.; Kim, S.T. Expression of Cathepsin L in Human Testis under DiverseInfertility Conditions. *Arch. Androl.* **2004**, *50*, 187–191, doi:10.1080/01485010490425223.
166. Mathur, P.P.; Grima, J.; Mo, M.Y.; Zhu, L.J.; Aravindan, G.R.; Calcagno, K.; O'Bryan,M.; Chung, S.; Mruk, D.; Lee, W.M.; et al. Differential Expression of Multiple Cathepsin MRNAs in the Rat Testis during Maturation and Following Lonidamine Induced Tissue Restructuring. *Biochem. Mol. Biol. Int.* **1997**, *42*, 217–233, doi:10.1080/15216549700202611.
167. Erickson-Lawrence, M.; Zabludoff, S.D.; Wright, W.W. Cyclic Protein-2, a SecretoryProduct of Rat Sertoli Cells, Is the Proenzyme Form of Cathepsin L. *Mol. Endocrinol. Baltim. Md* **1991**, *5*, 1789–1798, doi:10.1210/mend-5-12-1789.
168. Mruk, D.; Zhu, L.J.; Silvestrini, B.; Lee, W.M.; Cheng, C.Y. Interactions of Proteasesand Protease Inhibitors in Sertoli-Germ Cell Cocultures Preceding the Formation of Specialized Sertoli-Germ Cell Junctions in Vitro. *J. Androl.* **1997**, *18*, 612–622.
169. Fan, H.-Y.; Sun, Q.-Y.; Zou, H. Regulation of Separase in Meiosis: Separase Is Activated at the Metaphase I-II Transition in Xenopus Oocytes during Meiosis. *Cell Cycle Georget. Tex* **2006**, *5*, 198–204, doi:10.4161/cc.5.2.2321.

170. Kudo, N.R.; Anger, M.; Peters, A.H.F.M.; Stemmann, O.; Theussl, H.-C.; Helmhart, W.; Kudo, H.; Heyting, C.; Nasmyth, K. Role of Cleavage by Separase of the Rec8 Kleisin Subunit of Cohesin during Mammalian Meiosis I. *J. Cell Sci.* **2009**, *122*, 2686–2698, doi:10.1242/jcs.035287.
171. Stemmann, O.; Zou, H.; Gerber, S.A.; Gygi, S.P.; Kirschner, M.W. Dual Inhibition ofSister Chromatid Separation at Metaphase. *Cell* **2001**, *107*, 715–726, doi:10.1016/s0092-8674(01)00603-1.
172. Sun, Y.; Kucej, M.; Fan, H.-Y.; Yu, H.; Sun, Q.-Y.; Zou, H. Separase Is Recruited toMitotic Chromosomes to Dissolve Sister Chromatid Cohesion in a DNA-Dependent Manner. *Cell* **2009**, *137*, 123–132, doi:10.1016/j.cell.2009.01.040.
173. Kucej, M.; Zou, H. DNA-Dependent Cohesin Cleavage by Separase. *Nucl. Austin Tex*
174. **2010**, *1*, 4–7, doi:10.4161/nucl.1.1.10010.
175. Touati, S.A.; Cladière, D.; Lister, L.M.; Leontiou, I.; Chambon, J.-P.; Rattani, A.; Böttger, F.; Stemmann, O.; Nasmyth, K.; Herbert, M.; et al. Cyclin A2 Is Required forSister Chromatid Segregation, but Not Separase Control, in Mouse Oocyte Meiosis. *Cell Rep.* **2012**, *2*, 1077–1087, doi:10.1016/j.celrep.2012.10.002.
176. Katis, V.L.; Lipp, J.J.; Imre, R.; Bogdanova, A.; Okaz, E.; Habermann, B.; Mechtler, K.; Nasmyth, K.; Zachariae, W. Rec8 Phosphorylation by Casein Kinase 1 and Cdc7- Dbf4 Kinase Regulates Cohesin Cleavage by Separase during Meiosis. *Dev. Cell* **2010**,*18*, 397–409, doi:10.1016/j.devcel.2010.01.014.
177. Tategu, M.; Nakagawa, H.; Sasaki, K.; Yamauchi, R.; Sekimachi, S.; Suita, Y.; Watanabe, N.; Yoshid, K. Transcriptional Regulation of Human Polo-like Kinases andEarly Mitotic Inhibitor. *J. Genet. Genomics Yi Chuan Xue Bao* **2008**, *35*, 215–224, doi:10.1016/S1673-8527(08)60030-2.
178. Guardavaccaro, D.; Kudo, Y.; Boulaire, J.; Barchi, M.; Busino, L.; Donzelli, M.; Margottin-Goguet, F.; Jackson, P.K.; Yamasaki, L.; Pagano, M. Control of Meiotic andMitotic Progression by the F Box Protein Beta-Trcp1 in Vivo. *Dev. Cell* **2003**, *4*, 799– 812, doi:10.1016/s1534-5807(03)00154-0.
179. Margottin-Goguet, F.; Hsu, J.Y.; Loktev, A.; Hsieh, H.M.; Reimann, J.D.R.; Jackson,
180. P.K. Prophase Destruction of Emi1 by the SCF(BetaTrCP/Slimb) Ubiquitin Ligase Activates the Anaphase Promoting Complex to Allow Progression beyond Prometaphase. *Dev. Cell* **2003**, *4*, 813–826, doi:10.1016/s1534-5807(03)00153-9.
181. Moshe, Y.; Bar-On, O.; Ganoth, D.; Hershko, A. Regulation of the Action of EarlyMitotic Inhibitor 1 on the Anaphase-Promoting Complex/Cyclosome by Cyclin- Dependent Kinases. *J. Biol. Chem.* **2011**, *286*, 16647–16657, doi:10.1074/jbc.M111.223339.
182. Zhi, E.; Li, P.; Chen, H.; Xu, P.; Zhu, X.; Zhu, Z.; He, Z.; Li, Z. Decreased Expressionof KIFC1 in Human Testes with Globozoospermic Defects. *Genes* **2016**, *7*, E75, doi:10.3390/genes7100075.
183. Yang, W.-X.; Sperry, A.O. C-Terminal Kinesin Motor KIFC1 Participates in AcrosomeBiogenesis and Vesicle Transport. *Biol. Reprod.* **2003**, *69*, 1719–1729, doi:10.1095/biolreprod.102.014878.
184. Ma, D.-D.; Wang, D.-H.; Yang, W.-X. Kinesins in Spermatogenesis. *Biol. Reprod.*
185. **2017**, *96*, 267–276, doi:10.1095/biolreprod.116.144113.
186. Janke, C.; Ortiz, J.; Lechner, J.; Shevchenko, A.; Shevchenko, A.; Magiera, M.M.; Schramm, C.; Schiebel, E. The Budding Yeast Proteins Spc24p and Spc25p Interactwith Ndc80p and Nuf2p at the Kinetochore and Are Important for Kinetochore Clustering and Checkpoint Control. *EMBO J.* **2001**, *20*, 777–791, doi:10.1093/emboj/20.4.777.
187. McCleland, M.L.; Kallio, M.J.; Barrett-Wilt, G.A.; Kestner, C.A.; Shabanowitz, J.; Hunt, D.F.; Gorbsky, G.J.; Stukenberg, P.T. The Vertebrate Ndc80 Complex ContainsSpc24 and Spc25 Homologs, Which Are Required to Establish and Maintain Kinetochore-Microtubule Attachment. *Curr. Biol. CB* **2004**, *14*, 131–137, doi:10.1016/j.cub.2003.12.058.
188. Danyu, L.; Ying, L.; Zhenwu, B.; Heming, Y.; Xuejun, L. Aquaporin 1 Expression inthe Testis, Epididymis and Vas Deferens of Postnatal ICR Mice. *Cell Biol. Int.* **2008**,*32*, 532–541, doi:10.1016/j.cellbi.2008.01.002.
189. Domeniconi, R.F.; Orsi, A.M.; Justulin, L.A.; Leme Beu, C.C.; Felisbino, S.L. Immunolocalization of Aquaporins 1, 2 and 7 in Rete Testis, Efferent Ducts, Epididymis and Vas Deferens of Adult Dog. *Cell Tissue Res.* **2008**, *332*, 329–335,doi:10.1007/s00441-008-0592-x.
190. Badran, H.H.; Hermo, L.S. Expression and Regulation of Aquaporins 1, 8, and 9 in theTestis, Efferent Ducts, and Epididymis of Adult Rats and during Postnatal Development. *J. Androl.* **2002**, *23*, 358–373.
191. Barbonetti, A.; Vassallo, M.R.C.; Antonangelo, C.; Nuccetelli, V.; D'Angeli, A.; Pelliccione, F.; Giorgi, M.; Francavilla, F.; Francavilla, S. RANTES and Human SpermFertilizing Ability: Effect on Acrosome Reaction and Sperm/Oocyte Fusion. *Mol. Hum.Reprod.* **2008**, *14*, 387–391, doi:10.1093/molehr/gan031.
192. Rebourcet, D.; Odet, F.; Vérot, A.; Combe, E.; Meugnier, E.; Pesenti, S.; Leduque, P.;Déchaud, H.; Magre, S.; Le Magueresse-Battistoni, B. The Effects of an in Utero Exposure to 2,3,7,8-Tetrachloro-Dibenzo-p-Dioxin on Male Reproductive Function: Identification of Ccl5 as a Potential Marker. *Int. J. Androl.* **2010**, *33*, 413–424, doi:10.1111/j.1365-2605.2009.01020.x.
193. Schall, T.J. Biology of the RANTES/SIS Cytokine Family. *Cytokine* **1991**, *3*, 165–183, doi:10.1016/1043-4666(91)90013-4.

194. Klein, B.; Haggeney, T.; Fietz, D.; Indumathy, S.; Loveland, K.L.; Hedger, M.; Kliesch, S.; Weidner, W.; Bergmann, M.; Schuppe, H.-C. Specific Immune Cell and Cytokine Characteristics of Human Testicular Germ Cell Neoplasia. *Hum. Reprod. Oxf.Engl.* **2016**, *31*, 2192–2202, doi:10.1093/humrep/dew211.
195. Müllenbach, E.; Walter, L.; Dressel, R. A Novel Discoidin Domain Receptor 1 (Ddr1)Transcript Is Expressed in Postmeiotic Germ Cells of the Rat Testis Depending on theMajor Histocompatibility Complex Haplotype. *Gene* **2006**, *372*, 53–61, doi:10.1016/j.gene.2005.12.004.
196. Ramaswamy, S.; Weinbauer, G.F. Endocrine Control of Spermatogenesis: Role of FSHand LH/ Testosterone. *Spermatogenesis* **2014**, *4*, e996025, 1–14, doi:10.1080/21565562.2014.996025.
197. Chieffi, P.; Troncone, G.; Caleo, A.; Libertini, S.; Linardopoulos, S.; Tramontano, D.;Portella, G. Aurora B Expression in Normal Testis and Seminomas. *J. Endocrinol.* **2004**, *181*, 263–270, doi:10.1677/joe.0.1810263.
198. Tang, C.-J.C.; Lin, C.-Y.; Tang, T.K. Dynamic Localization and Functional Implications of Aurora-C Kinase during Male Mouse Meiosis. *Dev. Biol.* **2006**, *290*,398–410, doi:10.1016/j.ydbio.2005.11.036.
199. Kimmins, S.; Crosio, C.; Kotaja, N.; Hirayama, J.; Monaco, L.; Höög, C.; van Duin, M.; Gossen, J.A.; Sassone-Corsi, P. Differential Functions of the Aurora-B and Aurora-C Kinases in Mammalian Spermatogenesis. *Mol. Endocrinol.* **2007**, *21*, 726–739, doi:10.1210/me.2006-0332.
200. Shuda, K.; Schindler, K.; Ma, J.; Schultz, R.M.; Donovan, P.J. Aurora Kinase B Modulates Chromosome Alignment in Mouse Oocytes. *Mol. Reprod. Dev.* **2009**, *76*,1094–1105, doi:10.1002/mrd.21075.
201. Mallampalli, R.K.; Glasser, J.R.; Coon, T.A.; Chen, B.B. Calmodulin Protects AuroraB on the Midbody to Regulate the Fidelity of Cytokinesis. *Cell Cycle Georget. Tex* **2013**, *12*, 663–673, doi:10.4161/cc.23586.
202. Ditchfield, C.; Johnson, V.L.; Tighe, A.; Ellston, R.; Haworth, C.; Johnson, T.; Mortlock, A.; Keen, N.; Taylor, S.S. Aurora B Couples Chromosome Alignment with Anaphase by Targeting BubR1, Mad2, and Cenp-E to Kinetochores. *J. Cell Biol.* **2003**,*161*, 267–280, doi:10.1083/jcb.200208091.
203. Maia, A.F.; Feijão, T.; Vromans, M.J.M.; Sunkel, C.E.; Lens, S.M.A. Aurora B KinaseCooperates with CENP-E to Promote Timely Anaphase Onset. *Chromosoma* **2010**, *119*, 405–413, doi:10.1007/s00412-010-0265-x.
204. Hori, T.; Haraguchi, T.; Hiraoka, Y.; Kimura, H.; Fukagawa, T. Dynamic Behavior ofNuf2-Hec1 Complex That Localizes to the Centrosome and Centromere and Is Essential for Mitotic Progression in Vertebrate Cells. *J. Cell Sci.* **2003**, *116*, 3347, doi:10.1242/jcs.00645.
205. Asakawa, H.; Hayashi, A.; Haraguchi, T.; Hiraoka, Y. Dissociation of the Nuf2-Ndc80Complex Releases Centromeres from the Spindle-Pole Body during Meiotic Prophase in Fission Yeast. *Mol. Biol. Cell* **2005**, *16*, 2325–2338, doi:10.1091/mbc.e04-11-0996.
206. Zhang, T.; Zhou, Y.; Qi, S.-T.; Wang, Z.-B.; Qian, W.-P.; Ouyang, Y.-C.; Shen, W.; Schatten, H.; Sun, Q.-Y. Nuf2 Is Required for Chromosome Segregation during MouseOocyte Meiotic Maturation. *Cell Cycle Georget. Tex* **2015**, *14*, 2701–2710, doi:10.1080/15384101.2015.1058677.
207. Godet, M.; Thomas, A.; Rudkin, B.B.; Durand, P. Developmental Changes in Cyclin B1 and Cyclin-Dependent Kinase 1 (CDK1) Levels in the Different Populations of Spermatogenic Cells of the Post-Natal Rat Testis. *Eur. J. Cell Biol.* **2000**, *79*, 816–823,doi:10.1078/0171-9335-00107.
208. Chapman, D.L.; Wolgemuth, D.J. Regulation of M-Phase Promoting Factor Activityduring Development of Mouse Male Germ Cells. *Dev. Biol.* **1994**, *165*, 500–506, doi:10.1006/dbio.1994.1270.
209. Gromoll, J.; Wessels, J.; Rosiepen, G.; Brinkworth, M.H.; Weinbauer, G.F. Expressionof Mitotic Cyclin B1 Is Not Confined to Proliferating Cells in the Rat Testis. *Biol. Reprod.* **1997**, *57*, 1312–1319, doi:10.1095/biolreprod57.6.1312.
210. Lau, Y.-F.C.; Li, Y.; Kido, T. Role of the Y-Located Putative Gonadoblastoma Gene inHuman Spermatogenesis. *Syst. Biol. Reprod. Med.* **2011**, *57*, 27–34, doi:10.3109/19396368.2010.499157.
211. Tang, J.-X.; Li, J.; Cheng, J.-M.; Hu, B.; Sun, T.-C.; Li, X.-Y.; Batool, A.; Wang, Z.-P.; Wang, X.-X.; Deng, S.-L.; et al. Requirement for CCNB1 in Mouse Spermatogenesis. *Cell Death Dis.* **2017**, *8*, e3142, doi:10.1038/cddis.2017.555.
212. Papadopoulos, V.; Miller, W.L. Role of Mitochondria in Steroidogenesis. *Best Pract.Res. Clin. Endocrinol. Metab.* **2012**, *26*, 771–790, doi:10.1016/j.beem.2012.05.002.
213. Tanaka, K.; Parvinen, M.; Nigg, E.A. The in Vivo Expression Pattern of Mouse Nek2,a NIMA-Related Kinase, Indicates a Role in Both Mitosis and Meiosis. *Exp. Cell Res.* **1997**, *237*, 264–274, doi:10.1006/excr.1997.3788.
214. Rhee, K.; Wolgemuth, D.J. Tcp10 Promoter-Directed Expression of the Nek2 Gene inMouse Meiotic Spermatocytes. *Mol. Cells* **2002**, *13*, 85–90.
215. Di Agostino, S.; Fedele, M.; Chieffi, P.; Fusco, A.; Rossi, P.; Geremia, R.; Sette, C. Phosphorylation of High-Mobility Group Protein A2 by Nek2 Kinase during the FirstMeiotic Division in Mouse Spermatocytes. *Mol. Biol. Cell* **2004**, *15*, 1224–1232, doi:10.1091/mbc.e03-09-0638.
216. Wei, R.; Ngo, B.; Wu, G.; Lee, W.-H. Phosphorylation of the Ndc80 Complex Protein,HEC1, by Nek2 Kinase Modulates Chromosome Alignment and Signaling of the Spindle Assembly Checkpoint. *Mol. Biol. Cell* **2011**, *22*, 3584–3594, doi:10.1091/mbc.E11-01-0012.

217. Welch, J.E.; O'Rand, M.G. Characterization of a Sperm-Specific Nuclear Autoantigenic Protein. II. Expression and Localization in the Testis. *Biol. Reprod.***1990**, *43*, 569–578, doi:10.1095/biolreprod43.4.569.

218. O'Rand, M.G.; Richardson, R.T.; Zimmerman, L.J.; Widgren, E.E. Sequence and Localization of Human NASP: Conservation of a Xenopus Histone-Binding Protein.*Dev. Biol.* **1992**, *154*, 37–44, doi:10.1016/0012-1606(92)90045-i.

219. Richardson, R.T.; Batova, I.N.; Widgren, E.E.; Zheng, L.X.; Whitfield, M.; Marzluff,W.F.; O'Rand, M.G. Characterization of the Histone H1-Binding Protein, NASP, as aCell Cycle-Regulated Somatic Protein. *J. Biol. Chem.* **2000**, *275*, 30378–30386, doi:10.1074/jbc.M003781200.

220. Heindel, J.J.; Steinberger, A.; Strada, S.J. Identification and Characterization of a Beta1-Adrenergic Receptor in the Rat Sertoli Cell. *Mol. Cell. Endocrinol.* **1981**, *22*, 349– 358, doi:10.1016/0303-7207(81)90043-5.

221. Hellgren, I.; Sylvén, C.; Magnusson, Y. Study of the Beta1 Adrenergic Receptor Expression in Human Tissues: Immunological Approach. *Biol. Pharm. Bull.* **2000**, *23*,700–703, doi:10.1248/bpb.23.700.

222. Gao, Y.; Qin, L.; Yang, Y.; Dong, X.; Zhao, Z.; Zhang, G.; Zhao, Z. PDPN Gene Promotes the Proliferation of Immature Bovine Sertoli Cells in Vitro. *Anim. Reprod. Sci.* **2017**, *179*, 35–43, doi:10.1016/j.anireprosci.2017.01.014.

223. Sun, F.; Xu, Q.; Zhao, D.; Degui Chen, C. Id4 Marks Spermatogonial Stem Cells in theMouse Testis. *Sci. Rep.* **2015**, *5*, 17594, doi:10.1038/srep17594.

224. Oatley, M.J.; Kaucher, A.V.; Racicot, K.E.; Oatley, J.M. Inhibitor of DNA Binding 4 IsExpressed Selectively by Single Spermatogonia in the Male Germline and Regulates the Self-Renewal of Spermatogonial Stem Cells in Mice. *Biol. Reprod.* **2011**, *85*, 347– 356, doi:10.1095/biolreprod.111.091330.

225. Helsel, A.R.; Yang, Q.-E.; Oatley, M.J.; Lord, T.; Sablitzky, F.; Oatley, J.M. ID4 Levels Dictate the Stem Cell State in Mouse Spermatogonia. *Dev. Camb. Engl.* **2017**,*144*, 624–634, doi:10.1242/dev.146928.

226. Zhang, H.; Zhang, X.; Ji, S.; Hao, C.; Mu, Y.; Sun, J.; Hao, J. Sohlh2 Inhibits OvarianCancer Cell Proliferation by Upregulation of P21 and Downregulation of Cyclin D1. *Carcinogenesis* **2014**, *35*, 1863–1871, doi:10.1093/carcin/bgu113.

227. Park, M.; Lee, Y.; Jang, H.; Lee, O.-H.; Park, S.-W.; Kim, J.-H.; Hong, K.; Song, H.; Park, S.-P.; Park, Y.-Y.; et al. SOHLH2 Is Essential for Synaptonemal Complex Formation during Spermatogenesis in Early Postnatal Mouse Testes. *Sci. Rep.* **2016**, *6*,20980–20980, doi:10.1038/srep20980.

228. Hao, J.; Yamamoto, M.; Richardson, T.E.; Chapman, K.M.; Denard, B.S.; Hammer, R.E.; Zhao, G.Q.; Hamra, F.K. Sohlh2 Knockout Mice Are Male-Sterile Because of Degeneration of Differentiating Type A Spermatogonia. *Stem Cells Dayt. Ohio* **2008**,*26*, 1587–1597, doi:10.1634/stemcells.2007-0502.

229. Zhang, X.; Liu, R.; Su, Z.; Zhang, Y.; Zhang, W.; Liu, X.; Wang, F.; Guo, Y.; Li, C.;Hao, J. Immunohistochemical Study of Expression of Sohlh1 and Sohlh2 in Normal Adult Human Tissues. *PloS One* **2015**, *10*, e0137431, doi:10.1371/journal.pone.0137431.

230. Barrios, F.; Filipponi, D.; Campolo, F.; Gori, M.; Bramucci, F.; Pellegrini, M.; Ottolenghi, S.; Rossi, P.; Jannini, E.A.; Dolci, S. SOHLH1 and SOHLH2 Control KitExpression during Postnatal Male Germ Cell Development. *J. Cell Sci.* **2012**, *125*, 1455–1464, doi:10.1242/jcs.092593.

231. Desimio, M.G.; Campolo, F.; Dolci, S.; De Felici, M.; Farini, D. SOHLH1 and SOHLH2 Directly Down-Regulate STIMULATED BY RETINOIC ACID 8 (STRA8)Expression. *Cell Cycle Georget. Tex* **2015**, *14*, 1036–1045, doi:10.1080/15384101.2015.1007721.

232. O'Bryan, M.K.; Mallidis, C.; Murphy, B.F.; Baker, H.W. Immunohistological Localization of Clusterin in the Male Genital Tract in Humans and Marmosets. *Biol. Reprod.* **1994**, *50*, 502–509, doi:10.1095/biolreprod50.3.502.

233. Ahuja, H.S.; Tenniswood, M.; Zakeri, Z.F. Differential Expression of Clusterin in the Testis and Epididymis of Postnatal and Germ Cell Deficient Mice. *J. Androl.* **1996**, *17*,491–501.

234. Han, Z.; Wang, Z.; Cheng, G.; Liu, B.; Li, P.; Li, J.; Wang, W.; Yin, C.; Zhang, W.Presence, Localization, and Origin of Clusterin in Normal Human Spermatozoa. *J. Assist. Reprod. Genet.* **2012**, *29*, 751–757, doi:10.1007/s10815-012-9779-x.

235. Matsushita, K.; Miyake, H.; Chiba, K.; Fujisawa, M. Clusterin Produced by Sertoli Cells Inhibits Heat Stress-Induced Apoptosis in the Rat Testis. *Andrologia* **2016**, *48*,11–19, doi:10.1111/and.12404.

236. Saewu, A.; Kadunganattil, S.; Raghupathy, R.; Kongmanas, K.; Diaz-Astudillo, P.;Hermo, L.; Tanphaichitr, N. Clusterin in the Mouse Epididymis: Possible Roles in Sperm Maturation and Capacitation. *Reprod. Camb. Engl.* **2017**, *154*, 867–880, doi:10.1530/REP-17-0518.

237. Zindy, F.; den Besten, W.; Chen, B.; Rehg, J.E.; Latres, E.; Barbacid, M.; Pollard, J.W.; Sherr, C.J.; Cohen, P.E.; Roussel, M.F. Control of Spermatogenesis in Mice by the Cyclin D-Dependent Kinase Inhibitors P18(Ink4c) and P19(Ink4d). *Mol. Cell. Biol.***2001**, *21*, 3244–3255, doi:10.1128/MCB.21.9.3244-3255.2001.

238. Buchold, G.M.; Magyar, P.L.; Arumugam, R.; Lee, M.M.; O'Brien, D.A. P19Ink4d andP18Ink4c Cyclin-Dependent Kinase Inhibitors in the Male Reproductive Axis. *Mol. Reprod. Dev.* **2007**, *74*, 997–1007, doi:10.1002/mrd.20716.

239. Zalzali, H.; Rabeh, W.; Najjar, O.; Abi Ammar, R.; Harajly, M.; Saab, R. Interplaybetween P53 and Ink4c in Spermatogenesis and Fertility. *Cell Cycle Georget. Tex* **2018**, *17*, 643–651, doi:10.1080/15384101.2017.1421874.
240. Longin, J.; Guillaumot, P.; Chauvin, M.A.; Morera, A.M.; Le Magueresse-Battistoni,
241. B. MT1-MMP in Rat Testicular Development and the Control of Sertoli Cell ProMMP-2 Activation. *J. Cell Sci.* **2001**, *114*, 2125–2134.
242. Siu, M.K.Y.; Cheng, C.Y. Interactions of Proteases, Protease Inhibitors, and the Beta1Integrin/Laminin Gamma3 Protein Complex in the Regulation of Ectoplasmic Specialization Dynamics in the Rat Testis. *Biol. Reprod.* **2004**, *70*, 945–964, doi:10.1095/biolreprod.103.023606.
243. Yao, P.-L.; Lin, Y.-C.; Richburg, J.H. TNF Alpha-Mediated Disruption of Spermatogenesis in Response to Sertoli Cell Injury in Rodents Is Partially Regulated byMMP2. *Biol. Reprod.* **2009**, *80*, 581–589, doi:10.1095/biolreprod.108.073122.
244. Chen, H.; Fok, K.L.; Yu, S.; Jiang, J.; Chen, Z.; Gui, Y.; Cai, Z.; Chan, H.C. CD147 IsRequired for Matrix Metalloproteinases-2 Production and Germ Cell Migration duringSpermatogenesis. *Mol. Hum. Reprod.* **2011**, *17*, 405–414, doi:10.1093/molehr/gar013.
245. Ferrer, M.; Rodriguez, H.; Zara, L.; Yu, Y.; Xu, W.; Oko, R. MMP2 and Acrosin Are Major Proteinases Associated with the Inner Acrosomal Membrane and May Cooperatein Sperm Penetration of the Zona Pellucida during Fertilization. *Cell Tissue Res.* **2012**, *349*, 881–895, doi:10.1007/s00441-012-1429-1.
246. Warinrak, C.; Wu, J.-T.; Hsu, W.-L.; Liao, J.-W.; Chang, S.-C.; Cheng, F.-P. Expression of Matrix Metalloproteinases (MMP-2, MMP-9) and Their Inhibitors (TIMP-1, TIMP-2) in Canine Testis, Epididymis and Semen. *Reprod. Domest. Anim.Zuchthyg.* **2015**, *50*, 48–57, doi:10.1111/rda.12448.
247. Liu, S.-S.; Maguire, E.M.; Bai, Y.-S.; Huang, L.; Liu, Y.; Xu, L.; Fauzi, I.; Zhang, S.-Q.; Xiao, Q.; Ma, N.-F. A Novel Regulatory Axis, CHD1L-MicroRNA 486-Matrix Metalloproteinase 2, Controls Spermatogonial Stem Cell Properties. *Mol. Cell. Biol.* **2019**, *39*, e00357-18, doi:10.1128/MCB.00357-18.
248. Barone, R.; Pitruzzella, A.; Marino Gammazza, A.; Rappa, F.; Salerno, M.; Barone, F.;Sangiorgi, C.; D'Amico, D.; Locorotondo, N.; Di Gaudio, F.; et al. Nandrolone Decanoate Interferes with Testosterone Biosynthesis Altering Blood-Testis Barrier Components. *J. Cell. Mol. Med.* **2017**, *21*, 1636–1647, doi:10.1111/jcmm.13092.
249. Siu, M.K.Y.; Cheng, C.Y. Interactions of Proteases, Protease Inhibitors, and the Beta1Integrin/Laminin Gamma3 Protein Complex in the Regulation of Ectoplasmic Specialization Dynamics in the Rat Testis. *Biol. Reprod.* **2004**, *70*, 945–964, doi:10.1095/biolreprod.103.023606.
250. Beumer, T.L.; Roepers-Gajadien, H.L.; Gademan, I.S.; Kal, H.B.; de Rooij, D.G. Involvement of the D-Type Cyclins in Germ Cell Proliferation and Differentiation inthe Mouse. *Biol. Reprod.* **2000**, *63*, 1893–1898, doi:10.1095/biolreprod63.6.1893.
251. Lucas, T.F.G.; Lazari, M.F.M.; Porto, C.S. Differential Role of the Estrogen ReceptorsESR1 and ESR2 on the Regulation of Proteins Involved with Proliferation and Differentiation of Sertoli Cells from 15-Day-Old Rats. *Mol. Cell. Endocrinol.* **2014**, *382*, 84–96, doi:10.1016/j.mce.2013.09.015.
252. Lucas, T.F.G.; Lazari, M.F.M.; Porto, C.S. Differential Role of the Estrogen ReceptorsESR1 and ESR2 on the Regulation of Proteins Involved with Proliferation and Differentiation of Sertoli Cells from 15-Day-Old Rats. *Mol. Cell. Endocrinol.* **2014**, *382*, 84–96, doi:10.1016/j.mce.2013.09.015.
253. Rindone, G.M.; Gorga, A.; Regueira, M.; Pellizzari, E.H.; Cigorraga, S.B.; Galardo,M.N.; Meroni, S.B.; Riera, M.F. Metformin Counteracts the Effects of FSH on Rat Sertoli Cell Proliferation. *Reprod. Camb. Engl.* **2018**, *156*, 93–101, doi:10.1530/REP-18-0233.
254. Beumer, T.L.; Roepers-Gajadien, H.L.; Gademan, I.S.; Kal, H.B.; de Rooij, D.G. Involvement of the D-Type Cyclins in Germ Cell Proliferation and Differentiation inthe Mouse. *Biol. Reprod.* **2000**, *63*, 1893–1898, doi:10.1095/biolreprod63.6.1893.
255. Carthon, B.C.; Neumann, C.A.; Das, M.; Pawlyk, B.; Li, T.; Geng, Y.; Sicinski, P. Genetic Replacement of Cyclin D1 Function in Mouse Development by Cyclin D2. *Mol. Cell. Biol.* **2005**, *25*, 1081–1088, doi:10.1128/MCB.25.3.1081-1088.2005.
256. He, Z.; Jiang, J.; Kokkinaki, M.; Tang, L.; Zeng, W.; Gallicano, I.; Dobrinski, I.; Dym,
257. M. MiRNA-20 and Mirna-106a Regulate Spermatogonial Stem Cell Renewal at thePost-Transcriptional Level via Targeting STAT3 and Ccnd1. *Stem Cells Dayt. Ohio***2013**, *31*, 2205–2217, doi:10.1002/stem.1474.
258. Feki, A.; Jefford, C.E.; Jefford, C.-E.; Durand, P.; Harb, J.; Lucas, H.; Krause, K.-H.;Irminger-Finger, I. BARD1 Expression during Spermatogenesis Is Associated with Apoptosis and Hormonally Regulated. *Biol. Reprod.* **2004**, *71*, 1614–1624, doi:10.1095/biolreprod.104.029678.
259. Zhao, W.; Steinfeld, J.B.; Liang, F.; Chen, X.; Maranon, D.G.; Jian Ma, C.; Kwon, Y.;Rao, T.; Wang, W.; Sheng, C.; et al. BRCA1-BARD1 Promotes RAD51-Mediated Homologous DNA Pairing. *Nature* **2017**, *550*, 360–365, doi:10.1038/nature24060.
260. Wang, Y.-P.; Qi, S.-T.; Wei, Y.; Ge, Z.-J.; Chen, L.; Hou, Y.; Ouyang, Y.-C.; Schatten,H.; Zhao, J.-G.; Sun, Q.-Y. Knockdown of UCHL5IP Causes Abnormalities in γ- Tubulin Localisation, Spindle Organisation and

Chromosome Alignment in Mouse Oocyte Meiotic Maturation. *Reprod. Fertil. Dev.* **2013**, *25*, 495–502, doi:10.1071/RD12300.
261. Pandey, A.; Yadav, S.K.; Vishvkarma, R.; Singh, B.; Maikhuri, J.P.; Rajender, S.; Gupta, G. The Dynamics of Gene Expression during and Post Meiosis Sets the Sperm Agenda. *Mol. Reprod. Dev.* **2019**, doi:10.1002/mrd.23278.
262. Li, L.; Sha, Y.-W.; Su, Z.-Y.; Mei, L.-B.; Ji, Z.-Y.; Zhang, Q.; Lin, S.-B.; Wang, X.;Qiu, P.-P.; Li, P.; et al. A Novel Mutation in HAUS7 Results in Severe Oligozoospermia in Two Brothers. *Gene* **2018**, *639*, 106–110, doi:10.1016/j.gene.2017.10.014.
263. Ye, X.; Skinner, M.K.; Kennedy, G.; Chun, J. Age-Dependent Loss of Sperm Production in Mice via Impaired Lysophosphatidic Acid Signaling. *Biol. Reprod.* **2008**,*79*, 328–336, doi:10.1095/biolreprod.108.068783.
264. Ye, X. Lysophospholipid Signaling in the Function and Pathology of the ReproductiveSystem. *Hum. Reprod. Update* **2008**, *14*, 519–536, doi:10.1093/humupd/dmn023.
265. Schleicher, R.L.; Zheng, M.; Zhang, M. Immunocytochemical Localization and Endogenous Synthesis of Apolipoprotein E in Testicular Leydig Cells. *Biol. Reprod.***1993**, *48*, 313–324, doi:10.1095/biolreprod48.2.313.
266. Olson, L.M.; Zhou, X.; Schreiber, J.R. Immunolocalization of Apolipoprotein E in theTestis and Epididymis of the Rat. *Biol. Reprod.* **1994**, *50*, 535–542, doi:10.1095/biolreprod50.3.535.
267. Olson, L.M.; Zhou, X.; Schreiber, J.R. Cell-Specific Localization of Apolipoprotein EMessenger Ribonucleic Acid in the Testis and Epididymis of the Rat. *Biol. Reprod.* **1995**, *52*, 1003–1011, doi:10.1095/biolreprod52.5.1003.
268. Law, G.L.; McGuinness, M.P.; Linder, C.C.; Griswold, M.D. Expression of Apolipoprotein E MRNA in the Epithelium and Interstitium of the Testis and theEpididymis. *J. Androl.* **1997**, *18*, 32–42.
269. Fofana, M.; Maboundou, J.C.; Bocquet, J.; Le Goff, D. Transfer of Cholesterol betweenHigh Density Lipoproteins and Cultured Rat Sertoli Cells. *Biochem. Cell Biol. Biochim. Biol. Cell.* **1996**, *74*, 681–686, doi:10.1139/o96-074.
270. Kanimozhi, V.; Palanivel, K.; Akbarsha, M.A.; Kadalmani, B. Molecular Mechanismsof Tributyltin-Induced Alterations in Cholesterol Homeostasis and Steroidogenesis in Hamster Testis: In Vivo and in Vitro Studies. *J. Cell. Biochem.* **2018**, *119*, 4021–4037,doi:10.1002/jcb.26564.
271. Anderson, E.L.; Baltus, A.E.; Roepers-Gajadien, H.L.; Hassold, T.J.; de Rooij, D.G.; van Pelt, A.M.M.; Page, D.C. Stra8 and Its Inducer, Retinoic Acid, Regulate Meiotic Initiation in Both Spermatogenesis and Oogenesis in Mice. *Proc. Natl. Acad. Sci. U. S.A.* **2008**, *105*, 14976–14980, doi:10.1073/pnas.0807297105.
272. Lu, N.; Sargent, K.M.; Clopton, D.T.; Pohlmeier, W.E.; Brauer, V.M.; McFee, R.M.;Weber, J.S.; Ferrara, N.; Silversides, D.W.; Cupp, A.S. Loss of Vascular EndothelialGrowth Factor A (VEGFA) Isoforms in the Testes of Male Mice Causes Subfertility,Reduces Sperm Numbers, and Alters Expression of Genes That Regulate Undifferentiated Spermatogonia. *Endocrinology* **2013**, *154*, 4790–4802, doi:10.1210/en.2013-1363.
273. Bott, R.C.; McFee, R.M.; Clopton, D.T.; Toombs, C.; Cupp, A.S. Vascular EndothelialGrowth Factor and Kinase Domain Region Receptor Are Involved in Both Seminiferous Cord Formation and Vascular Development during Testis Morphogenesis in the Rat. *Biol. Reprod.* **2006**, *75*, 56–67, doi:10.1095/biolreprod.105.047225.
274. Caires, K.C.; de Avila, J.M.; Cupp, A.S.; McLean, D.J. VEGFA Family Isoforms Regulate Spermatogonial Stem Cell Homeostasis in Vivo. *Endocrinology* **2012**, *153*,887–900, doi:10.1210/en.2011-1323.
275. Caires, K.C.; de Avila, J.; McLean, D.J. Vascular Endothelial Growth Factor RegulatesGerm Cell Survival during Establishment of Spermatogenesis in the Bovine Testis. *Reprod. Camb. Engl.* **2009**, *138*, 667–677, doi:10.1530/REP-09-0020.
276. Baltes-Breitwisch, M.M.; Artac, R.A.; Bott, R.C.; McFee, R.M.; Kerl, J.G.; Clopton, D.T.; Cupp, A.S. Neutralization of Vascular Endothelial Growth Factor AntiangiogenicIsoforms or Administration of Proangiogenic Isoforms Stimulates Vascular Development in the Rat Testis. *Reprod. Camb. Engl.* **2010**, *140*, 319–329, doi:10.1530/REP-09-0456.
277. Oresti, G.M.; García-López, J.; Aveldaño, M.I.; Del Mazo, J. Cell-Type-Specific Regulation of Genes Involved in Testicular Lipid Metabolism: Fatty Acid-Binding Proteins, Diacylglycerol Acyltransferases, and Perilipin 2. *Reprod. Camb. Engl.* **2013**,*146*, 471–480, doi:10.1530/REP-13-0199.
278. Oresti, G.M.; García-López, J.; Aveldaño, M.I.; Del Mazo, J. Cell-Type-Specific Regulation of Genes Involved in Testicular Lipid Metabolism: Fatty Acid-Binding Proteins, Diacylglycerol Acyltransferases, and Perilipin 2. *Reprod. Camb. Engl.* **2013**,*146*, 471–480, doi:10.1530/REP-13-0199.
279. Gautam, M.; Bhattacharya, I.; Rai, U.; Majumdar, S.S. Hormone Induced DifferentialTranscriptome Analysis of Sertoli Cells during Postnatal Maturation of Rat Testes. *PloS One* **2018**, *13*, e0191201, doi:10.1371/journal.pone.0191201.
280. Huang, G.; Yuan, M.; Zhang, J.; Li, J.; Gong, D.; Li, Y.; Zhang, J.; Lin, P.; Huang, L. IL-6 Mediates Differentiation Disorder during Spermatogenesis in Obesity-AssociatedInflammation by Affecting the Expression of Zfp637 through the SOCS3/STAT3 Pathway. *Sci. Rep.* **2016**, *6*, 28012, doi:10.1038/srep28012.

281. Özkan, Z.S.; Deveci, D.; Akpolat, N.; İlhan, F.; Şimşek, M.; Timurkan, H. The Impactof Plasma SOCS3 Levels and Endometrial Leukocytes on Unexplained Infertility. *Turk. J. Med. Sci.* **2018**, *48*, 509–516, doi:10.3906/sag-1707-103.
282. Kokk, K.; Veräjänkorva, E.; Wu, X.-K.; Tapfer, H.; Põldoja, E.; Pöllänen, P. Immunohistochemical Detection of Glucose Transporters Class I Subfamily in theMouse, Rat and Human Testis. *Med. Kaunas Lith.* **2004**, *40*, 156–160.
283. Rauch, M.C.; Ocampo, M.E.; Bohle, J.; Amthauer, R.; Yáñez, A.J.; Rodríguez-Gil, J.E.; Slebe, J.C.; Reyes, J.G.; Concha, I.I. Hexose Transporters GLUT1 and GLUT3 Are Colocalized with Hexokinase I in Caveolae Microdomains of Rat SpermatogenicCells. *J. Cell. Physiol.* **2006**, *207*, 397–406, doi:10.1002/jcp.20582.
284. Zhang, J.; Ding, X.; Bian, Z.; Xia, Y.; Lu, C.; Wang, S.; Song, L.; Wang, X. The Effectof Anti-Eppin Antibodies on Ionophore A23187-Induced Calcium Influx and Acrosome Reaction of Human Spermatozoa. *Hum. Reprod. Oxf. Engl.* **2010**, *25*, 29–36,doi:10.1093/humrep/dep356.
285. O'Rand, M.G.; Widgren, E.E. Loss of Calcium in Human Spermatozoa via EPPIN, theSemenogelin Receptor. *Biol. Reprod.* **2012**, *86*, 55, doi:10.1095/biolreprod.111.094227.
286. Silva, E.J.R.; Patrão, M.T.C.C.; Tsuruta, J.K.; O'Rand, M.G.; Avellar, M.C.W. Epididymal Protease Inhibitor (EPPIN) Is Differentially Expressed in the Male Rat Reproductive Tract and Immunolocalized in Maturing Spermatozoa. *Mol. Reprod. Dev.***2012**, *79*, 832–842, doi:10.1002/mrd.22119.
287. Bian, Z.-H.; Zhang, J.; Ding, X.-L.; Zhang, B.; Wang, Z.-J.; Lu, C.-C.; Song, L.; Wang,S.-L.; Wang, X.-R. Localization of Epididymal Protease Inhibitor in Adult Rat and Its Transcription Profile in Testis during Postnatal Development. *Asian J. Androl.* **2009**, *11*, 731–739, doi:10.1038/aja.2009.66.
288. Zhang, J.; Ding, X.; Bian, Z.; Xia, Y.; Lu, C.; Wang, S.; Song, L.; Wang, X. The Effectof Anti-Eppin Antibodies on Ionophore A23187-Induced Calcium Influx and Acrosome Reaction of Human Spermatozoa. *Hum. Reprod. Oxf. Engl.* **2010**, *25*, 29–36,doi:10.1093/humrep/dep356.
289. Prisco, M.; Rosati, L.; Morgillo, E.; Mollica, M.P.; Agnese, M.; Andreuccetti, P.;Valiante, S. Pituitary Adenylate Cyclase-Activating Peptide (PACAP) and Its Receptors in Mus Musculus Testis. *Gen. Comp. Endocrinol.* **2020**, *286*, 113297, doi:10.1016/j.ygcen.2019.113297.
290. Reglodi, D.; Cseh, S.; Somoskoi, B.; Fulop, B.D.; Szentleleky, E.; Szegeczki, V.;Kovacs, A.; Varga, A.; Kiss, P.; Hashimoto, H.; et al. Disturbed Spermatogenic Signaling in Pituitary Adenylate Cyclase Activating Polypeptide-Deficient Mice.*Reprod. Camb. Engl.* **2018**, *155*, 129–139, doi:10.1530/REP-17-0470.
291. Persengiev, S.P.; Kondova, I.I.; Millette, C.F.; Kilpatrick, D.L. Gli Family Members Are Differentially Expressed during the Mitotic Phase of Spermatogenesis. *Oncogene* **1997**, *14*, 2259–2264, doi:10.1038/sj.onc.1201068.
292. Szczepny, A.; Hime, G.R.; Loveland, K.L. Expression of Hedgehog Signalling Components in Adult Mouse Testis. *Dev. Dyn. Off. Publ. Am. Assoc. Anat.* **2006**, *235*,3063–3070, doi:10.1002/dvdy.20931.
293. Yao, C.; Sun, M.; Yuan, Q.; Niu, M.; Chen, Z.; Hou, J.; Wang, H.; Wen, L.; Liu, Y.; Li, Z.; et al. MiRNA-133b Promotes the Proliferation of Human Sertoli Cells throughTargeting GLI3. *Oncotarget* **2016**, *7*, 2201–2219, doi:10.18632/oncotarget.6876.
294. Li, L.; Fan, L.; Peng, N.; Yang, L.; Mou, L.; Huang, W. R383C Mutation of HumanCDC20 Results in Idiopathic Non-Obstructive Azoospermia. *Oncotarget* **2017**, *8*, 99816–99824, doi:10.18632/oncotarget.21071.
295. Goto, M.; Eddy, E.M. Speriolin Is a Novel Spermatogenic Cell-Specific CentrosomalProtein Associated with the Seventh WD Motif of Cdc20. *J. Biol. Chem.* **2004**, *279*, 42128–42138, doi:10.1074/jbc.M403190200.
296. Goto, M.; Eddy, E.M. Speriolin Is a Novel Spermatogenic Cell-Specific CentrosomalProtein Associated with the Seventh WD Motif of Cdc20. *J. Biol. Chem.* **2004**, *279*, 42128–42138, doi:10.1074/jbc.M403190200.
297. Jin, F.; Hamada, M.; Malureanu, L.; Jeganathan, K.B.; Zhou, W.; Morbeck, D.E.; vanDeursen, J.M. Cdc20 Is Critical for Meiosis I and Fertility of Female Mice. *PLoS Genet.* **2010**, *6*, e1001147, doi:10.1371/journal.pgen.1001147.
298. Yang, W.L.; Li, J.; An, P.; Lei, A.M. CDC20 Downregulation Impairs Spindle Morphology and Causes Reduced First Polar Body Emission during Bovine OocyteMaturation. *Theriogenology* **2014**, *81*, 535–544, doi:10.1016/j.theriogenology.2013.11.005.
299. Eward, K.L.; Obermann, E.C.; Shreeram, S.; Loddo, M.; Fanshawe, T.; Williams, C.; Jung, H.-I.; Prevost, A.T.; Blow, J.J.; Stoeber, K.; et al. DNA Replication Licensing inSomatic and Germ Cells. *J. Cell Sci.* **2004**, *117*, 5875–5886, doi:10.1242/jcs.01503.
300. Arias, E.E.; Walter, J.C. Strength in Numbers: Preventing Rereplication via MultipleMechanisms in Eukaryotic Cells. *Genes Dev.* **2007**, *21*, 497–518, doi:10.1101/gad.1508907.
301. Lu, C.-H.; Lee, R.K.-K.; Hwu, Y.-M.; Chu, S.-L.; Chen, Y.-J.; Chang, W.-C.; Lin, S.-P.; Li, S.-H. SERPINE2, a Serine Protease Inhibitor Extensively Expressed in Adult Male Mouse Reproductive Tissues, May Serve as a Murine Sperm Decapacitation Factor. *Biol. Reprod.* **2011**, *84*, 514–525, doi:10.1095/biolreprod.110.085100.
302. Grive Dynamic Transcriptome Profiles within Spermatogonial and Spermatocyte Populations during Postnatal Testis Maturation Revealed by Single-Cell Sequen... -PubMed - NCBI. **2019**.

303. Hilbold, E.; Bergmann, M.; Fietz, D.; Kliesch, S.; Weidner, W.; Langeheine, M.; Rode,K.; Brehm, R. Immunolocalization of DMRTB1 in Human Testis with Normal and Impaired Spermatogenesis. *Andrology* **2019**, *7*, 428–440, doi:10.1111/andr.12617.
304. Lukassen, H.G.M.; van der Meer, A.; van Lierop, M.-J.C.; Lindeman, E.J.M.; Joosten, I.; Braat, D.D.M. The Proportion of Follicular Fluid CD16+CD56DIM NK Cells Is Increased in IVF Patients with Idiopathic Infertility. *J. Reprod. Immunol.* **2003**, *60*, 71–84, doi:10.1016/s0165-0378(03)00081-0.
305. Gil, T.; Castilla, J.A.; Hortas, M.L.; Redondo, M.; Samaniego, F.; Garrido, F.; Vergara,F.; Herruzo, A.J. Increase of Large Granular Lymphocytes in Human Ejaculate Containing Antisperm Antibodies. *Hum. Reprod. Oxf. Engl.* **1998**, *13*, 296–301, doi:10.1093/humrep/13.2.296.
306. Aksu, S.; Çalışkan, E.; Cakiroglu, Y. Evaluation of Endometrial Natural Killer Cell Expression of CD4, CD103, and CD16 Cells in Women with Unexplained Infertility. *J.Reprod. Immunol.* **2016**, *117*, 70–75, doi:10.1016/j.jri.2016.07.004.
307. Lehti, M.S.; Kotaja, N.; Sironen, A. KIF3A Is Essential for Sperm Tail Formation andManchette Function. *Mol. Cell. Endocrinol.* **2013**, *377*, 44–55, doi:10.1016/j.mce.2013.06.030.
308. Zhao, Y.-Q.; Mu, D.-L.; Wang, D.; Han, Y.-L.; Hou, C.-C.; Zhu, J.-Q. Analysis of theFunction of KIF3A and KIF3B in the Spermatogenesis in Boleophthalmus Pectinirostris. *Fish Physiol. Biochem.* **2018**, *44*, 769–788, doi:10.1007/s10695-017- 0461-1.
309. Wang, J.; Gao, X.; Zheng, X.; Hou, C.; Xie, Q.; Lou, B.; Zhu, J. Expression and Potential Functions of KIF3A/3B to Promote Nuclear Reshaping and Tail Formation during Larimichthys Polyactis Spermiogenesis. *Dev. Genes Evol.* **2019**, *229*, 161–181,doi:10.1007/s00427-019-00637-5.
310. Kanehara, H.; Song, K.; Hirai, K.; Ueda, H.; Shiota, N.; Azuma, H.; Katsuoka, Y.; Miyazaki, H.; Miyazaki, M. Involvement of Angiotensin II Receptor Subtypes duringTesticular Development in Rats. *Int. J. Androl.* **1998**, *21*, 186–195.
311. Gianzo, M.; Muñoa-Hoyos, I.; Urizar-Arenaza, I.; Larreategui, Z.; Quintana, F.; Garrido, N.; Subirán, N.; Irazusta, J. Angiotensin II Type 2 Receptor Is Expressed in Human Sperm Cells and Is Involved in Sperm Motility. *Fertil. Steril.* **2016**, *105*, 608–616, doi:10.1016/j.fertnstert.2015.11.004.
312. Hirai, K.; Song, K.; Kanehara, H.; Shiota, N.; Ueda, H.; Kim, S.; Miyazaki, H.; Katsuoka, Y.; Miyazaki, M. Pituitary-Dependent Expression of the Testicular Angiotensin II Receptor and Its Subtypes in Rats. *Int. J. Androl.* **1998**, *21*, 177–185,doi:10.1046/j.1365-2605.1998.00102.x.
313. Godet, M.; Damestoy, A.; Mouradian, S.; Rudkin, B.B.; Durand, P. Key Role forCyclin-Dependent Kinases in the First and Second Meiotic Divisions of Rat Spermatocytes. *Biol. Reprod.* **2004**, *70*, 1147–1152, doi:10.1095/biolreprod.103.023705.
314. Redgrove, K.A.; Bernstein, I.R.; Pye, V.J.; Mihalas, B.P.; Sutherland, J.M.; Nixon, B.;McCluskey, A.; Robinson, P.J.; Holt, J.E.; McLaughlin, E.A. Dynamin 2 Is Essential for Mammalian Spermatogenesis. *Sci. Rep.* **2016**, *6*, 35084, doi:10.1038/srep35084.
315. Rattani, A.; Vinod, P.K.; Godwin, J.; Tachibana-Konwalski, K.; Wolna, M.; Malumbres, M.; Novák, B.; Nasmyth, K. Dependency of the Spindle Assembly Checkpoint on Cdk1 Renders the Anaphase Transition Irreversible. *Curr. Biol. CB***2014**, *24*, 630–637, doi:10.1016/j.cub.2014.01.033.
316. Clement, T.M.; Inselman, A.L.; Goulding, E.H.; Willis, W.D.; Eddy, E.M. DisruptingCyclin Dependent Kinase 1 in Spermatocytes Causes Late Meiotic Arrest and Infertility in Mice. *Biol. Reprod.* **2015**, *93*, 137–137, doi:10.1095/biolreprod.115.134940.
317. Li, J.; Ouyang, Y.-C.; Zhang, C.-H.; Qian, W.-P.; Sun, Q.-Y. The Cyclin B2/CDK1Complex Inhibits Separase Activity in Mouse Oocyte Meiosis I. *Dev. Camb. Engl.* **2019**, *146*, dev182519, doi:10.1242/dev.182519.
318. Wei, X.; Gao, C.; Luo, J.; Zhang, W.; Qi, S.; Liang, W.; Dai, S. Hec1 Inhibition AltersSpindle Morphology and Chromosome Alignment in Porcine Oocytes. *Mol. Biol. Rep.***2014**, *41*, 5089–5095, doi:10.1007/s11033-014-3374-4.
319. DeLuca, J.G.; Dong, Y.; Hergert, P.; Strauss, J.; Hickey, J.M.; Salmon, E.D.; McEwen,
320. B.F. Hec1 and Nuf2 Are Core Components of the Kinetochore Outer Plate Essential forOrganizing Microtubule Attachment Sites. *Mol. Biol. Cell* **2005**, *16*, 519–531, doi:10.1091/mbc.e04-09-0852.
321. Sun, S.-C.; Zhang, D.-X.; Lee, S.-E.; Xu, Y.-N.; Kim, N.-H. Ndc80 Regulates MeioticSpindle Organization, Chromosome Alignment, and Cell Cycle Progression in MouseOocytes. *Microsc. Microanal.* **2011**, *17*, 431–439, doi:10.1017/S1431927611000274.
322. Diaz-Rodríguez, E.; Sotillo, R.; Schvartzman, J.-M.; Benezra, R. Hec1 OverexpressionHyperactivates the Mitotic Checkpoint and Induces Tumor Formation in Vivo. *Proc. Natl. Acad. Sci. U. S. A.* **2008**, *105*, 16719–16724, doi:10.1073/pnas.0803504105.
323. DeLuca, J.G.; Howell, B.J.; Canman, J.C.; Hickey, J.M.; Fang, G.; Salmon, E.D. Nuf2and Hec1 Are Required for Retention of the Checkpoint Proteins Mad1 and Mad2 to Kinetochores. *Curr. Biol. CB* **2003**, *13*, 2103–2109, doi:10.1016/j.cub.2003.10.056.

324. Lin, Y.-T.; Chen, Y.; Wu, G.; Lee, W.-H. Hec1 Sequentially Recruits Zwint-1 and ZW10 to Kinetochores for Faithful Chromosome Segregation and Spindle Checkpoint Control. *Oncogene* **2006**, *25*, 6901–6914, doi:10.1038/sj.onc.1209687.
325. Zhang, G.; Kelstrup, C.D.; Hu, X.-W.; Kaas Hansen, M.J.; Singleton, M.R.; Olsen,J.V.; Nilsson, J. The Ndc80 Internal Loop Is Required for Recruitment of the Ska Complex to Establish End-on Microtubule Attachment to Kinetochores. *J. Cell Sci.*
326. **2012**, *125*, 3243–3253, doi:10.1242/jcs.104208.
327. Helgeson, L.A.; Zelter, A.; Riffle, M.; MacCoss, M.J.; Asbury, C.L.; Davis, T.N. Human Ska Complex and Ndc80 Complex Interact to Form a Load-Bearing Assembly That Strengthens Kinetochore-Microtubule Attachments. *Proc. Natl. Acad. Sci. U. S. A.***2018**, *115*, 2740–2745, doi:10.1073/pnas.1718553115.
328. Chen, J.; Liao, A.; Powers, E.N.; Liao, H.; Kohlstaedt, L.A.; Evans, R.; Holly, R.M.; Kim, J.K.; Jovanovic, M.; Ünal, E. Aurora B-Dependent Ndc80 Degradation RegulatesKinetochore Composition in Meiosis. *Genes Dev.* **2020**, *34*, 209–225, doi:10.1101/gad.333997.119.
329. Gui, L.; Homer, H. Hec1-Dependent Cyclin B2 Stabilization Regulates the G2-M Transition and Early Prometaphase in Mouse Oocytes. *Dev. Cell* **2013**, *25*, 43–54,doi:10.1016/j.devcel.2013.02.008.
330. He, Z.; Jiang, J.; Kokkinaki, M.; Dym, M. Nodal Signaling via an Autocrine PathwayPromotes Proliferation of Mouse Spermatogonial Stem/Progenitor Cells through Smad2/3 and Oct-4 Activation. *Stem Cells Dayt. Ohio* **2009**, *27*, 2580–2590, doi:10.1002/stem.198.
331. Tian, R.-H.; Yang, S.; Zhu, Z.-J.; Wang, J.-L.; Liu, Y.; Yao, C.; Ma, M.; Guo, Y.; Yuan, Q.; Hai, Y.; et al. NODAL Secreted by Male Germ Cells Regulates the Proliferation and Function of Human Sertoli Cells from Obstructive Azoospermia andNonobstructive Azoospermia Patients. *Asian J. Androl.* **2015**, *17*, 996–1005, doi:10.4103/1008-682X.159722.
332. Spiller, C.M.; Feng, C.-W.; Jackson, A.; Gillis, A.J.M.; Rolland, A.D.; Looijenga, L.H.J.; Koopman, P.; Bowles, J. Endogenous Nodal Signaling Regulates Germ Cell Potency during Mammalian Testis Development. *Dev. Camb. Engl.* **2012**, *139*, 4123–4132, doi:10.1242/dev.083006.
333. Tassinari, V.; Campolo, F.; Cesarini, V.; Todaro, F.; Dolci, S.; Rossi, P. Fgf9 Inhibitionof Meiotic Differentiation in Spermatogonia Is Mediated by Erk-Dependent Activation of Nodal-Smad2/3 Signaling and Is Antagonized by Kit Ligand. *Cell Death Dis.* **2015**, *6*, e1688, doi:10.1038/cddis.2015.56.
334. Hamer, G.; Gell, K.; Kouznetsova, A.; Novak, I.; Benavente, R.; Höög, C. Characterization of a Novel Meiosis-Specific Protein within the Central Element of theSynaptonemal Complex. *J. Cell Sci.* **2006**, *119*, 4025, doi:10.1242/jcs.03182.
335. Hamer, G.; Wang, H.; Bolcun-Filas, E.; Cooke, H.J.; Benavente, R.; Höög, C. Progression of Meiotic Recombination Requires Structural Maturation of the CentralElement of the Synaptonemal Complex. *J. Cell Sci.* **2008**, *121*, 2445–2451, doi:10.1242/jcs.033233.
336. Hamer, G.; Wang, H.; Bolcun-Filas, E.; Cooke, H.J.; Benavente, R.; Höög, C. Progression of Meiotic Recombination Requires Structural Maturation of the CentralElement of the Synaptonemal Complex. *J. Cell Sci.* **2008**, *121*, 2445–2451, doi:10.1242/jcs.033233.
337. Hernández-Hernández, A.; Masich, S.; Fukuda, T.; Kouznetsova, A.; Sandin, S.; Daneholt, B.; Höög, C. The Central Element of the Synaptonemal Complex in Mice IsOrganized as a Bilayered Junction Structure. *J. Cell Sci.* **2016**, *129*, 2239, doi:10.1242/jcs.182477.
338. Hammond, G.L. Diverse Roles for Sex Hormone-Binding Globulin in Reproduction.
339. *Biol. Reprod.* **2011**, *85*, 431–441, doi:10.1095/biolreprod.111.092593.
340. Wood, M.A.; Mukherjee, P.; Toocheck, C.A.; Walker, W.H. Upstream Stimulatory Factor Induces Nr5a1 and Shbg Gene Expression during the Onset of Rat Sertoli Cell Differentiation. *Biol. Reprod.* **2011**, *85*, 965–976, doi:10.1095/biolreprod.111.093013.
341. Della-Maria, J.; Gerard, A.; Franck, P.; Gerard, H. Effects of Androgen-Binding Protein (ABP) on Spermatid Tnp1 Gene Expression in Vitro. *Mol. Cell. Endocrinol.***2002**, *198*, 131–141, doi:10.1016/s0303-7207(02)00376-3.
342. Herbert, Z.; Weigel, S.; Sendemir, E.; Marshall, A.; Caldwell, J.D.; Petrusz, P.; Peuckert, C.; Jirikowski, G.F. Androgen-Binding Protein Is Co-Expressed with Oxytocin in the Male Reproductive Tract. *Anat. Histol. Embryol.* **2005**, *34*, 286–293,doi:10.1111/j.1439-0264.2005.00605.x.
343. Gerard, H.; Gerard, A.; En Nya, A.; Felden, F.; Gueant, J.L. Spermatogenic Cells DoInternalize Sertoli Androgen-Binding Protein: A Transmission Electron Microscopy Autoradiographic Study in the Rat. *Endocrinology* **1994**, *134*, 1515–1527, doi:10.1210/endo.134.3.8119194.
344. Jeyaraj, D.A.; Grossman, G.; Weaver, C.; Petrusz, P. Dynamics of Testicular Germ Cell Proliferation in Normal Mice and Transgenic Mice Overexpressing Rat Androgen-Binding Protein: A Flow Cytometric Evaluation. *Biol. Reprod.* **2002**, *66*, 877–885, doi:10.1095/biolreprod66.4.877.
345. Morimoto, H.; Kanatsu-Shinohara, M.; Orwig, K.E.; Shinohara, T. Expression andFunctional Analyses of Ephrin Type-A Receptor 2 in Mouse Spermatogonial Stem Cells†. *Biol. Reprod.* **2020**, *102*, 220–232, doi:10.1093/biolre/ioz156.

346. Wang, K.; Sturt-Gillespie, B.; Hittle, J.C.; Macdonald, D.; Chan, G.K.; Yen, T.J.; Liu,S.-T. Thyroid Hormone Receptor Interacting Protein 13 (TRIP13) AAA-ATPase Is a Novel Mitotic Checkpoint-Silencing Protein. *J. Biol. Chem.* **2014**, *289*, 23928–23937,doi:10.1074/jbc.M114.585315.
347. Roig, I.; Dowdle, J.A.; Toth, A.; de Rooij, D.G.; Jasin, M.; Keeney, S. Mouse TRIP13/PCH2 Is Required for Recombination and Normal Higher-Order ChromosomeStructure during Meiosis. *PLoS Genet.* **2010**, *6*, doi:10.1371/journal.pgen.1001062.
348. Pacheco, S.; Marcet-Ortega, M.; Lange, J.; Jasin, M.; Keeney, S.; Roig, I. The ATMSignaling Cascade Promotes Recombination-Dependent Pachytene Arrest in Mouse Spermatocytes. *PLoS Genet.* **2015**, *11*, e1005017–e1005017, doi:10.1371/journal.pgen.1005017.
349. Li, X.C.; Li, X.; Schimenti, J.C. Mouse Pachytene Checkpoint 2 (Trip13) Is Requiredfor Completing Meiotic Recombination but Not Synapsis. *PLoS Genet.* **2007**, *3*, e130,doi:10.1371/journal.pgen.0030130.
350. Ma, H.T.; Poon, R.Y.C. TRIP13 Regulates Both the Activation and Inactivation of theSpindle-Assembly Checkpoint. *Cell Rep.* **2016**, *14*, 1086–1099, doi:10.1016/j.celrep.2016.01.001.
351. Clairmont, C.S.; Sarangi, P.; Ponnienselvan, K.; Galli, L.D.; Csete, I.; Moreau, L.; Adelmant, G.; Chowdhury, D.; Marto, J.A.; D'Andrea, A.D. TRIP13 Regulates DNARepair Pathway Choice through REV7 Conformational Change. *Nat. Cell Biol.* **2020**,*22*, 87–96, doi:10.1038/s41556-019-0442-y.
352. Luedtke, C.C.; McKee, M.D.; Cyr, D.G.; Gregory, M.; Kaartinen, M.T.; Mui, J.; Hermo, L. Osteopontin Expression and Regulation in the Testis, Efferent Ducts, and Epididymis of Rats during Postnatal Development through to Adulthood. *Biol. Reprod.***2002**, *66*, 1437–1448, doi:10.1095/biolreprod66.5.1437.
353. Yoo, J.K.; Lim, J.J.; Ko, J.J.; Lee, D.R.; Kim, J.K. Expression Profile of GenesIdentified in Human Spermatogonial Stem Cell-like Cells Using Suppression Subtractive Hybridization. *J. Cell. Biochem.* **2010**, *110*, 752–762, doi:10.1002/jcb.22588.
354. Acquaviva, L.; Székvölgyi, L.; Dichtl, B.; Dichtl, B.S.; de La Roche Saint André, C.;Nicolas, A.; Géli, V. The COMPASS Subunit Spp1 Links Histone Methylation to Initiation of Meiotic Recombination. *Science* **2013**, *339*, 215–218,doi:10.1126/science.1225739.
355. Sommermeyer, V.; Béneut, C.; Chaplais, E.; Serrentino, M.E.; Borde, V. Spp1, a Member of the Set1 Complex, Promotes Meiotic DSB Formation in Promoters by Tethering Histone H3K4 Methylation Sites to Chromosome Axes. *Mol. Cell* **2013**, *49*,43–54, doi:10.1016/j.molcel.2012.11.008.
356. Adam, C.; Guérois, R.; Citarella, A.; Verardi, L.; Adolphe, F.; Béneut, C.; Sommermeyer, V.; Ramus, C.; Govin, J.; Couté, Y.; et al. The PHD Finger Protein Spp1 Has Distinct Functions in the Set1 and the Meiotic DSB Formation Complexes.*PLoS Genet.* **2018**, *14*, e1007223, doi:10.1371/journal.pgen.1007223.
357. Karányi, Z.; Halász, L.; Acquaviva, L.; Jónás, D.; Hetey, S.; Boros-Oláh, B.; Peng, F.;Chen, D.; Klein, F.; Géli, V.; et al. Nuclear Dynamics of the Set1C Subunit Spp1 Prepares Meiotic Recombination Sites for Break Formation. *J. Cell Biol.* **2018**, *217*, 3398–3415, doi:10.1083/jcb.201712122.
358. Perozzi, G.; Barilá, D.; Plateroti, M.; Sambuy, Y.; Nobili, F.; Gaetani, S. Effects of Retinoids on Gene Expression in Different Epithelial Models in Vivo and in Vitro. *Z.Ernahrungswiss.* **1998**, *37 Suppl 1*, 29–34.
359. Faraonio, R.; Galdieri, M.; Colantuoni, V. Cellular Retinoic-Acid-Binding-Protein andRetinol-Binding-Protein MRNA Expression in the Cells of the Rat Seminiferous Tubules and Their Regulation by Retinoids. *Eur. J. Biochem.* **1993**, *211*, 835–842, doi:10.1111/j.1432-1033.1993.tb17616.x.
360. Eskild, W.; Troen, G.; Blaner, W.S.; Nilsson, A.; Hansson, V. Evidence for Independent Control at the MRNA and Protein Levels of Cellular Retinol BindingProtein 1 in Rat Sertoli Cells. *J. Reprod. Fertil.* **2000**, *119*, 101–109, doi:10.1530/jrf.0.1190101.
361. Domenjoud, L.; Kremling, H.; Burfeind, P.; Maier, W.M.; Engel, W. On the Expression of Protamine Genes in the Testis of Man and Other Mammals. *Andrologia* **1991**, *23*, 333–337, doi:10.1111/j.1439-0272.1991.tb02575.x.
362. Carrell, D.T.; Emery, B.R.; Hammoud, S. Altered Protamine Expression and Diminished Spermatogenesis: What Is the Link? *Hum. Reprod. Update* **2007**, *13*, 313–327, doi:10.1093/humupd/dml057.
363. Wykes, S.M.; Nelson, J.E.; Visscher, D.W.; Djakiew, D.; Krawetz, S.A. Coordinate Expression of the PRM1, PRM2, and TNP2 Multigene Locus in Human Testis. *DNA Cell Biol.* **1995**, *14*, 155–161, doi:10.1089/dna.1995.14.155.
364. Culp, B.R.; Lands, W.E.; Lucches, B.R.; Pitt, B.; Romson, J. The Effect of Dietary Supplementation of Fish Oil on Experimental Myocardial Infarction. *Prostaglandins* **1980**, *20*, 1021–1031, doi:10.1016/0090-6980(80)90056-8.
365. Pattabiraman, D.; Roelens, B.; Woglar, A.; Villeneuve, A.M. Meiotic RecombinationModulates the Structure and Dynamics of the Synaptonemal Complex during C. Elegans Meiosis. *PLoS Genet.* **2017**, *13*, e1006670–e1006670, doi:10.1371/journal.pgen.1006670.
366. Brandt, J.N.; Hussey, K.A.; Kim, Y. Spatial and Temporal Control of Targeting Polo-like Kinase during Meiotic Prophase. *J. Cell Biol.* **2020**, *219*, e202006094, doi:10.1083/jcb.202006094.

367. Persson, H.; Ayer-Le Lievre, C.; Soder, O.; Villar, M.; Metsis, M.; Olson, L.; Ritzen,M.; Hokfelt, T. Expression of Beta-Nerve Growth Factor Receptor MRNA in SertoliCells Downregulated by Testosterone. *Science* **1990**, *247*, 704, doi:10.1126/science.2154035.
368. Perrard, M.-H.; Vigier, M.; Damestoy, A.; Chapat, C.; Silandre, D.; Rudkin, B.B.;Durand, P. β-Nerve Growth Factor Participates in an Auto/Paracrine Pathway of
369. Regulation of the Meiotic Differentiation of Rat Spermatocytes. *J. Cell. Physiol.* **2007**,
370. *210*, 51–62, doi:10.1002/jcp.20805.
371. Wang, Y.; Zhu, T.; Li, Q.; Liu, C.; Han, F.; Chen, M.; Zhang, L.; Cui, X.; Qin, Y.; Bao,S.; et al. Prmt5 Is Required for Germ Cell Survival during Spermatogenesis in Mice. *Sci. Rep.* **2015**, *5*, 11031, doi:10.1038/srep11031.
372. Zhu, J.; Zhang, D.; Liu, X.; Yu, G.; Cai, X.; Xu, C.; Rong, F.; Ouyang, G.; Wang, J.;Xiao, W. Zebrafish Prmt5 Arginine Methyltransferase Is Essential for Germ Cell Development. *Dev. Camb. Engl.* **2019**, *146*, doi:10.1242/dev.179572.
373. Holloway, J.K.; Booth, J.; Edelmann, W.; McGowan, C.H.; Cohen, P.E. MUS81 Generates a Subset of MLH1-MLH3-Independent Crossovers in Mammalian Meiosis.*PLoS Genet.* **2008**, *4*, e1000186, doi:10.1371/journal.pgen.1000186.
374. Wechsler, T.; Newman, S.; West, S.C. Aberrant Chromosome Morphology in HumanCells Defective for Holliday Junction Resolution. *Nature* **2011**, *471*, 642–646, doi:10.1038/nature09790.
375. Wang, X.; Wang, H.; Guo, B.; Zhang, Y.; Gong, Y.; Zhang, C.; Xu, H.; Wu, X. Gen1 and Eme1 Play Redundant Roles in DNA Repair and Meiotic Recombination in Mice. *DNA Cell Biol.* **2016**, *35*, 585–590, doi:10.1089/dna.2015.3022.
376. Fleming, J.C.; Tartaglini, E.; Kawatsuji, R.; Yao, D.; Fujiwara, Y.; Bednarski, J.J.; Fleming, M.D.; Neufeld, E.J. Male Infertility and Thiamine-Dependent Erythroid Hypoplasia in Mice Lacking Thiamine Transporter Slc19a2. *Mol. Genet. Metab.* **2003**,*80*, 234–241, doi:10.1016/S1096-7192(03)00141-0.
377. Oishi, K.; Barchi, M.; Au, A.C.; Gelb, B.D.; Diaz, G.A. Male Infertility Due to GermCell Apoptosis in Mice Lacking the Thiamin Carrier, Tht1. A New Insight into the Critical Role of Thiamin in Spermatogenesis. *Dev. Biol.* **2004**, *266*, 299–309, doi:10.1016/j.ydbio.2003.10.026.
